# Inhibiting menin attenuates high-fat diet–induced weight gain by limiting intestinal lipid absorption in mice

**DOI:** 10.1172/JCI202709

**Published:** 2026-08-11

**Authors:** Xiaoru Cao, Pingping Zhou, Haiyue Meng, Zhitao Guo, Yan Cao, Chenghao Wang, Lulu Liu, Yinghao Guo, Yue Wang, Guoshun Xin, Dabin Liu, Feng Geng, Jian Ma

**Affiliations:** 1 Department of Pancreatobiliary Surgery, The Second Affiliated Hospital of Harbin Medical University and Department of Immunology, School of Basic Medical Sciences, Harbin Medical University, Harbin, China.; 2 Central Laboratory, Yulin Hospital, The First Affiliated Hospital of Xi’an Jiaotong University, Yulin, China.; 3 Department of Endocrinology, First Affiliated Hospital of Harbin Medical University, Harbin, China.; 4 NHC Key Laboratory of Molecular Probes and Targeted Diagnosis and Therapy, The Fourth Affiliated Hospital of Harbin Medical University, Harbin, China.; 5 Science and Technology Research Center, Harbin Medical University Cancer Hospital, Harbin, China.; 6 Key Laboratory of Molecular Oncology of Heilongjiang Province, Harbin, China.

**Keywords:** Gastroenterology, Metabolism, Obesity

## Abstract

Intestinal lipid metabolism is essential for systemic energy homeostasis, and its modulation is emerging as a therapeutic strategy for obesity. Menin, encoded by the *MEN1* gene, is a scaffold protein that regulates chromatin remodeling and gene expression and is abundantly expressed in intestinal epithelial cells (IECs), but its metabolic role remains underexplored. Here, we generated IEC-specific *Men1*-knockout mice and found that *Men1* deficiency protected against high-fat diet–induced obesity, accompanied by elevated carboxylesterase 1 (CES1) expression in IECs. Increased CES1 promoted triglyceride (TG) hydrolysis and reduced intracellular TG storage, thereby limiting the lipid substrate pool required for ApoB48-dependent chylomicron assembly. Although lipid hydrolysis was enhanced, steady-state free fatty acid levels were not increased; instead, *Men1* deficiency activated fatty acid β-oxidation programs and increased etomoxir-sensitive fatty acid–dependent mitochondrial respiration, supporting enhanced fatty acid catabolism. Mechanistically, menin recruited histone deacetylase 1 and interacted with the nuclear receptor liver X receptor-β to suppress *Ces1g* transcription, thereby sustaining efficient intestinal lipid absorption. Pharmacological inhibition of menin with MI-463 recapitulated the metabolic effects of inducible *Men1* deletion. In a human gut organoid-on-chip system, MI-463 dose-dependently increased CES1 expression and markedly reduced lipid accumulation. Collectively, our findings identify menin as a regulator of intestinal lipid metabolism and suggest menin inhibition as a potential therapeutic strategy for obesity-related metabolic disorders.

## Abstract 2

<strong>Targeting menin in intestinal cells boosts lipid breakdown, curbs absorption, and prevents obesity.</strong>

## Graphical abstract

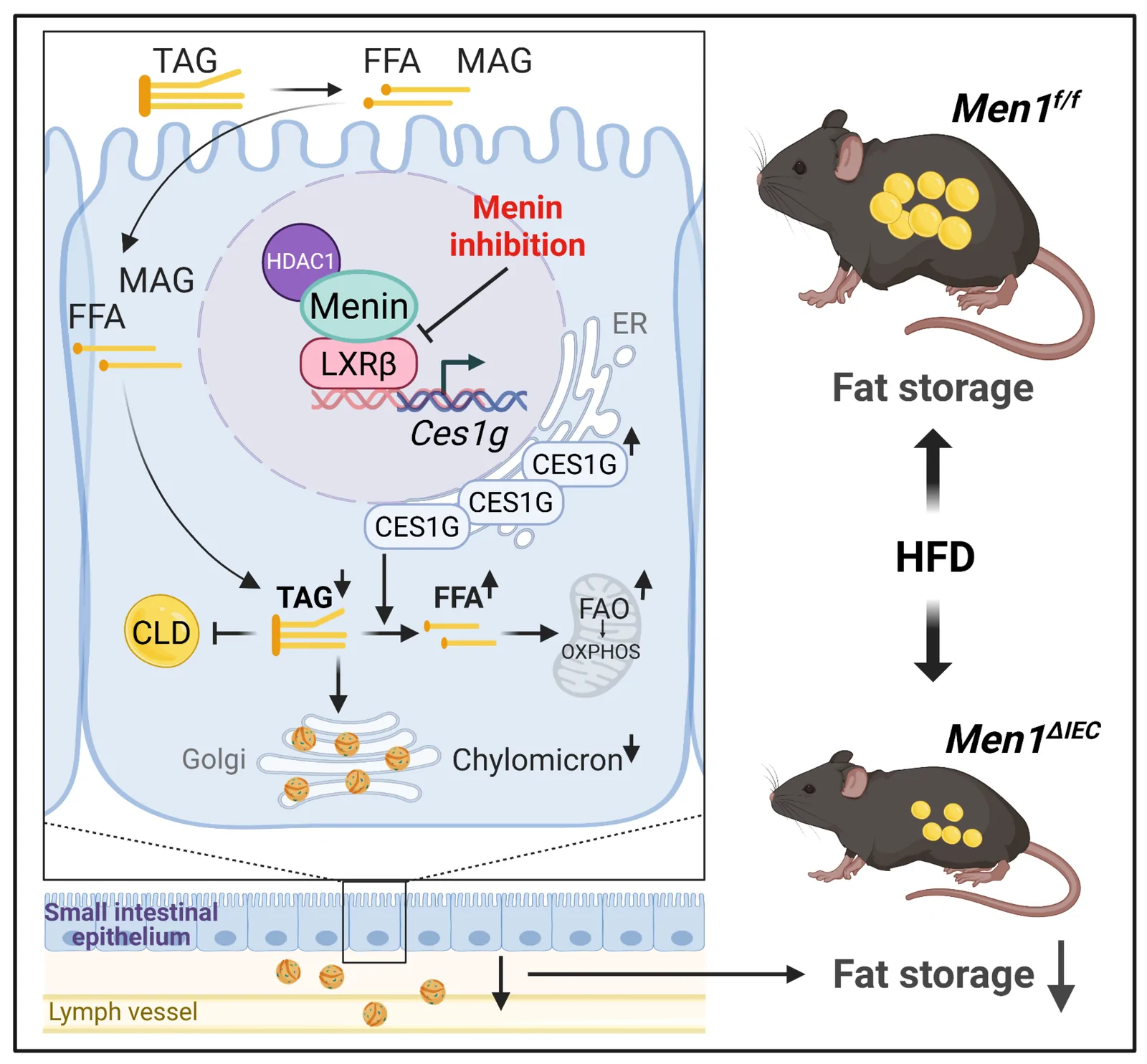

## Introduction

Obesity is widely recognized by WHO as a leading health challenge of the 21st century (1). Its pathogenesis is driven by chronic energy imbalance, with the small intestine serving as the principal site for dietary lipid absorption and thus being central to obesity development (2). In intestinal epithelial cells (IECs), dietary fats are taken up, reesterified into triglycerides (TGs), and packaged into chylomicrons for secretion. Under conditions of excess energy intake, lipid-absorptive processes facilitate adipose tissue expansion and ultimately contribute to systemic metabolic dysregulation — a hallmark event in obesity pathogenesis (3). Given the intestine’s pivotal role, strategies to restrain its absorptive capacity have attracted considerable interest.

Pharmacological and genetic interventions targeting intestinal lipid absorption have emerged as promising antiobesity approaches that do not require altered food intake or energy expenditure. For example, *Mogat2* ablation delays lipid absorption and protects against weight gain (4), while MTTP inhibition reduces chylomicron (CM) assembly and lipid export (5, 6), although systemic MTTP blockade is limited by resulting hepatic lipid accumulation (7). These observations underscore the therapeutic potential of targeting intestinal lipid metabolism; however, compared with the abundant machinery that promotes lipid uptake and transport, most endogenous lipases and genes responsible for lipid hydrolysis are expressed at low levels or are epigenetically silenced in the intestinal epithelium (8–10), posing a major challenge for simply enhancing catabolic pathways in the gut.

Among the enzymes involved in lipid catabolism, the carboxylesterase family — particularly CES1 — can hydrolyze TGs (11, 12), but its intestinal expression is low (13). Notably, hepatic overexpression of human CES1 enhances TG hydrolysis, promotes fatty acid oxidation (FAO), and protects against obesity and steatosis (14, 15). This raises the possibility that derepressing intestinal CES1 could enhance local lipid turnover and limit systemic lipid delivery. However, the upstream regulator of intestinal CES1 remains unknown, and its druggability is unclear.

Menin, a protein encoded by the *MEN1* gene, is a nuclear scaffold protein initially identified as a tumor suppressor in multiple endocrine neoplasia type 1 (MEN1) syndrome (16). Beyond its canonical role in tumor suppression, menin orchestrates a variety of metabolic pathways through physical interactions with transcription factors and chromatin‑modifying enzymes (17). In the liver, menin interacts with PPARγ and sirtuin 1 to regulate FAO and lipid transport, whereas in pancreatic β cells, it modulates proliferation and glucose–lipid homeostasis (18, 19). Despite its abundant expression in the intestine, whether menin influences intestinal lipid absorption and metabolism has remained unexplored. To address this gap, we hypothesized that menin might act as a negative regulator of intestinal lipolysis and set out to test this possibility using an in vivo loss‑of‑function mouse model.

In the present study, we generated IEC-specific *Men1*-knockout mice and subjected them to high-fat diet (HFD) feeding to evaluate menin’s impact on lipid absorption and obesity. We found that *Men1* deletion reduces adiposity by enhancing CES1-mediated TG hydrolysis and impairing CM formation. Liberated fatty acids are efficiently channeled into mitochondrial β-oxidation, not accumulated. Furthermore, pharmacological inhibition with menin inhibitor MI-463 upregulates CES1-dependent hydrolysis and ameliorates obesity in HFD-fed mice. Collectively, these findings identify a menin/CES1 axis governing intestinal lipid metabolism and suggest that targeting menin may offer a promising therapeutic strategy for obesity-related diseases.

## Results

### IEC-specific Men1 deletion attenuates HFD-induced weight gain in mice.

To investigate the physiological role of menin in the intestinal epithelium, we generated IEC-specific *Men1*-deficient mice (*Men1^ΔIEC^*) by crossing *Men1^fl/fl^* mice, in which *loxP* sites flank exons 3–8 of the *Men1* gene, with *Villin*-Cre transgenic mice (Supplemental Figure 1, A and B; supplemental material available online with this article; https://doi.org/10.1172/JCI202709DS1). PCR genotyping confirmed the *Men1^fl/fl^* allele and *Villin*-Cre transgene in *Men1^ΔIEC^* mice (Supplemental Figure 1C). To assess *Men1* deletion efficiency in IECs, we first prepared single-cell suspensions from intestinal tissues and confirmed by flow cytometry that IECs comprised approximately 90% of the isolated intestinal cell population (Supplemental Figure 1D). By genotyping and qPCR and Western blotting (WB) analyses, we confirmed efficient *Men1* deletion in IECs of *Men1^ΔIEC^* mice (Supplemental Figure 1E). By immunohistochemistry (IHC), we found that menin was predominantly expressed in IECs of wild-type mice, whereas its expression was diminished in *Men1^ΔIEC^* mice compared with littermate *Men1^fl/fl^* controls (Supplemental Figure 1F). Furthermore, *Men1* mRNA expression was lower in the proximal jejunum and colon of *Men1^ΔIEC^* mice compared with *Men1^fl/fl^* controls, while no changes were observed in other metabolic organs, such as the kidney, stomach, liver, or pancreas (Supplemental Figure 1G).

To assess the metabolic phenotypes of the mice, both male and female *Men1^fl/fl^* and *Men1^ΔIEC^* mice were fed a normal chow diet (NCD) or an HFD starting at 6 weeks of age and maintained on the assigned diet for 12 weeks (Figure 1A). Body weight was recorded weekly, and samples were collected in weeks 16 and 18 for metabolic analyses and tissue collection (Figure 1B). Compared with NCD feeding, HFD feeding increased body weight in both male and female mice (Figure 1, B and C, and Supplemental Figure 1H). However, HFD-induced body weight gain was more pronounced and sustained in male mice than in female mice. Therefore, subsequent metabolic and mechanistic analyses were performed in male mice to ensure a robust obesity-related phenotype. Of note, in male mice, no significant difference in body weight was observed between *Men1^fl/fl^* and *Men1^ΔIEC^* mice under NCD conditions (Figure 1B). However, under HFD feeding conditions, *Men1^ΔIEC^* mice gained less weight compared with *Men1^fl/fl^* mice (Figure 1, B and C), with marked reductions in adipose depot weights (Figure 1, D and E). Histological analysis revealed that compared with *Men1^fl/fl^* mice, *Men1^ΔIEC^* mice exhibited a smaller adipocyte size in both gWAT and rWAT depots (Figure 1, F and G), and Oil Red O staining demonstrated less hepatic lipid accumulation (Figure 1, H and I), consistent with decreased hepatic TG content (Figure 1J), in the *Men1^ΔIEC^* mice compared with the controls. Consistent with reduced lipid-associated metabolic burden, *Men1^ΔIEC^* mice displayed significantly lower fasting serum TG levels (Figure 1K) after 12 weeks of HFD feeding, while fasting serum cholesterol levels were unchanged as compared with controls (Supplemental Figure 2A). In addition, *Men1^ΔIEC^* mice exhibited lower fasting blood glucose (Supplemental Figure 2B), lower fasting insulin levels (Supplemental Figure 2C), and improved glucose tolerance (Supplemental Figure 2, D and E) compared with *Men1^fl/fl^* mice. These findings suggest that intestinal menin deficiency markedly mitigates HFD-induced obesity and lipid-associated metabolic dysfunction.

To investigate the mechanisms underlying the improved metabolic phenotypes in the *Men1^ΔIEC^* mice, we examined intestinal lipid absorption and metabolism-related parameters. No significant differences were observed between genotypes in small or large intestinal lengths (Supplemental Figure 2F), jejunal circumference (Supplemental Figure 2G), or villus lengths (Supplemental Figure 2H). Intestinal permeability was also unchanged, as assessed by FITC-dextran assay and *ZO-1* mRNA expression in IECs (Supplemental Figure 2, I and J). Similarly, genes related to bile acid transport (*Asbt*, *Osta*, and *Ostb*) and pancreatic lipases (*Plinp* and *Clps*) were expressed at comparable levels between groups (Supplemental Figure 2K). These findings indicate that intestinal morphology and classical mechanisms of lipid digestion and absorption were not affected by *Men1* deletion.

To assess whether altered energy expenditure contributed to the lean phenotype, we performed metabolic cage studies (20). In line with the reduced adiposity, *Men1^ΔIEC^* mice showed significantly lower fat mass and fat-to-body weight ratio compared with *Men1^fl/fl^* controls (Supplemental Figure 2, L and M). However, there were no differences in the respiratory exchange ratio and total energy expenditure between *Men1^fl/fl^* and *Men1^ΔIEC^* mice during the full-day, dark-phase, and light-phase periods (Supplemental Figure 2, N–Q). Under HFD feeding, cumulative food intake was also similar between genotypes (Figure 1L). Notably, the food efficiency, which is defined as the body weight gain per unit of food consumed, was significantly lower in *Men1^ΔIEC^* mice compared with *Men1^fl/fl^* mice (Figure 1M). Measurements of fecal lipid and TG contents further showed no differences between genotypes (Figure 1, N and O), indicating that the differences in weight gain were not attributable to malabsorption or altered fecal lipid loss. Collectively, these results suggest that the reduced weight gain in *Men1^ΔIEC^* mice on HFD is not due to changes in food intake, digestion, or total energy expenditure.

### Men1 deletion in IECs reduces CM formation.

Having excluded systemic alterations in energy balance, we next investigated whether the reduced weight gain in HFD-fed *Men1^ΔIEC^* mice is linked to impaired intracellular processing and transport of absorbed lipids within IECs. After 6 weeks of HFD feeding, *Men1^fl/fl^* and *Men1^ΔIEC^* mice were orally gavaged with olive oil (10 μL/g body weight) (Figure 2A), and serum TG levels were measured at 0, 2, 4, and 6 hours after gavage (Figure 2B). *Men1^ΔIEC^* mice displayed significantly lower postprandial serum TG levels than *Men1^fl/fl^* controls (Figure 2, B and C). To determine whether this reduction reflected enhanced peripheral TG clearance or decreased intestinal lipid delivery, we employed tyloxapol, a lipoprotein lipase (LPL) inhibitor, to block TG uptake by peripheral tissues, before olive oil gavage (18). The reduction in serum TG levels in *Men1^ΔIEC^* mice persisted despite tyloxapol administration, indicating that the lower TG levels in these mice were not attributable to enhanced peripheral TG clearance (Figure 2, D–F). Because tyloxapol also allows circulating TG derived from hepatic VLDL secretion to accumulate, we further assessed hepatic VLDL output. Plasma VLDL-TG levels measured at 0, 1, 2, and 3 hours were comparable between *Men1^fl/fl^* and *Men1^ΔIEC^* mice (Supplemental Figure 3A), suggesting that reduced hepatic VLDL production was unlikely to account for the lower serum TG levels in *Men1^ΔIEC^* mice.

To evaluate whether the reduction in postprandial circulating TG levels stemmed from impaired intestinal lipid export, we assessed multiple related parameters. First, serum FFA levels after olive oil gavage were lower in *Men1^ΔIEC^* mice compared with controls (Figure 2G). Second, Nile Red staining of intestinal tissues revealed reduced lipid droplet accumulation in the IECs of *Men1^ΔIEC^* mice (Figure 2, H and I). Consistently, direct quantification of TG content in IECs showed a significant reduction in TG accumulation in *Men1^ΔIEC^* mice (Figure 2J).

The reduced intracellular TG accumulation in IECs of *Men1^ΔIEC^* mice (Figure 2J) prompted us to examine whether the biogenesis of CMs, the principal carriers of intestinal lipid export, was qualitatively altered. In IECs, resynthesized TGs are packaged by MTTP and assembled with ApoB48 into CMs for lymphatic secretion. Because CM diameter is a major determinant of lipid cargo (21), we next assessed CM size by transmission electron microscopy. This analysis revealed that CM diameter was markedly smaller in *Men1^ΔIEC^* mice (203.02 ± 5.19 nm [mean ± SD]) compared with *Men1^fl/fl^* controls (284.48 ± 12.9 nm [mean ± SD]) (Figure 2, K and L). Correspondingly, we also found that the serum levels of ApoB48, a key structural component of CMs, were also significantly lower in *Men1^ΔIEC^* mice (Figure 2, M and N), further supporting the notion that postprandial CM assembly and lipid export are impaired in the absence of menin in IECs. Together, these data indicate that IEC-specific *Men1* deletion leads to reduced serum lipid levels in HFD-fed mice by impairing CM assembly and limiting dietary lipid entry into the circulation.

### Men1 deletion upregulates Ces1g expression in IECs, promoting lipolysis and curbing lipid export.

To elucidate the mechanism underlying reduced CM production and postprandial lipid entry into the circulation in *Men1^ΔIEC^* mice, we performed transcriptomic profiling of proximal small IECs from HFD-fed *Men1^fl/fl^* and *Men1^ΔIEC^* mice. Differential expression analysis revealed 563 upregulated and 372 downregulated genes in *Men1^ΔIEC^* IECs compared with *Men1^fl/fl^* controls (Figure 3A). Gene ontology (GO) enrichment analysis showed that the upregulated genes were primarily associated with biological processes (BP), such as fatty acid metabolism, lipid transport, and lipid catabolism, and were enriched in cellular components (CC), like apical plasma membrane, brush border, and lipoprotein particles (Figure 3B). Notably, genes involved in TG uptake and synthesis (*Slc27a1*, *Slc27a2*, *Mogat2*, *Dgat1*, *Dgat2*) and neutral TG lipases (*Atgl*, *Hsl*, *Mgll*) were unchanged, multiple carboxylesterase family genes (*Ces1c*, *Ces1d*, *Ces1g*, *Ces2a*, *Ces2b*) were significantly upregulated, and lipid packaging–related genes (*Fabp1*, *Fabp2*, *Mttp*) were downregulated (Figure 3C). RT-qPCR analysis of primary IECs showed robust upregulation of several *Ces* family genes in *Men1^ΔIEC^* mice, with *Ces1g* exhibiting the most prominent induction (Figure 3D). In contrast, genes involved in fatty acid uptake, TG synthesis, and cholesterol metabolism were largely unchanged, except for increased *Cd36*, whereas the lipid transport/CM-associated genes *Fabp1* and *Fabp2*, but not *Mttp*, were reduced (Supplemental Figure 3, B–D).

Because *Cd36* expression was increased after *Men1* depletion, we next assessed whether this change enhanced early fatty acid uptake. Using a BODIPY 500/510 C1, C12–based fluorescent fatty acid uptake assay in MODE-K cells, we found that *Men1* knockdown did not increase fatty acid uptake at 5, 10, 20, or 30 minutes compared with control cells (Supplemental Figure 3E). We therefore focused on the *Ces1* family, which showed the most consistent transcriptional induction. Consistent with the RNA-seq and RT-qPCR data, WB analysis confirmed substantially increased CES1 protein levels in jejunal epithelial cells from *Men1^ΔIEC^* mice compared with controls (Figure 3, E and F). Given that *Ces1g* exhibited the strongest induction among the *Ces* family genes, *Ces1g* was selected for downstream mechanistic studies in mice.

To assess whether *Ces1g* upregulation depends on HFD feeding, we examined CES1 protein levels in jejunal epithelial cells under NCD conditions. CES1 expression was markedly increased in IECs from *Men1*-deficient mice under NCD feeding (Supplemental Figure 3, F and G). These results indicate that CES1 induction is primarily driven by IEC *Men1* deletion rather than by HFD feeding. We next examined the functional impact of CES1G on intracellular lipid metabolism by overexpressing *Ces1g* in MODE-K cells (Figure 3G). Following oleic acid treatment, Nile Red staining and TG quantification showed that *Ces1g* overexpression was associated with significantly less intracellular droplet accumulation and TG content (Supplemental Figure 3, H and I, and Figure 3H). Because reduced TG storage alone cannot establish enhanced lipid hydrolysis, we used an NBD-TG–based lipid hydrolysis assay to determine whether CES1G directly promotes TG hydrolysis in IECs. The NBD-C6 standard curve showed a linear relationship between fluorescence intensity and NBD-C6 abundance, enabling quantification of FFA release (Supplemental Figure 3J). Although steady-state FFA levels were not significantly altered in primary IECs from *Men1^ΔIEC^* mice (Supplemental Figure 3K), *Ces1g* overexpression induced a robust, time-dependent increase in FFA release in MODE-K cells (Figure 3I). Together, these data indicate that CES1G directly enhances TG hydrolysis in IECs, thereby contributing to reduced intracellular TG accumulation.

To further validate the essential role of CES1 in *Men1* deficiency–induced lipid catabolism and reduced CM output, we knocked down *CES1* in *MEN1*-deficient CACO-2 cells (Supplemental Figure 3L). We then established a Transwell-based human CACO-2 monolayer model to study lipid absorption and transport (Supplemental Figure 3M), which we validated using key indicators: fluorescein permeability < 2 × 10^–6^ cm/s (22), transepithelial electrical resistance > 400 Ω·cm² (23), and apical-to-basal alkaline phosphatase activity ratio > 2 (24) (Supplemental Figure 3, N–P). By lipid transport assays we found that *MEN1* knockdown led to reduced intracellular lipid accumulation and apical-to-basal lipid transport, both of which were significantly restored by additional *CES1* knockdown (Supplemental Figure 3, Q and R). Similarly, treatment of *Men1*-deficient MODE-K cells with WWL113, a selective inhibitor of CES1 and its murine orthologs Ces3/Ces1f, restored intracellular lipid content (Supplemental Figure 3, S–U). These findings indicate that reduced lipid accumulation and transport after *MEN1* depletion were CES1 dependent.

To determine whether increased CES1 activity contributes to impaired intestinal lipid transport in *Men1^ΔIEC^* mice, we inhibited CES1 with WWL113 after 6 weeks of HFD feeding (25) (Figure 3J). WWL113 significantly reduced intestinal CES1 esterase activity in both genotypes (Figure 3K). Importantly, WWL113 increased postprandial serum TG levels and rescued the reduced TG response in *Men1^ΔIEC^* mice (Figure 3L). It also restored intestinal lipid droplet accumulation and ApoB48 expression in *Men1^ΔIEC^* mice (Figure 3, M–P). In addition, as a direct measure of lipolytic activity, NBD-TG hydrolysis assays showed that *Men1* knockdown increased FFA release in a time-dependent manner, and this increase was partially suppressed by WWL113 treatment (Supplemental Figure 3V). Collectively, these in vitro and in vivo findings demonstrate that *Ces1g* and *CES1* are critical downstream effectors of *Men1/MEN1* deletion in IECs, mediating enhanced lipid hydrolysis, reduced lipid droplet accumulation, and decreased dietary lipid entry into the bloodstream.

### Men1 deficiency enhances FAO capacity in IECs.

As *Ces1g* upregulation promoted TG hydrolysis without causing a detectable increase in steady-state FFA levels in IECs, we next asked whether *Men1* deficiency enhances downstream fatty acid utilization. Gene set enrichment analysis (GSEA) of intestinal epithelial transcriptomes revealed enrichment of pathways related to mitochondrial fatty acid β-oxidation, PPARα-regulated lipid metabolism, and peroxisomal lipid metabolism in *Men1^ΔIEC^* IECs (Figure 4A), suggesting the activation of lipid-catabolic programs. Consistently, RT-qPCR analysis showed that several FAO-related genes, including *carnitine palmitoyltransferase 1A (Cpt1a)*, *Acox1*, *Acadl*, and *Ehhadh*, were significantly upregulated in *Men1^ΔIEC^* IECs, whereas *Acadm* showed a modest but nonsignificant increase (Figure 4B). WB analysis further confirmed efficient menin depletion and increased CPT1A expression in *Men1^ΔIEC^* IECs (Figure 4C).

To directly assess β-oxidation capacity, we measured metabolic activity in isolated IECs. *Men1^ΔIEC^* IECs displayed significantly higher oxygen consumption rate (OCR) and increased FAO activity compared with *Men1^fl/fl^* controls (Figure 4, D and E). In contrast, citrate synthase (CS) activity was unchanged between the 2 groups (Figure 4F), indicating that the enhanced FAO capacity was not simply due to increased mitochondrial abundance. We further validated the FAO phenotype in MODE-K cells. *Men1* knockdown significantly increased *Ces1g* and *Cpt1a* expression under both BSA control and oleic acid–treated conditions (Figure 4, G and H). In palmitate oxidation stress assays, exogenous palmitate increased OCR in MODE-K cells, whereas the CPT1 inhibitor etomoxir attenuated this response, confirming the assay’s specificity (Figure 4, I and J). Importantly, *Men1*-knockdown MODE-K cells exhibited higher basal and maximal OCR when palmitate was supplied as the substrate, and this increase was suppressed by etomoxir treatment (Figure 4, K and L), indicating that *Men1* deficiency promotes fatty acid–dependent mitochondrial respiration. Together, these results demonstrate that *Men1* deficiency activates FAO-related transcriptional programs and directly enhances fatty acid–dependent mitochondrial respiration in IECs, thereby facilitating the utilization of FFAs generated by CES1-mediated TG hydrolysis.

### The menin–HDAC1–LXRβ complex cooperatively represses Ces1g/CES1 transcription.

To determine whether menin regulates *Ces1g/CES1* transcription, we first examined *Ces1g* mRNA levels in *Men1*-knockdown MODE-K cells. *Men1* knockdown significantly increased *Ces1g* mRNA and CES1 protein levels, whereas *Men1* overexpression suppressed CES1 protein expression (Figure 5, A and B). We observed similar results in *MEN1*-knockdown CACO-2 cells, where both CES1 mRNA and protein levels were upregulated (Supplemental Figure 4, A and B) and were reduced upon *MEN1* overexpression (Supplemental Figure 4B). Together with the upregulated *Ces1g* mRNA and CES1 protein levels observed in primary IECs of *Men1^ΔIEC^* mice (Figure 3E), these findings from both mouse and human intestinal cells confirm that menin negatively regulates *Ces1g/CES1* transcription.

To explore the underlying mechanism, we performed chromatin immunoprecipitation followed by sequencing (ChIP-seq) in mouse IECs. Menin was enriched at the *Ces1g* promoter but not at the *Ces1c* or *Ces1d* promoters, with only weak enrichment near the *Ces2a* promoter (Figure 5C and Supplemental Figure 4C). ChIP-qPCR at 3 candidate *Ces2a* promoter regions showed no significant menin enrichment (Supplemental Figure 4D), indicating that *Ces2a* is unlikely to be directly regulated by menin in this context. ChIP-qPCR screening covering the *Ces1g* promoter identified 2 regions (–426 to –658 bp and –1,921 to –2,286 bp) with specific menin enrichment, whereas adjacent sites, including –1,800 and –904 bp, showed no significant binding (Supplemental Figure 4E). Focusing on these 2 regions, we confirmed that menin occupancy was markedly reduced in *Men1^ΔIEC^* mice compared with *Men1^fl/fl^* controls, which was accompanied by increased RNA polymerase II recruitment (Figure 5, D and E) and correlated with an enhanced *Ces1g* transcription. Similar patterns were observed in human CACO-2 cells (Supplemental Figure 4F). In addition, dual-luciferase reporter assays further confirmed that *Men1*/*MEN1* overexpression suppressed *Ces1g* and *CES1* promoter activity (Figure 5F and Supplemental Figure 4G).

Because menin acts a scaffold protein that cooperates with transcription factors to coregulate lipid metabolic genes, we used transcription factor binding site predictions and literature mining (26–28) to identify 5 candidate transcription factors that potentially interact with menin to regulate *CES1*: HNF4α, LXRα, LXRβ, PPARγ, and RELA (Figure 5G). Co-immunoprecipitation (Co-IP) assays showed that menin interacts with HNF4α, LXRα, LXRβ, and PPARγ (Figure 5H). Dual-luciferase assays indicated that among these, LXRβ and RELA significantly enhanced *CES1* promoter activity (Figure 5I). Notably, in *MEN1*-knockdown HEK293T cells, LXRβ exhibited an even greater effect in promoting *Ces1g* transcription, suggesting that menin may antagonize LXRβ-mediated *CES1* transcription (Figure 5J).

As LXRβ regulates gene expression through LXR response elements (LXREs) (29), we next identified 2 conserved LXREs in the promoter regions of *Ces1g* and *CES1* (Supplemental Figure 4, H and I) by using the JASPAR database. Of note, mutation of these LXREs diminished LXRβ-mediated transcriptional activation of *Ces1g* and *CES1* in *Men1/MEN1*-knockdown cells (Figure 5K and Supplemental Figure 4J), supporting direct regulation by LXRβ. To further support this, we analyzed publicly available RNA-seq data (NCBI GEO, accession no. GSE167983) from mouse IECs treated with low-dose GW3965, a selective LXR agonist (30). Our analysis showed that LXR activation upregulated *Ces1* and *Ces2* expression in vivo (Supplemental Figure 5A). Conversely, treatment of MODE-K and CACO-2 cells with the LXR antagonist GSK2033 resulted in a dose-dependent suppression of *Ces1g/CES1* mRNA and protein levels (Supplemental Figure 5, B–G). GSK2033 also reversed *Ces1g* mRNA (Figure 5L) and CES1 protein levels (Supplemental Figure 5, H and I) in *Men1*-deficient MODE-K cells, further supporting the notion that menin suppresses LXRβ-driven *Ces1g* transcription. To determine whether *Men1* deficiency broadly activates canonical LXR signaling, we examined representative LXR-responsive genes involved in cholesterol efflux and lipid metabolism. *Abca1*, *Abcg1*, *Acaca*, *Srebf1*, *Srebf2*, *Scd1*, *Acly*, and *Fasn* were not significantly changed in IECs from *Men1^ΔIEC^* compared with *Men1^fl/fl^* controls (Supplemental Figure 5J). These results suggest that *Men1* deficiency selectively enhanced *Ces1g* transcription without globally activating canonical LXR-responsive lipid metabolic programs.

Because menin can recruit chromatin-modifying enzymes to modulate chromatin accessibility at target gene loci (31), we next examined whether *Men1* deletion affects histone acetylation at the *Ces1g* locus. ChIP-seq revealed increased in H3 acetylation (H3Ac) at the *Ces1g* regulatory region in *Men1^ΔIEC^* IECs (Figure 5, M and N), suggesting that menin represses transcription by maintaining a closed chromatin state. Consistently, co-IP experiments showed that LXRβ formed a complex with menin, HDAC1, and HDAC3 (Figure 5O). ChIP-qPCR analysis further revealed that *Men1^ΔIEC^* mice exhibited increased H3Ac and H3 acetylation at lysine 27 (H3K27Ac) levels at the *Ces1g* promoter, along with reduced HDAC1 occupancy, while HDAC3 and LXRβ binding remained unchanged (Figure 5P and Supplemental Figure 5K). Re-ChIP assays confirmed the simultaneous presence of menin, HDAC1, and LXRβ at the *Ces1g* promoter, supporting a cooperative repression mechanism (Figure 5Q). Collectively, these findings suggest that LXRβ recruits the menin–HDAC1 complex to the *Ces1g* promoter, leading to reduced histone acetylation and transcriptional repression of *Ces1g* (Figure 5R).

### Pharmacological or genetic inhibition of intestinal menin attenuates HFD-induced weight gain in mice.

To evaluate the therapeutic potential of targeting menin in obesity, we generated a tamoxifen-inducible (TAM-inducible) IEC-specific *Men1*-knockout mouse model (*Men1^ΔIEC-IND^*). Mice were fed an HFD starting at 6 weeks of age, followed by oral gavage with TAM at 12 weeks to induce *Men1* deletion, and analyzed at 19 weeks (Figure 6A). WB analysis confirmed successful deletion of menin in the proximal intestinal epithelium of TAM-treated *Men1^ΔIEC-IND^* mice, which was accompanied by a marked increase in CES1 protein levels (Figure 6B). Under HFD conditions, *Men1^ΔIEC-IND^* mice gained significantly less weight than *Men1^fl/fl^* controls (Figure 6C). FTT further showed significantly lower postprandial serum TG levels in *Men1^ΔIEC-IND^* mice (Figure 6, D and E), and Nile Red staining revealed decreased lipid droplet accumulation in the intestinal epithelium after olive oil gavage (Figure 6, F and G). These results indicate that inducible *Men1* deletion in IECs upregulates CES1 expression and mitigates HFD-induced lipid absorption and weight gain.

Given that genetic ablation of *Men1* could attenuate HFD-induced weight gain in mice, we investigated whether pharmacological inhibition of menin using MI-463 (32) recapitulates the metabolic benefits of genetic deletion. In MODE-K and CACO-2 cells, treatment with the menin inhibitor MI-463 dose-dependently increased *Ces1g*/*CES1* mRNA and CES1 protein levels (Figure 6, H and I, and Supplemental Figure 6, A and B), suggesting that MI-463 activates the menin/CES1 axis. Co-IP assays revealed that MI-463 dose-dependently disrupted the interaction between menin and LXRβ (Figure 6J). ChIP-qPCR analysis showed that MI-463 treatment significantly reduced menin and HDAC1 binding at the *Ces1g* promoter, while increasing H3K27Ac enrichment but not affecting LXRβ binding (Figure 6K and Supplemental Figure 6C). These data support a model whereby MI-463 derepresses *Ces1g/CES1* transcription by disrupting the menin–HDAC1–LXRβ complex.

To evaluate the in vivo efficacy of MI-463 in treating obesity, wild-type mice were fed on HFD for 6 weeks and then orally administered MI-463 at 30 mg/kg once daily or vehicle for an additional 6 weeks (Figure 6L). MI-463 treatment significantly attenuated HFD-induced weight gain compared with vehicle treatment (Figure 6M). Tissue gene expression analysis revealed that MI-463 specifically upregulated *Ces1g* in the intestine but not in the liver (Figure 6N), confirming its targeted action on intestinal lipid metabolism. In FTT assays, MI-463–treated mice exhibited lower serum TG levels (Figure 6, O and P), reduced intestinal lipid accumulation (Figure 6, Q and R), and decreased serum ApoB48 levels (Figure 6S), indicating attenuated CM formation and lipid absorption. Moreover, MI-463 treatment significantly decreased fasting insulin levels (Supplemental Figure 6D), improved glucose tolerance (Supplemental Figure 6, E and F), and enhanced insulin sensitivity (Supplemental Figure 6, G and H), suggesting a beneficial impact on glucose metabolism alongside obesity reduction. In addition, H&E staining revealed no obvious pathological changes in the heart, liver, spleen, jejunum, lung, or kidney (Supplemental Figure 6I). Serum alanine aminotransferase (ALT), aspartate aminotransferase (AST), creatinine (CRE), blood urea nitrogen (BUN), and uric acid (UA) levels were also comparable between vehicle- and MI-463–treated mice (Supplemental Figure 4, J–N), indicating no detectable liver or kidney toxicity. Collectively, these findings demonstrate that pharmacological inhibition of menin by MI-463 suppresses intestinal lipid export and substantially attenuates HFD-induced obesity and associated metabolic disturbances in mice.

### MI-463 administration enhances CES1 expression and reduces lipid accumulation in human small intestinal organoids.

To explore the translational relevance of CES1 in human intestinal lipid metabolism, we first analyzed proteins involved in intestinal fatty acid absorption and transport. We found high expression of enzymes responsible for fatty acid reesterification (MOGAT2, DGAT1) and CM assembly (MTTP, FABP2) while the lipolytic enzyme CES1 was expressed at low levels (Supplemental Figure 7A). These findings suggest that the human intestine predominantly favors lipid reesterification and packaging over intracellular lipolysis for lipid processing.

To validate whether menin inhibition enhances CES1 expression and modulates lipid metabolism in human intestinal epithelium, we employed a high-throughput intestinal barrier organ-on-chip system (IBAC-on-chip), derived from human small intestinal organoids (Figure 7A). Immunofluorescence staining confirmed expression of the intestinal differentiation markers VILLIN, MUC2, and CHGA, validating the intestinal identity of the organoid-derived epithelial barrier (Figure 7B). In the presence of oleic acid stimulation, MI-463 treatment dose-dependently increased CES1 mRNA expression and resulted in a dose-dependent upregulation of *CES1* as measured by RT-qPCR (Figure 7C). Immunofluorescence analysis and quantitative imaging confirmed that MI-463 treatment significantly increased CES1 protein expression (Figure 7, D and E), supporting the capacity of menin inhibition to enhance the expression of this noncanonical lipase in human IECs. Furthermore, upon incubation with the fluorescent fatty acid analog BODIPY-C12, organoids treated with MI-463 displayed markedly reduced intracellular lipid staining, indicating a significant decrease in lipid accumulation within IECs (Figure 7, F and G). Collectively, these findings demonstrate that MI-463 upregulates CES1 expression and reduces lipid accumulation in a human organoid-derived intestinal epithelium model, further supporting the role of menin inhibition in modulating human intestinal metabolism.

## Discussion

Obesity and related metabolic disorders are major public health challenges, and safe, effective antiobesity therapies remain urgently needed. Current treatments mainly reduce energy intake or increase energy expenditure, but they often cause systemic side effects (33). Targeting intestinal lipid metabolism may offer a more tissue-specific strategy to limit dietary fat entry into the circulation. Although several approaches have been explored, their clinical use remains limited. For example, MTTP inhibitors are approved only for rare inherited disorders (34), and their inhibition may induce compensatory hepatic lipogenesis and steatosis. Enhancing intestinal lipase activity, particularly CES1-mediated hydrolysis of long-chain fatty acid esters, may therefore represent a promising strategy to promote intestinal lipid catabolism and reduce lipid transport (27). In this study, we identified menin as a suppressor of intestinal lipid degradation. Menin represses CES1 expression through an LXRβ-dependent epigenetic mechanism, maintaining a low-lipolytic state in IECs, promoting chylomicron formation, and contributing to systemic fat accumulation. Genetic deletion of *Men1* in IECs or pharmacological inhibition of menin with MI-463 relieves this repression, enhances intestinal lipid hydrolysis, reduces dietary lipid transport, and protects against obesity.

Menin has been implicated in hepatic cholesterol metabolism, pancreatic β cell function, and adipose tissue homeostasis (35). Although abundantly expressed in the intestinal epithelium, its role there has been poorly understood. Here, IEC-specific *Men1* deletion produced a clear antiobesity phenotype under HFD challenge, including reduced weight gain, decreased white adipose and hepatic lipid accumulation, and improved glucose tolerance. *Men1* deletion did not disrupt intestinal morphology, permeability, or bile acid circulation, but it reduced intracellular lipid accumulation and postprandial serum TG levels. These findings suggest menin mainly regulates intracellular processing of absorbed lipids. During absorption, FFAs and monoglycerides are taken up by enterocytes, reesterified into TGs, and assembled into CMs (36). Our data indicate that *Men1* deficiency redirects absorbed fatty acids away from TG storage and CM assembly and toward FAO, thereby limiting dietary lipid delivery to the circulation. Notably, although *Men1* deficiency enhanced IEC FAO, indirect calorimetry did not detect increased whole-body energy expenditure. This is not necessarily contradictory: enhanced IEC FAO likely reflects a shift in intracellular lipid fate rather than global metabolic increase. Because absorption is highly dynamic postprandially, *Men1* deficiency may increase FAO mainly after feeding, and this localized change may be insufficient to alter 24-hour energy expenditure. Compensatory adjustments in other organs may also buffer systemic expenditure. Consistent with this, enhanced FAO in specific tissues does not necessarily increase total energy expenditure (37, 38). Thus, the antiobesity phenotype is more attributable to remodeling of IEC lipid handling than to increased whole-body energy expenditure.

CES1 is a key lipase in hepatic lipoprotein metabolism (14). Hepatic *Ces1* overexpression protects against steatohepatitis (15), but its intestinal function is less characterized (39). Previous studies showed CES1G deficiency causes abnormal CM composition and postprandial hyperlipidemia (40). In our study, *Men1* deletion induced CES1G expression, reduced IEC lipid droplets, and decreased serum ApoB48, suggesting CES1G limits CM production by promoting intracellular lipid hydrolysis. This was associated with upregulated FAO, raising the possibility that liberated FFAs are preferentially oxidized rather than reesterified. While CES1G is predominantly ER/microsomal (13) and β-oxidation occurs in mitochondria and peroxisomes, these spatially separated processes may be linked through intracellular lipid flux. In the NBD-TG assay, *Ces1g* overexpression increased FFA release, supporting a direct role in TG hydrolysis. Released fatty acids may serve as oxidative substrates or activate fatty acid–responsive transcriptional programs, consistent with reports that lipolytic products can activate PPARα/δ signaling (41, 42). Because mammalian β-oxidation occurs in both mitochondria and peroxisomes (41, 43, 44), CES1G likely promotes FAO indirectly by increasing fatty acid availability rather than by directly catalyzing β-oxidation.

Mechanistically, menin has been reported to regulate cholesterol metabolism in colorectal cancer by repressing ABCA1 and ABCG1 through LXREs (45). Here, we show menin directly interacts with LXRβ and recruits HDAC1 to LXRE sites in the *CES1* promoter. Co-IP and ChIP-qPCR supported formation of a menin–LXRβ–HDAC1 complex, though its structural basis remains undefined. Our data indicate menin suppresses LXRβ-mediated *CES1* transcription via HDAC1 recruitment, while *Men1* deletion increases H3K27Ac and CES1 expression. Given menin participates in multiple epigenetic complexes, future studies should examine whether other epigenetic enzymes are involved (46). Inducible *Men1* deletion ameliorated HFD-induced weight gain and postprandial hyperlipidemia, indicating menin-mediated CES1G repression is therapeutically targetable.

Our findings further support menin as a druggable regulator. In HFD-fed mice, menin inhibitor MI-463 reduced weight gain, improved glycemic control, and lowered circulating TG levels. Mechanistically, MI-463 disrupted the menin–LXRβ interaction and derepressed *CES1* transcription, suggesting pharmacological menin inhibition improves metabolism by enhancing intracellular lipid processing. MI-463 is a potent, orally bioavailable menin–MLL inhibitor with antileukemic activity and favorable pharmacokinetics (47). In our model, 6 weeks of treatment caused no overt abnormalities in serum markers or organ histology, indicating the biosafety of the menin inhibitor. The translational potential is also supported by clinical development of other menin inhibitors: Revumenib showed a manageable safety profile in a phase I trial and has been FDA-approved for KMT2A-rearranged acute leukemia (48); ziftomenib has also demonstrated clinical activity with manageable toxicity (49). These findings support the pharmacological tractability of menin in intestinal lipid regulation.

To further evaluate translational relevance, we used a human small intestinal organoid model. Unlike conventional cultures, organoids preserve epithelial polarity, cellular heterogeneity, and crypt–villus architecture (50). Most importantly, MI-463 treatment increased CES1 expression and enhanced lipid degradation in human intestinal epithelium, suggesting menin-mediated repression is conserved in human IECs. Overall, consistent effects in mice and human organoids support cross-species relevance of the menin/CES1 axis.

Several limitations should be noted. First, *Villin-Cre* may affect a subset of enteroendocrine cells (51). Future studies using more specific drivers like *Chga*-Cre, combined with single-cell RNA-seq, may clarify cell type–specific roles. Second, gut microbiota are important regulators of metabolic homeostasis and obesity (52, 53), but we did not systematically analyze microbiota composition in *Men1^ΔIEC^* mice. 16S rRNA amplicon or metagenomic analyses are warranted in future studies.

In conclusion, this study identifies menin as a regulator of small intestinal lipid metabolism. Menin represses *CES1* transcription through the LXRβ/CES1 axis, promoting CM formation and dietary lipid entry into the circulation. Menin inhibition enhances TG hydrolysis, suppresses CM production in IECs, and protects against HFD-induced obesity. These findings provide mechanistic insight into intestinal lipid regulation and highlight the menin/CES1 axis as a potential therapeutic target for obesity and related metabolic disorders.

## Methods

### Sex as a biological variable.

Both male and female mice were included in the initial experiments to evaluate the effect of intestinal epithelial *Men1* deletion on HFD-induced obesity. Because male mice developed a more pronounced and sustained obesity phenotype than female mice, and the protective effect of *Men1* deletion was more evident in males, subsequent metabolic, mechanistic, and pharmacological experiments were performed in male mice to ensure a robust and reproducible obesity-associated phenotype. Whether the full metabolic effects of intestinal epithelial *Men1* deletion and menin inhibition are similar in female mice remains to be determined. Human small intestinal organoid experiments were performed using iPSC-derived intestinal epithelial models, and sex was not analyzed as an independent biological variable in these experiments.

### Reagents and resources.

A list of reagents and commercial assays, along with their catalog numbers, is provided in Supplemental Table 7.

### Mice.

All mice were on a C57BL/6J genetic background and housed in a specific pathogen–free facility at a constant temperature of 23°C under a 12-hour light/dark cycle. Euthanasia was performed by cervical dislocation after CO_2_ inhalation.

*Men1^fl/fl^* mice and wild-type C57BL/6J mice were purchased from Shanghai Model Organisms Center, Inc. (Shanghai, China). Intestinal epithelium-specific *Villin*-Cre and TAM-inducible *Villin*-CreERT2 transgenic mice were obtained from Cyagen Biosciences Inc. (Guangzhou, China). To generate intestinal epithelial cell–specific *Men1*-knockout mice (*Men1^ΔIEC^*), *Villin*-Cre mice were crossed with *Men1^fl/fl^* mice. The *Men1^ΔIEC^* mice and the *Men1^fl/fl^* control mice used in this study were littermates. For inducible knockout models (*Men1^ΔIEC-IND^*), *Villin*-CreERT2 mice were crossed with *Men1^fl/fl^* mice, and TAM was used to induce intestinal epithelial cell–specific *Men1* deletion. Genotyping was performed by PCR (primers listed in Supplemental Table 1). Mice aged 7–8 weeks were used for experiments, with 6–7 mice per group. The diets included an NCD and HFD (60% kcal from fat, D12492, Research Diets). For TAM administration, TAM (S1238, Selleck) was dissolved in a solution of 10% ethanol and 90% corn oil at a concentration of 20 mg/mL. The dose was calculated based on body weight (100 μL/10 g body weight, i.e., 5 mg per mouse). TAM was administered by oral gavage once daily for 5 consecutive days. To treat the mice with MI-463, MI-463 (S7816, Selleck) was dissolved in 0.5% CMC-Na in a sealed centrifuge tube to achieve a drug concentration of 4.5 mg/mL, with an ultrasonic water bath used to assist with dissolution. MI-463 was administered orally to the mice at a dose of 30 mg/kg once daily for 6 weeks. The dosage of MI-463 was determined based on previously published studies (54). To evaluate the short-term safety of MI-463, serum was collected for biochemical analysis of ALT, AST, CRE, BUN, and UA using commercial assay kits according to the manufacturers’ instructions. At the end of treatment, mice were euthanized, and major organs, including the heart, liver, spleen, jejunum, lung, and kidney, were collected, fixed in 4% paraformaldehyde, embedded in paraffin, sectioned, and stained with hematoxylin and eosin. For WWL113 treatment, after establishing the obese mouse model, WWL113 (HY-110148, MedChemExpress) was dissolved in a solution of 15% castor oil and 85% PBS at a concentration of 4.5 mg/mL. The dose was calculated based on body weight (30 mg/kg). WWL113 was administered via intraperitoneal injection once daily for 2 weeks. During treatment, body weight, serum lipid levels, and other indicators were monitored weekly.

### BODIPY 500/510 C1, C12 fatty acid uptake assay.

Early fatty acid uptake was assessed using BODIPY 500/510 C1, C12 (C2055-1mg, Biyun Tian Biotechnology Co., LTD). Control and *Men1*-knockdown MODE-K cells were seeded in 24-well plates and cultured in 10% FBS DMEM. Cells were washed with PBS and incubated in serum-free medium containing BODIPY-C16 (10 μM) according to the manufacturer’s protocol. After incubation for 0, 5, 10, 20, or 30 minutes at 37°C, cells were immediately washed 3 times with cold PBS to remove extracellular dye. Fluorescence intensity was measured using a fluorescence microplate reader or fluorescence microscope.

### NBD-TG–based lipid hydrolysis assay.

Lipid hydrolysis activity was measured using an NBD-TG–based fluorescence assay as previously described (55). Briefly, a 7:1 NBD-TG: phosphatidylcholine (PC) substrate emulsion containing phosphatidylinositol (PI) was prepared by mixing NBD-TG, PC, and PI in chloroform. The lipid mixture was dried under nitrogen, resuspended in buffer II, sonicated on ice, and subjected to ultracentrifugation at 200,000*g*. The emulsion-containing fraction was collected and used as the substrate for the hydrolysis assay. MODE-K cells overexpressing CES1G or control cells were lysed in ice-cold buffer K containing 1 mM Tris-HCl, 1 mM EGTA, and 1 mM MgCl_2_ with protease inhibitors. For each reaction, 100 μL cell lysate containing 100 μg total protein was incubated with 10 μL of 7:1 NBD-TG:PC/PI substrate emulsion at 37°C for 0, 15, 30, 60, and 90 minutes. In inhibition experiments, lysates were preincubated with WWL113 before substrate addition. Fluorescence was measured at excitation/emission wavelengths of 460/530 nm using a SpectraMax M5 microplate reader, and substrate-only background fluorescence was subtracted from all readings.

To quantify released NBD-C6 fatty acid, an NBD-C6 standard curve was prepared in parallel. NBD-C6 (2.94 mg) was dissolved in 1 mL of DMSO to obtain a 10 mM stock solution. The stock was diluted in buffer K to obtain standards corresponding to 1,250, 625, 312.5, 125, 31.25, and 12.5 pmol in 100 μL. Fluorescence intensities of the standards were measured under the same conditions, with background values from solutions containing no NBD subtracted. Lipid hydrolysis activity was calculated from the NBD-C6 standard curve and normalized to total protein content and incubation time.

### OCR assay.

OCR in primary IECs isolated from mice fed on HFD for 4 weeks was measured using the Extracellular OCR Plate Assay Kit (E297, Dojindo) according to the manufacturer’s instructions. Briefly, freshly isolated small IECs were resuspended in complete medium and seeded into a 96-well, black, clear-bottom microplate at an optimized density (typically 4 × 10^5^ cells/well for suspension cells). Cells were incubated with the oxygen probe working solution prepared by diluting the oxygen probe in serum-containing medium, followed by incubation at 37°C for 30 minutes to allow probe equilibration. Subsequently, 10 μL of medium or treatment solution was added to each well as required, and 1 drop of prewarmed mineral oil was overlaid to prevent oxygen exchange with ambient air. After a 5-minute incubation at 37°C, kinetic fluorescence measurements were performed using a microplate reader (excitation 500 nm, emission 650 nm) at 10-minute intervals for up to 200 minutes. OCR values were calculated from the slope of oxygen consumption over time using the manufacturer-provided analysis template and expressed as pmol/min per well.

### FAO assays.

FAO activity in primary small IECs was measured using a Fatty Acid Oxidation Colorimetric Assay Kit (E-BC-K784-M, Elabscience) according to the manufacturer’s protocol. Briefly, freshly isolated IECs were washed with cold PBS and homogenized in extraction working solution on ice. Cell lysates were centrifuged at 10,000*g* for 15 minutes at 4°C. Equal volumes of sample supernatant were incubated with chromogenic working solution in the presence or absence of substrate working solution at 37°C for 30 minutes. Absorbance was measured at 450 nm before and after incubation. FAO activity was calculated from the standard curve and normalized to total protein concentration, and results were expressed as U/g protein.

### Seahorse XF palmitate oxidation stress assay.

Palmitate-supported mitochondrial respiration was assessed using the Seahorse XF Palmitate Oxidation Stress Test Kit (103693-100, Agilent) on a Seahorse XF96 extracellular flux analyzer. MODE-K cells were seeded in XF96 cell culture microplates (1 × 10^4^ cells/well). After 24 hours, cells were incubated overnight in substrate-limited medium, and the hydration probe plate was hydrated overnight at 37°C in a non-CO_2_ incubator. On the day of analysis, cells were equilibrated in substrate-limited XF assay medium supplemented with l-carnitine (0.5 mM). XF Palmitate-BSA FAO substrate (5 mM) or BSA control was added immediately before measurement. Where indicated, etomoxir (0.4 μM) was used to inhibit CPT1-dependent FAO. OCR was measured under basal conditions and after sequential injection of oligomycin (1.5 μM), FCCP (1 μM), and rotenone/antimycin A (0.5 μM). OCR values were normalized to cell number, and etomoxir-sensitive palmitate-supported OCR was used as an index of mitochondrial FAO.

### CS activity assay.

CS activity in primary small IECs was measured using a Citrate Synthase Activity Assay Kit (Colorimetric; EEA054, Thermo Fisher Scientific) according to the manufacturer’s protocol. Freshly isolated IECs were washed with cold PBS, homogenized in kit extraction solution on ice, and centrifuged at 350*g* at 4°C to obtain clarified lysates. Equal amounts of protein were incubated with the reaction mixture containing acetyl-CoA, oxaloacetate, and chromogenic reagent in a 96-well plate. Absorbance at 412 nm was measured using a SpectraMax M5 from Molecular Devices, and CS activity was calculated from the change in absorbance and normalized to total protein concentration.

### Establishment and characterization of the human small intestinal organoid barrier model.

Human small intestinal organoids were thawed and expanded according to Beijing Daxiang Biotech’s internal standard operating procedure. Once sufficient material was obtained, the organoids were enzymatically dissociated and seeded onto the porous membranes of IBAC M1 chips to establish a human small intestinal epithelial barrier model. Cultures were maintained under dynamic flow conditions to promote epithelial differentiation and maturation. Barrier differentiation was assessed by immunofluorescence staining for intestinal lineage markers. The mature epithelium formed villus-like structures resembling those of the human small intestine.

### Assessment of CES1 expression and fatty acid uptake in the human small intestinal organoid barrier model.

For CES1 analysis, established barrier models were starved in DMEM/F12 basal medium for 3 hours and then treated for 24 hours under dynamic culture conditions with intestinal organoid medium containing oleic acid alone or oleic acid plus test compounds. Samples were washed with Dulbecco’s PBS (DPBS), fixed, stained for CES1, and analyzed by high-content imaging.

For fatty acid uptake analysis, barrier models were pretreated for 24 hours with test compounds or vehicle in differentiation medium, followed by 3 hours’ starvation in DMEM/F12. The apical compartment was then exposed to 5 μM BODIPY-C12 in DMEM/F12, whereas the basolateral compartment received dye-free DMEM/F12. After 4 hours, barriers were washed 3 times with DPBS, fixed with 4% paraformaldehyde, counterstained with DAPI, and analyzed by high-content imaging.

### Statistics.

Detailed information regarding sample sizes and the statistical analyses applied is provided in the corresponding figure legends. The number of animals used was consistent with standard practices in the field. All animals and samples that met the predefined experimental conditions were included in the analysis. All statistical data are presented as mean ± SD. Comparisons between 2 groups were performed using independent-sample 2-tailed Student’s *t* tests. For comparisons among multiple groups, 1-way ANOVA was used, and for 2-factor data, repeated measures 2-way ANOVA was applied. Multiple comparisons between groups were corrected using either Dunnett’s or Bonferroni’s post hoc test. All statistical analyses were performed using GraphPad Prism version 10.0. A *P* value of less than 0.05 was considered statistically significant. The statistical tests applied are specified in the corresponding figure legends. The graphical abstract was prepared using Biorender.com. Figures were prepared using GraphPad Prism 10, Origin 2022, and Adobe Illustrator 2022.

Detailed protocols are provided in the Supplemental Methods.

### Study approval.

All animal experiments in this study were conducted in accordance with the protocols approved by the Animal Care and Use Committee of Harbin Medical University (Case no: HMUIRB2025032).

### Data availability.

All data supporting the findings of this study are available within the article and its supplemental material. Supporting Data Values are provided as an XLS file. Mouse RNA-seq data were deposited in the NCBI Sequence Read Archive (SRA) under accession number PRJNA1502564. ChIP-seq raw data were deposited in the NCBI database under NCBI BioProject accession PRJNA1313781. Additional data are available from the corresponding author upon reasonable request.

## Author contributions

JM conceptualized and supervised the study; XC, LL, and YG prepared and maintained the *Men1*-knockout mice; XC, PZ, YC, and HM designed and performed the morphological and biochemical analyses; ZG and CW analyzed the RNA-seq and ChIP-seq data; GX and YW prepared the experimental materials; JM and XC drafted the initial manuscript; and PZ, DL, FG, and JM revised the manuscript. All authors reviewed and approved the final version of the manuscript.

## Conflict of interest

The authors have declared that no conflict of interest exists.

## Funding support

- National Natural Science Foundation of China (NSFC) grants: 82270904 (to JM) and 82300601 (to PZ).

## Supplementary Material

Supplemental data

Unedited blot and gel images

Supporting data values

## Figures and Tables

**Figure 1:**
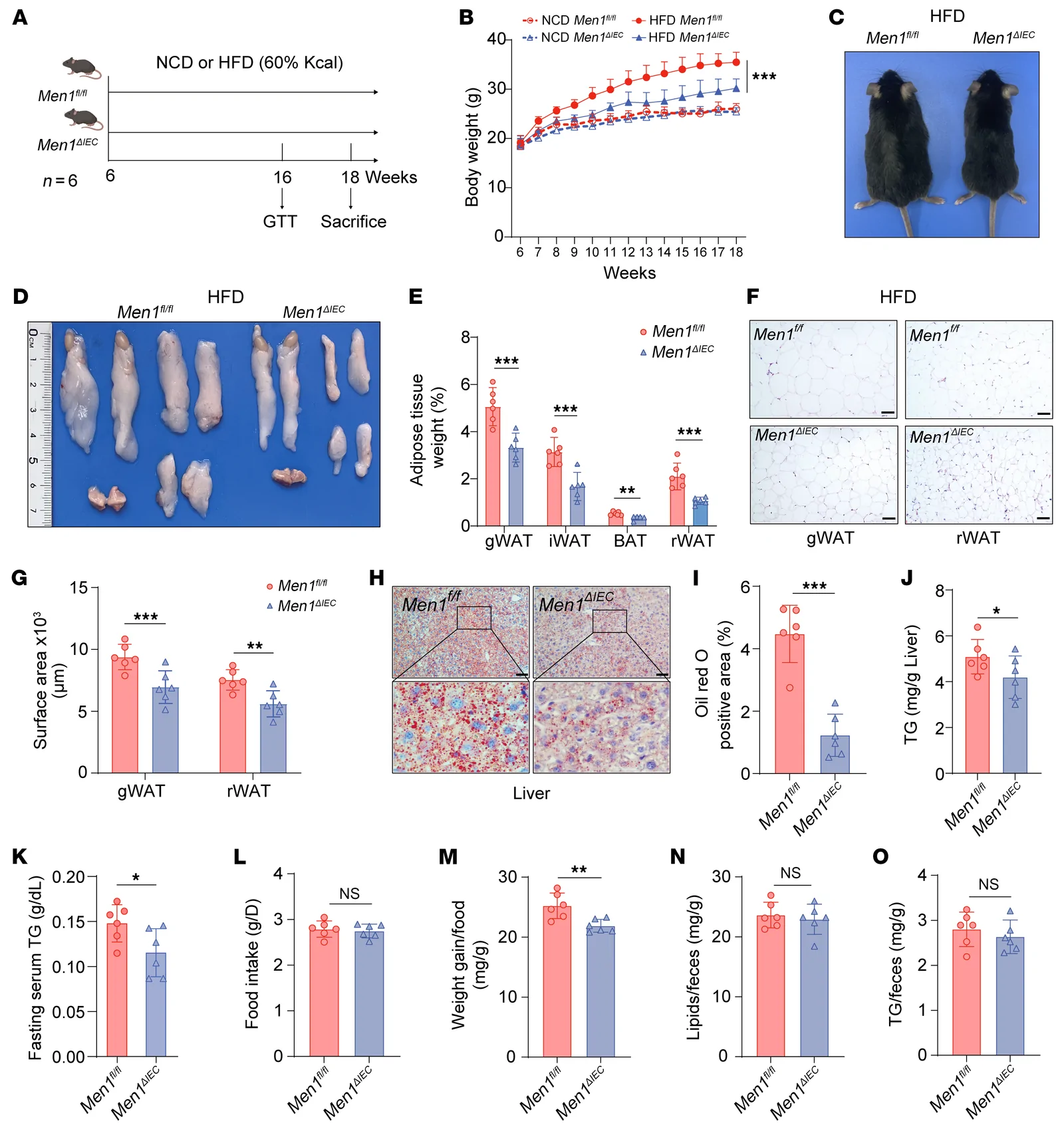
IEC-specific *Men1* deletion attenuates HFD-induced weight gain in mice. (**A**) Experimental design: 6-week-old *Men1^fl/fl^* and *Men1^ΔIEC^* mice were fed either an NCD or HFD. A glucose tolerance test (GTT) was performed at week 16, and mice were sacrificed at week 18. Samples were collected at weeks 12, 16, and 18 for RNA-seq, metabolic analyses, and histological assessments. (**B**) Body weight of *Men1^fl/fl^* and *Men1^ΔIEC^* mice under NCD or HFD feeding. Unfilled symbols, NCD; filled symbols, HFD. (**C**) Images of mice after 12 weeks of HFD feeding. (**D**) Images of gonadal white adipose tissue (gWAT), inguinal white adipose tissue (iWAT), retroperitoneal white adipose tissue (rWAT), and brown adipose tissue (BAT) following 12 weeks of HFD. (**E**) Tissue weight ratios of different fat depots to total body weight. (**F** and **G**) H&E staining (**F**) and quantification of average adipocyte area (**G**) in gWAT and rWAT. Scale bars: 100 μm. (**H** and **I**) Oil Red O staining (**H**) and quantification of Oil Red O–positive area (**I**) of hepatic lipid accumulation following 12 weeks of HFD feeding. Scale bars: 100 μm. (**J**) Hepatic TG content following 12 weeks of HFD. (**K**) Fasting serum TG levels after 12 weeks of HFD feeding. (**L**) Average daily food intake following 10 weeks of HFD feeding. (**M**) Food efficiency calculated as body weight gain per gram of food intake following 10 weeks of HFD feeding. (**N** and **O**) Lipid and TG content in feces. *n* = 6 mice per group. For quantitative analyses, each data point represents 1 mouse. All data are presented as mean ± SD. Statistical significance was assessed by 2-way ANOVA for panels **B**, **E**, and **G** and by unpaired 2-tailed *t* test for panels **I**–**O**. *P < 0.05, **P < 0.01, ***P < 0.001.

**Figure 2:**
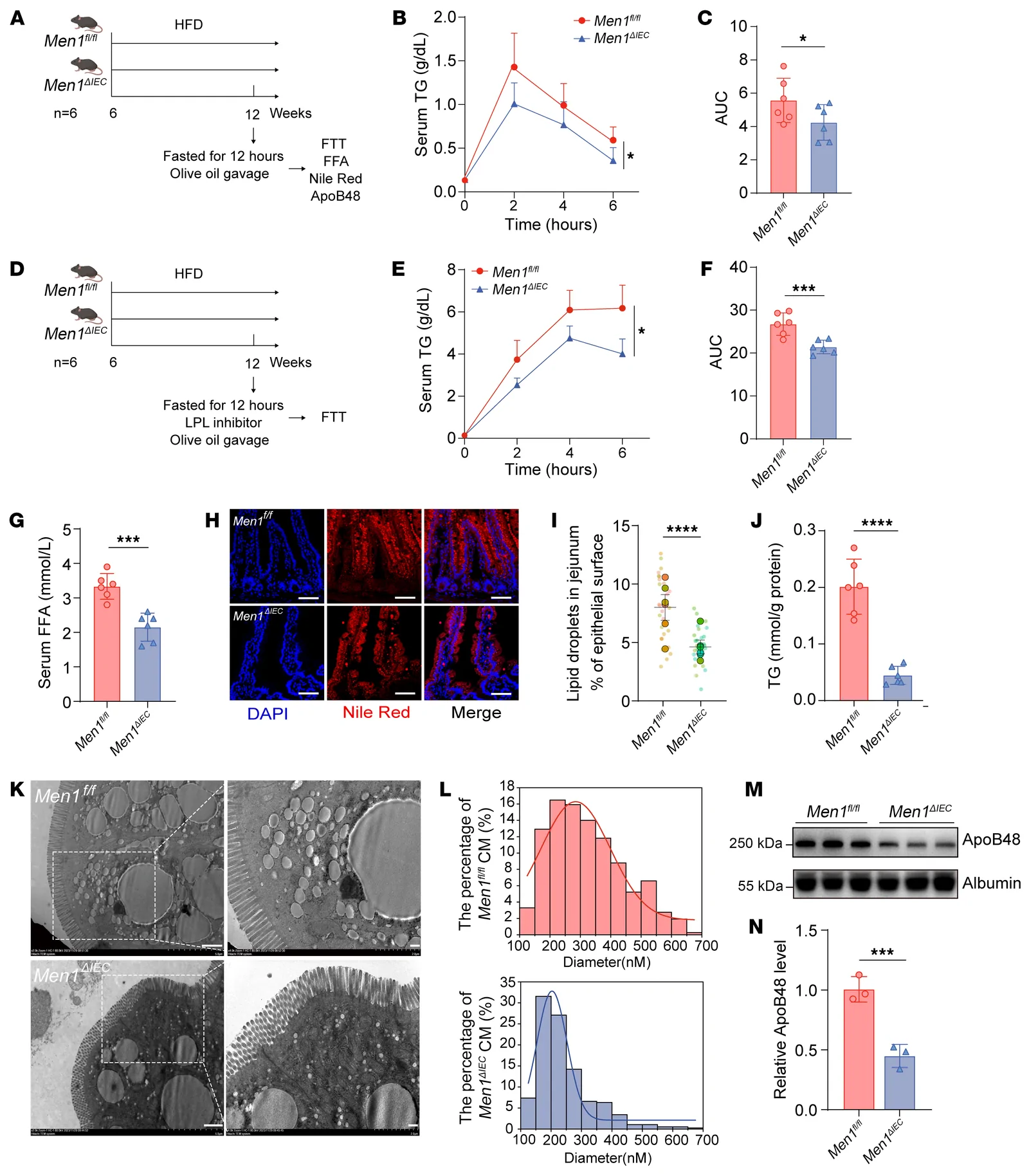
*Men1* deletion in IECs reduces chylomicron formation. (**A**) Experimental design: 6-week-old *Men1^fl/fl^* and *Men1^ΔIEC^* mice were fed an HFD for 6 weeks, fasted for 12 hours, and gavaged with olive oil (10 μL/g). Serum was collected at 0, 2, 4, and 6 hours and intestinal tissues at 6 hours. FTT, fat tolerance test; FFA, free fatty acid. (**B** and **C**) Time-course analysis of serum TG levels (**B**) and quantification of AUC (**C**). (**D**) After a 12-hour fast, mice were injected intravenously with tyloxapol (50 mg/mL, 10 μL/g) and gavaged with olive oil 15 minutes later. (**E** and **F**) Time-course TG levels (**E**) and AUC (**F**) in LPL-inhibited mice. (**G**) Serum FFA levels at 6 hours postgavage. (**H** and **I**) Nile Red staining of proximal small intestinal epithelial cells (**H**) and quantification of Nile Red–positive area (**I**). Five randomly selected fields per mouse were averaged as 1 data point. Scale bars: 50 μm. (**J**) TG content in proximal small intestine at 6 hours postgavage. (**K** and **L**) Transmission electron microscopy images and chylomicron diameter distributions (*n* = 3 mice per group). Ten fields per mouse were analyzed; 372 and 383 chylomicrons were measured in *Men1^fl/fl^* and *Men1^ΔIEC^* mice, respectively. Scale bars: 2 μm; inset scale bars: 400 nm. (**M** and **N**) WB and densitometric quantification of serum ApoB48 protein (*n* = 3 mice per group). Unless otherwise indicated, *n* = 6 mice per group. Each data point in **B**, **C**, **E**–**G**, **I**, **J**, and **N** represents 1 mouse. All data are presented as mean ± SD. Statistical significance was assessed by 2-way ANOVA for panels **B** and **E**; by unpaired 2-tailed Student’s *t* test for panels **C**, **F**, **G**, **I**, **J**, and **N**; and by Gaussian distribution analysis for panel **L**. *P < 0.05, ***P < 0.001, ****P < 0.0001.

**Figure 3:**
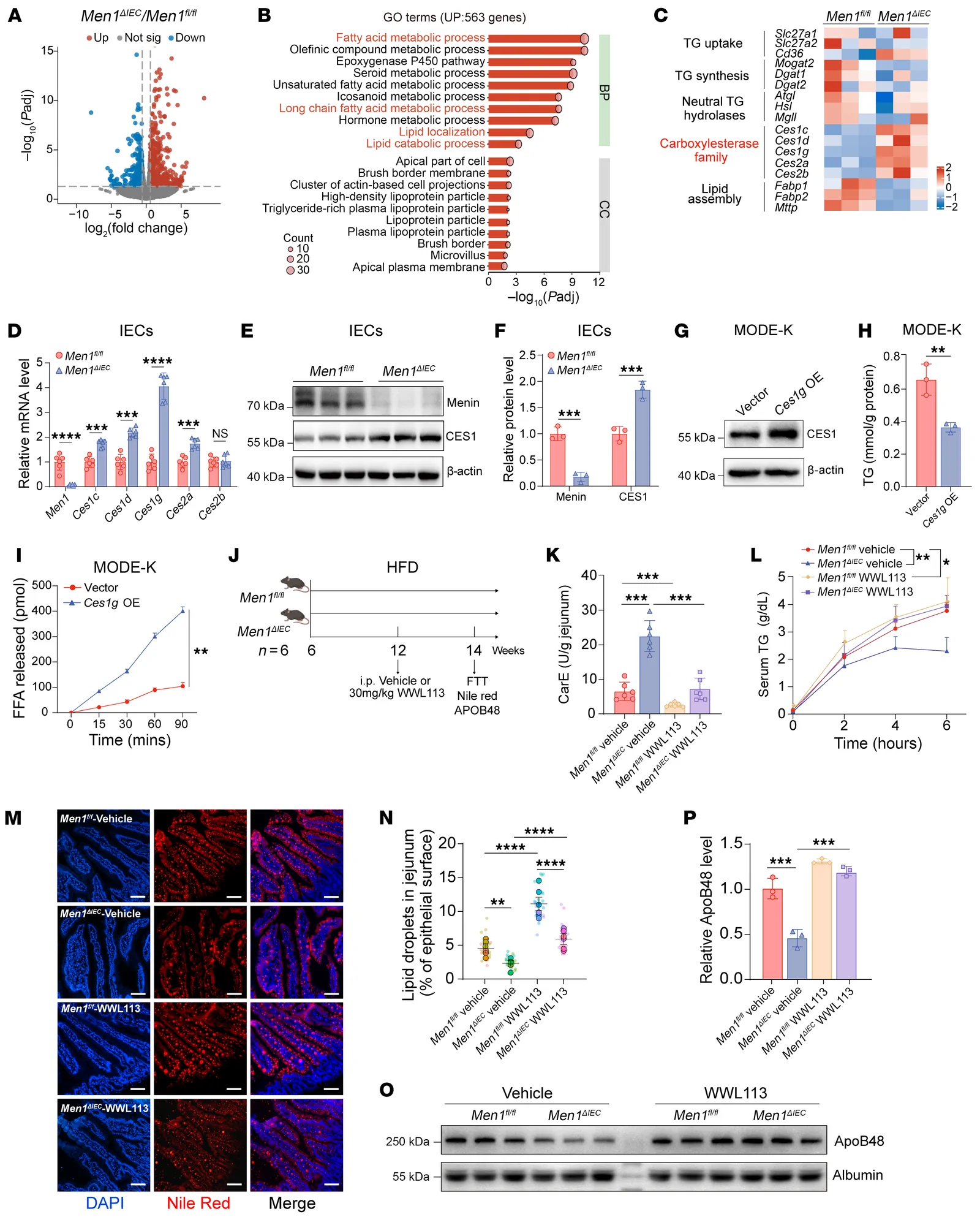
*Men1* deletion upregulates *Ces1g* expression in IECs, driving lipolysis and curbing lipid export. (**A**–**C**) Volcano plot of differentially expressed genes (**A**), GO enrichment analysis of upregulated genes (**B**), and heatmap of genes involved in intestinal lipid absorption, reesterification, lipolysis, and transport (**C**) in IECs from HFD-fed *Men1^ΔIEC^* vs. *Men1^fl/fl^*. (**D**) RT-qPCR analysis of the indicated genes in IECs after 4 weeks of HFD feeding. (**E** and **F**) WB (**E**) and densitometric quantification (**F**) of menin and CES1 in IECs. (**G** and **H**) Representative immunoblotting of CES1 (**G**) and intracellular TG content (**H**) in MODE-K cells transfected with *Ces1g* or control vector. (**I**) NBD-TG–based lipid hydrolysis assay in CES1G-overexpressing or control MODE-K cells. (**J**) Experimental design. *Men1^fl/fl^* and *Men1^ΔIEC^* mice were fed an HFD for 6 weeks and then treated with vehicle or WWL113 (30 mg/kg, i.p.) for 2 weeks before fat tolerance testing, Nile Red staining, and ApoB48 analysis. (**K**) Carboxylesterase activity in proximal small intestine. (**L**) Serum TG levels measured at 0, 2, 4, and 6 hours during FTT. (**M** and **N**) Nile Red staining and quantification of intestinal epithelial lipid accumulation. Five randomly selected fields per mouse were averaged as 1 data point. Scale bars: 50 μm. (**O** and **P**) WB analysis and densitometric quantification of serum ApoB48 at 6 hours after the test. For **A**–**C**, **E**, **F**, **O**, and **P**, *n* = 3 mice per group; for **D** and **K**–**N**, *n* = 6 mice per group; for **H** and **I**, *n* = 3; panel **G** shows representative results from 3 independent experiments. All data are presented as mean ± SD. Statistical significance was assessed by 2-way ANOVA for panels **D**, **F**, **I** and **L**; by unpaired 2-tailed Student’s *t* test for panel **H**; and by 1-way ANOVA for panels **K**, **N**, and **P**. *P < 0.05, **P < 0.01, ***P < 0.001, ****P < 0.0001.

**Figure 4:**
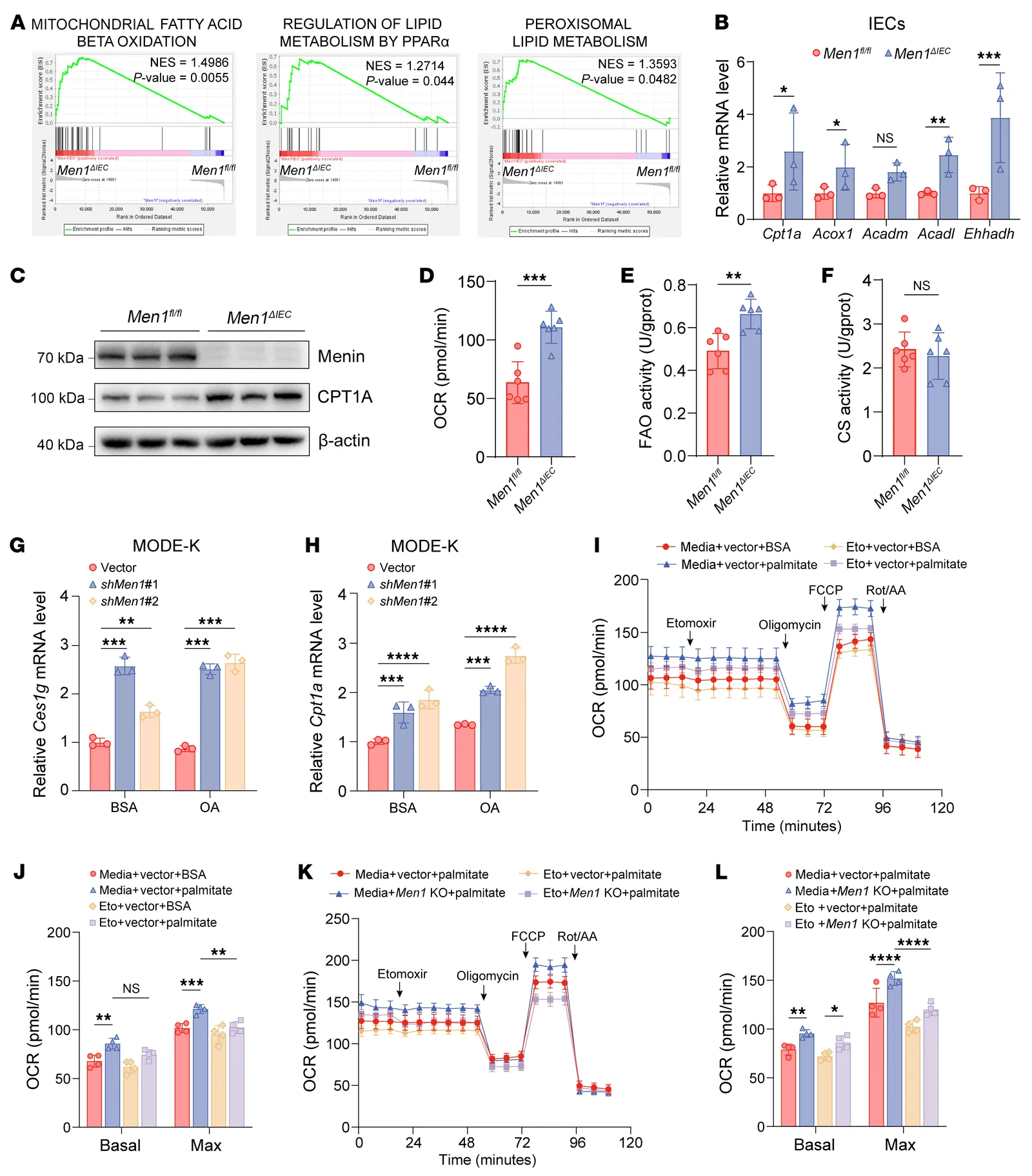
*Men1* deficiency enhances FAO capacity in IECs. (**A**) Gene set enrichment analysis (GSEA) of IEC transcriptomes from *Men1^fl/fl^* and *Men1^ΔIEC^* mice, showing enrichment of mitochondrial FAO, PPARα-regulated lipid metabolism, and peroxisomal lipid metabolism pathways. (**B**) RT-qPCR analysis of FAO-related genes in IECs isolated from *Men1^fl/fl^* and *Men1^ΔIEC^* mice. (**C**) WB analysis of menin and CPT1A protein expression in IECs from *Men1^fl/fl^* and *Men1^ΔIEC^* mice. (**D**) Oxygen consumption rate (OCR) in primary IECs. (**E**) FAO activity in primary IECs. (**F**) CS activity in primary IECs. (**G** and **H**) RT-qPCR analysis of *Ces1g* (**G**) and *Cpt1a* (**H**) mRNA levels in MODE-K cells transduced with control vector or 2 independent *Men1* shRNAs under BSA control or oleic acid treatment conditions. (**I** and **J**) Seahorse XF palmitate oxidation stress assay in control MODE-K cells treated with BSA or palmitate in the presence or absence of etomoxir (**I**), and quantification of basal and maximal OCR (**J**). FCCP, carbonyl cyanide *p*-trifluoromethoxyphenylhydrazone. (**K** and **L**) Seahorse XF palmitate oxidation stress assay in vector control and *Men1*-knockdown MODE-K cells supplied with palmitate in the presence or absence of etomoxir (**K**) and quantification of basal and maximal OCR (**L**). For **B** and **C**, *n* = 3 mice per group; for **D**–**F**, *n* = 6 mice per group; for **G**–**L**, *n* = 3. All data are presented as mean ± SD. Statistical significance was assessed by 2-way ANOVA for panels **B**, **G**, **H**, **J**, and **L** and by unpaired 2-tailed Student’s *t* test for panels **D**–**F**. *P < 0.05, **P < 0.01, ***P < 0.001, ****P < 0.0001.

**Figure 5:**
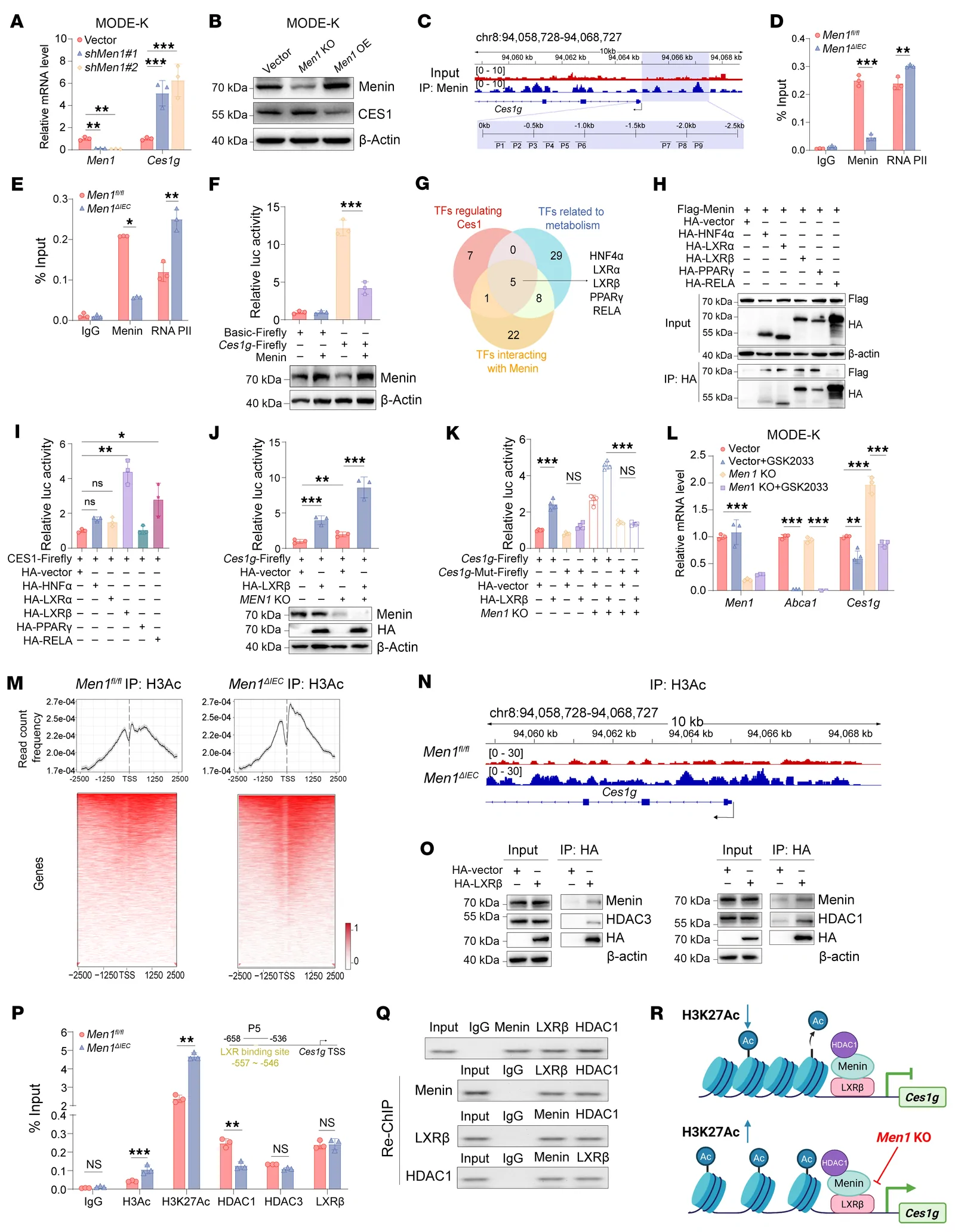
The menin–HDAC1–LXRβ complex cooperatively represses *Ces1g/CES1* transcription. (**A**) RT-qPCR of *Men1* and *Ces1g* in MODE-K cells after *shMen1* knockdown (KD). (**B**) Immunoblotting of menin and CES1 after *Men1* KD or overexpression (OE). (**C**) ChIP-seq of menin at the *Ces1g* promoter in IECs. (**D** and **E**) ChIP-qPCR of menin and polymerase II (Pol II) occupancy at *Ces1g* promoter regions P4 and P5. (**F**) *Ces1g* promoter luciferase activity with or without menin overexpression. (**G**) Overlap of transcription factors associated with CES1 regulation, metabolism, and menin interaction. (**H**) Co-IP of Flag-menin with HA-tagged HNF4α, LXRα, LXRβ, PPARγ, or RELA. (**I**) CES1 promoter activity following transcription factor OE. (**J**) *Ces1g* promoter activity in *MEN1* wild-type or knockout HEK293T cells expressing LXRβ or vector. (**K**) Activities of wild-type and double-mutant CES1 promoter reporters in control or *Men1*-KO cells expressing LXRβ or vector. (**L**) *Men1*, *Abca1*, and *Ces1g* expression in control or *Men1*-KO MODE-K cells treated with 2 μM GSK2033. (**M**) Genome-wide H3 acetylation profiles around transcription start sites (±2.5 kb) in *Men1^fl/fl^* and *Men1^ΔIEC^* IECs. (**N**) H3 acetylation tracks at the *Ces1g* locus. (**O**) Co-IP showing LXRβ association with menin, HDAC1, and HDAC3. (**P**) ChIP-qPCR analysis of H3Ac, H3K27Ac, HDAC1, HDAC3, and LXRβ occupancy at promoter region P5. (**Q**) Sequential ChIP showing co-occupancy of menin, LXRβ, and HDAC1 at the *Ces1g* promoter. (**R**) Model of LXRβ-mediated recruitment of the menin–HDAC1 complex to repress *Ces1g* transcription. For **A**, **I**, **K**, and **L**, *n* = 3; panels **B**, **F**, **H**, **J**, **O**, and **Q** show representative results from 3 independent experiments; for **D**, **E**, and **P**, *n* = 3 mice per group. All data are presented as mean ± SD. Statistical significance was assessed by 2-way ANOVA for panels **A**, **D**, **E**, **L**, and **P** and by 1-way ANOVA for panels **F** and **I**–**K**. *P < 0.05, **P < 0.01, ***P < 0.001. LXR, liver X receptor; HDAC, histone deacetylase; HNF, hepatocyte nuclear factor.

**Figure 6:**
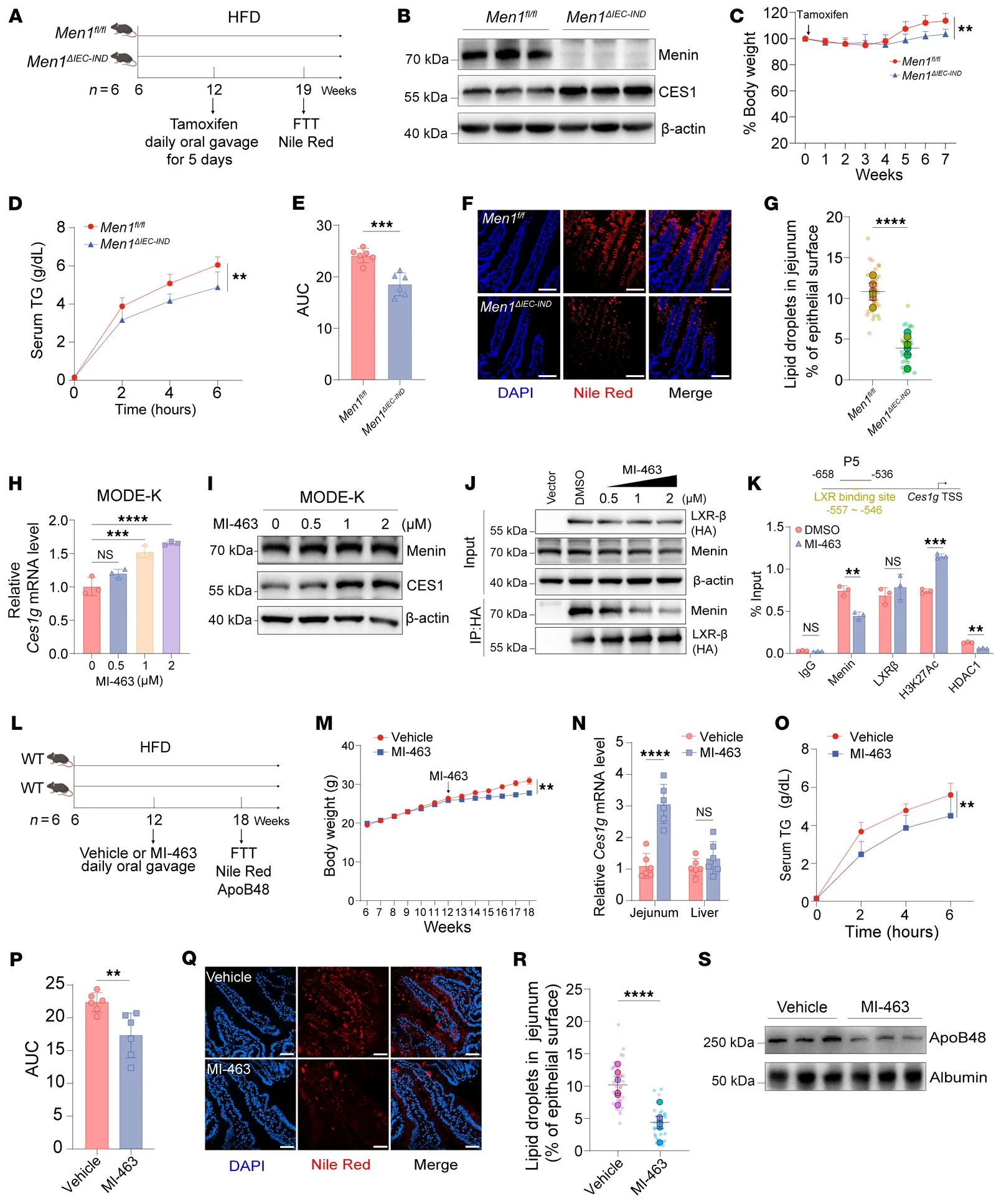
Pharmacological or genetic inhibition of intestinal menin attenuates HFD-induced weight gain in mice. (**A**) *Men1^fl/fl^* and *Men1^ΔIEC-IND^* mice were fed an HFD from 6 weeks of age, treated with tamoxifen (0.2 mg/g/d) for 5 days beginning at 12 weeks, and analyzed at 19 weeks. (**B**) Immunoblotting of menin and CES1 in IECs. (**C**) Body weight change after TAM. (**D** and **E**) Serum TG levels during the FTT and AUC. (**F** and **G**) Nile Red staining and quantification of lipid accumulation in proximal IECs. Five randomly selected fields per mouse were averaged as 1 data point. Scale bars: 50 μm. (**H** and **I**) *Ces1g* mRNA and menin/CES1 protein levels in MI-463–treated MODE-K cells. (**J**) Co-IP of Flag-menin and HA-LXRβ in MI-463–treated HEK293T cells. (**K**) ChIP-qPCR analysis of menin, LXRβ, H3K27Ac, and HDAC1 occupancy at the *Ces1g* promoter after MI-463 treatment. (**L**) HFD-fed C57BL/6 mice received vehicle or MI-463 (30 mg/kg/d, oral) from 12 to 18 weeks of age. (**M**) Body weight changes during treatment. (**N**) *Ces1g* expression in jejunum and liver. (**O** and **P**) Serum TG levels during FTT and corresponding AUC. (**Q** and **R**) Nile Red staining and quantification of intestinal epithelial lipid accumulation. Five fields per mouse were averaged as 1 data point. Scale bars: 100 μm. (**S**) Serum ApoB48 levels at 6 hours during FTT. For **A**–**G** and **L**–**R**, *n* = 6 mice per group; for **S**, *n* = 3 mice per group; for **H** and **K**, *n* = 3; panels **I** and **J** show representative results from 3 independent experiments. All data are presented as mean ± SD. Statistical analysis was assessed by 2-way ANOVA for panels **C**, **D**, **K**, and **M**–**O**; by unpaired 2-tailed Student’s *t* test for panels **E**, **G**, **P**, and **R**; and by 1-way ANOVA for panel **H**. **P < 0.01, ***P < 0.001, ****P < 0.0001.

**Figure 7:**
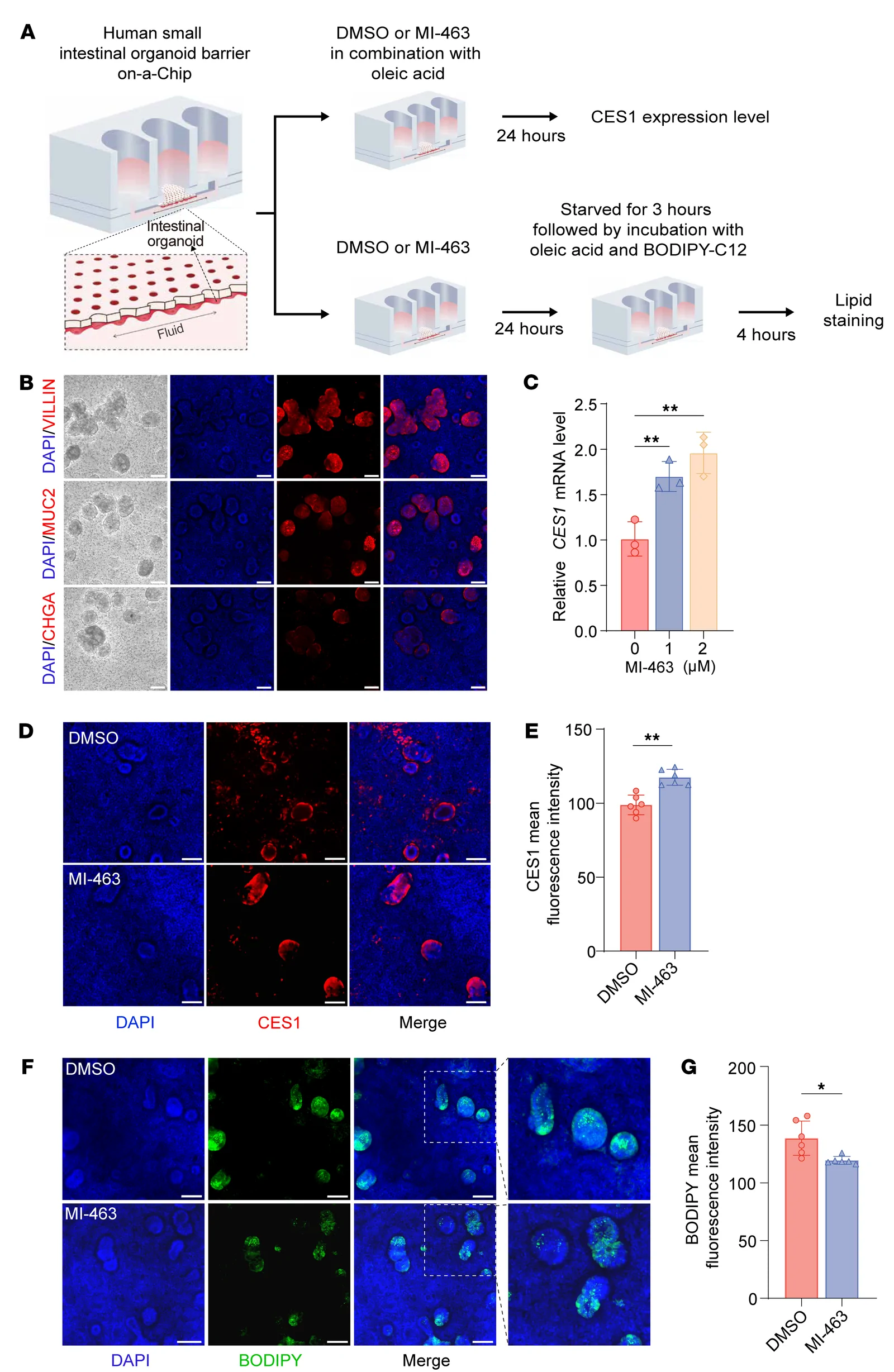
MI-463 administration enhances CES1 expression and reduces lipid accumulation in human small intestinal organoids. (**A**) Experimental design. Human small intestinal organoids were seeded in the IBAC M1 chip to establish a barrier model, treated with MI-463, and stimulated with oleic acid to mimic a high-fat condition. (**B**) Immunofluorescence staining of intestinal epithelial differentiation markers VILLIN, MUC2, and CHGA in the organoid-derived barrier model. Scale bars: 100 μm. (**C**) RT-qPCR analysis of *CES1* mRNA levels in the organoid-derived barrier. (**D** and **E**) Representative immunofluorescence staining of CES1 (**D**) and quantification of mean CES1 fluorescence intensity (**E**) in organoid-derived intestinal barriers treated with DMSO or MI-463. Six randomly selected fields per chip were analyzed, and the mean value for each chip was used as 1 data point. Scale bars: 100 μm. (**F** and **G**) Representative BODIPY-C12 staining of intracellular lipid droplets (**F**) and quantification of mean BODIPY fluorescence intensity (**G**) in organoid-derived intestinal barriers treated with DMSO or MI-463 and subsequently incubated with oleic acid. Six randomly selected fields per chip were analyzed, and the mean value for each chip was used as 1 data point. Scale bars: 100 μm. For **C**, **E**, and **G**, *n* = 3; panels **B**, **D**, and **F** show representative results from 3 independent experiments. All data are presented as mean ± SD. Statistical significance was assessed by 1-way ANOVA for panel **C** and by unpaired 2-tailed *t* test for panels **E** and **G**. *P < 0.05, **P < 0.01.

## Version 1 08/11/2026

In-Press Preview

## Version 2 10/01/2026

Electronic publication
