# Supplementary material for: Inhibiting menin attenuates high-fat diet–induced weight gain by limiting intestinal lipid absorption in mice
Source: J Clin Invest. 2026 Aug 11;136(19):e202709. doi: 10.1172/JCI202709 (PMC13626824; doi:10.1172/JCI202709)
Supplement: Supplementary file 1 [file jci-136-202709-s050.pdf]

Inhibiting menin attenuates high-fat diet-induced weight gain by limiting intestinal lipid absorption in mice

Xiaoru Cao[1,2]*, Pingping Zhou[1]*, Haiyue Meng[1], Zhitao Guo[1], Yan Cao[3], Chenghao Wang[1], Lulu Liu[1], Yinghao Guo[1], Yue Wang[1], Guoshun Xin[1], Dabin Liu[4], Feng Geng[5#], Jian Ma[1#]

1 Department of Pancreatobiliary Surgery, The Second Affiliated Hospital of Harbin Medical University and Department of Immunology, School of Basic Medical Sciences, Harbin Medical University, Harbin, 150081, China.

2 Central Laboratory, Yulin Hospital, The First Affiliated Hospital of Xi'an Jiaotong University, Yulin, 719000, China.

3 Department of Endocrinology, First Affiliated Hospital of Harbin Medical University, Harbin, 150001, China.

4 NHC Key Laboratory of Molecular Probes and Targeted Diagnosis and Therapy, The Fourth Hospital of Harbin Medical University, Harbin, 150001, China.

5 Science and Technology Research Center, Harbin Medical University Cancer Hospital; Key Laboratory of Molecular Oncology of Heilongjiang Province, Harbin, 150081, China.

[*] Xiaoru Cao and Pingping Zhou contributed equally to this work.

# Corresponding authors

Jian Ma

E-mail: jma@hrbmu.edu.cn

Tel: +86-13836031063

Postal address: No. 157, Baojian Road, Harbin, Heilongjiang, 150081, China.

Feng Geng

E-mail: gengfeng@hrbmu.edu.cn

Tel: +86-15145141075

Postal address: No. 150, Haping Road, Harbin, Heilongjiang, 150081, China.

Supplemental Information

1. **Supplemental Methods**
2. **Supplemental Figures and Figure legends 1-7**
3. **Supplemental Tables 1-7**

1. Supplemental Methods

Cell culture

The cell lines used in this study included HEK293T, CACO-2, MODE-K, which were obtained from the Cell Bank of the Chinese Academy of Sciences (Shanghai, China). All cells were maintained in Dulbecco's Modified Eagle Medium (Gibco, Thermo Fisher Scientific) or RPMI-1640 supplemented with 10% fetal bovine serum (Gibco, Thermo Fisher Scientific), 100 U/mL penicillin, and 100 μg/mL streptomycin, at 37°C in a humidified atmosphere containing 5% CO_2. All cell lines were authenticated and validated prior to experiments and were routinely tested for mycoplasma contamination, with negative results.

Immunohistochemistry (IHC)

Tissues were fixed in 4% paraformaldehyde, dehydrated, cleared, embedded in paraffin, and sectioned. After routine deparaffinization and rehydration, antigen retrieval was performed using microwave heating in sodium citrate buffer (pH 6.0). Sections were cooled to room temperature and treated with 3% hydrogen peroxide for 10 minutes to block endogenous peroxidase activity. After washing with PBS three times, sections were blocked at room temperature for 1 hour and incubated with primary antibodies at 4°C overnight. The following day, after PBS washing, sections were incubated with secondary antibodies at room temperature for 1 hour, followed by additional washes. Hematoxylin was used for nuclear counterstaining and bluing. Finally, sections were

cleared with xylene, mounted with neutral resin, and observed under a light microscope for imaging and analysis.

Primary intestinal epithelial cell isolation

The proximal jejunum (approximately 15 cm distal to the stomach) was excised and rinsed with PBS. The intestine was opened longitudinally, washed thoroughly, and cut into ~0.5 cm segments. These were incubated in digestion buffer (1.5 mmol/L EDTA and 0.5 mmol/L DTT) at 37°C with shaking at 150 rpm for 20 minutes. Undigested tissue was removed, and the cell suspension was centrifuged at 4°C, 500×*g* for 5 minutes. The resulting pellet contained intestinal epithelial cells (1). Then cells were stained with antibodies against CD45, together with a viability dye. IECs were defined as live $CD45^-$ cells. Flow cytometric analysis was performed using a flow cytometer, and data were analyzed with FlowJo software.

FITC-Dextran intestinal permeability assay

Following 12 hours of fasting, mice were gavaged with FITC-dextran (FD4-250MG, Sigma-Aldrich) at a dose of 800 mg/kg (dissolved in 200 µL PBS). Blood samples were collected 4 hours later. Serum fluorescence intensity was measured at 485/530 nm.

Nile Red staining

Primary intestinal epithelial cells were seeded and cultured for 24 hours, followed by treatment with medium containing BSA and 400 µMol/L oleic acid for another 24 hours. Frozen sections (5 µM) were also prepared from intestinal tissues collected after the FTT. Cells and tissue sections were stained identically. A Nile Red stock solution (1 mg/mL in DMSO) was diluted to a working concentration of 1 µg/mL. After washing

with PBS or culture medium, samples were incubated with Nile Red working solution in the dark for 15 minutes, followed by three gentle PBS washes. DAPI was then added for nuclear staining (5 minutes, in the dark), followed by three additional washes. Staining was visualized under a fluorescence microscope using a 540 nm excitation wavelength. Lipid droplet-positive areas were analyzed using ImageJ.

Intraperitoneal glucose tolerance tests (IPGTTs) and intraperitoneal insulin tolerance tests (IPITTs)

After 12 weeks of HFD feeding, mice were subjected to IPGTTs and IPITTs. For the IPGTT, mice were fasted for 16 hours prior to the test, followed by an intraperitoneal injection of glucose at a dose of 2 g/kg body weight. Blood glucose levels were then monitored at 0, 15, 30, 60, and 120 minutes post-injection using a handheld glucometer (Roche) with samples collected from the tail vein. For the IPITTs, mice underwent a 6-hour fasting period the day before the test. Insulin was administered intraperitoneally at a concentration of 1 U/kg, and blood glucose was similarly measured at 0, 15, 30, 60, and 120 minutes post-injection.

Fat tolerance tests (FTTs)

After 10 hours of fasting, mice were injected with Tyloxapol (T0307, Sigma-Aldrich, 500 mg/kg). Fifteen minutes later, olive oil (10 μL/g) was administered via oral gavage. Blood samples were collected at 0, 2, 4, and 6 hours post-gavage. Serum was obtained by centrifugation at 3000 rpm for 10 minutes. Serum TG levels were measured using a commercial kit (A110-1-1, Nanjing Jiancheng Bioengineering Institute). For hepatic VLDL output analysis, mice were fasted for 10–12 h and injected with tyloxapol at 500

mg/kg body weight to inhibit peripheral TG clearance. Blood samples were collected at 0, 1, 2, and 3 h after injection. Plasma VLDL levels were measured using a commercial mouse VLDL assay kit (H249-1-2 96t, Nanjing Jiancheng Bioengineering Institute) according to the manufacturer's protocol.

Transmission electron microscopy (TEM)

After 10 hours of fasting, male mice were gavaged with corn oil (10 μL/g). Six hours later, jejunal tissues were fixed in 2.5% glutaraldehyde. After dehydration, embedding, and ultrathin sectioning, samples were negatively stained and observed using a Hitachi HT7800 transmission electron microscope.

Metabolic cage monitoring

Energy metabolism was assessed using a comprehensive lab animal monitoring system (CLAMS; Oxymax-CLAMS, Columbus Instruments). Male mice fed a high-fat diet for 12 weeks were individually housed in metabolic cages 7 days under controlled conditions (22°C, 12 h light/12 h dark cycle) with ad libitum access to food and water. After this 7-day acclimation period, oxygen consumption (VO_2), carbon dioxide production (VCO_2), and locomotor activity were continuously recorded. Energy expenditure (EE) was calculated based on VO_2 and VCO_2 and normalized to lean body mass. For analysis, data collected between 24 and 60 h after the start of monitoring were used. EE was plotted as a function of time, with the dark phase indicated by shaded regions. In addition, the average EE, normalized to lean mass was calculated and compared across three time periods: the entire 24–60 h interval, the dark phase, and the light phase.

Fecal lipid measurement

Lipid extraction from jejunal tissue was performed using a modified Folch method (2). Tissue homogenates were mixed with chloroform: methanol (2:1), centrifuged for phase separation, and the lower lipid phase was collected, dried, and re-dissolved in water for TG measurement. For fecal lipid extraction, feces were collected after 24 hours of individual housing, suspended in saline, then extracted with chloroform/methanol. Lipid weight and TG content were subsequently measured.

Carboxylesterase (CarE) activity assay

Approximately 0.1 g of intestinal tissue was homogenized in 1 mL extraction buffer on ice, then centrifuged at 15,000 rpm and 4°C for 10 minutes. The supernatant was used for detection. A CarE activity assay kit (BC0845, Solarbio) was used according to the manufacturer's instructions. Reagent I was pre-warmed at 37°C; reagent II was kept on ice. For the assay, 10 μL supernatant, 120 μL reagent II, and 70 μL reagent I were added to a 96-well plate and mixed. Absorbance was measured at 10 and 310 seconds to calculate ΔA. CarE activity was calculated in U/g tissue using the provided formula.

RNA-sequencing

Male *Men1*$^{f/f}$ and intestinal epithelium-specific *Men1* knockout mice were maintained on a high-fat diet for 4 weeks. Upon completion of dietary intervention, primary intestinal epithelial cells were isolated from the small intestinal tissues of mice from both genotypes. Total RNA was extracted from isolated primary epithelial cells, and residual genomic DNA was thoroughly digested and eliminated. After quality verification, strand-specific RNA-seq libraries were constructed via end repair, adapter

ligation and PCR amplification steps, and paired-end sequencing was subsequently performed on the Illumina NovaSeq 6000 platform. Raw sequencing reads were deposited in the NCBI Sequence Read Archive (SRA) under accession number PRJNA1502564. For bioinformatic analysis, FastQC was applied to assess raw read quality; qualified clean reads were aligned to the mouse reference genome using HISAT2. Transcript abundance was quantified with RSEM and featureCounts, and differentially expressed genes were screened using DESeq2. Functional enrichment analyses including GO annotation and GSEA were further conducted to interpret biological functions of altered transcripts.

Western blotting (WB)

Tissues were lysed in RIPA buffer (Solarbio) with protease inhibitors (Roche). Protein concentrations were measured using a BCA kit (Solarbio). Samples were separated by SDS-PAGE and transferred to PVDF membranes (Millipore). After incubation with primary antibodies (see Supplementary Table 7) and HRP-conjugated secondary antibodies, signals were detected using a chemiluminescence system (ChemiScope 6200Touch). Densitometric analysis was performed using ImageJ, normalized to ACTIN or ALBUMIN. Chylomicron samples were diluted 1:5 to avoid signal saturation for ALB and APOB48 detection.

Co-immunoprecipitation (Co-IP)

293T cells were collected after 36 h transfection in 6 cm dishes, washed with cold PBS, and lysed in 800 μL RIPA buffer on ice for 30 minutes. Lysates were centrifuged at 12,000 x *g* for 15 minutes at 4°C. Supernatants were quantified by BCA, and 40 μL was

saved as input. Equal amounts of protein were incubated overnight at 4°C with antibody-conjugated magnetic beads. Complexes were washed 6–8 times with PBST and resuspended in 1× SDS loading buffer. Samples were denatured at 100°C for 10 minutes before WB analysis.

ELISA

Serum insulin, triglyceride, cholesterol, and free fatty acids levels in mouse serum were measured using commercial ELISA kits (E-EL-M1382, Elabscience; A110-1-1 (triglyceride) and A111-1-1 (cholesterol) from Nanjing Jiancheng Bioengineering Institute, BC0595, Solarbio) according to the manufacturer's protocol. Absorbance was measured using a SpectraMax M5 from Molecular Devices, and analyte levels were calculated from standard curves or kit-provided formulas.

RT-qPCR

Total RNA was extracted with TRIzol (15596018, Invitrogen). Reverse transcription was performed using HiFi Script gDNA Removal RT Kit (CW2020M, CWBio). qPCR was performed using TransStart Top Green qPCR SuperMix (AQ131, Transgene) on a 7500 system (Applied Biosystems). Relative expression was calculated using the 2^-ΔΔCt method with β-actin as the internal control. Primers were synthesized by Kumei Biotech (see Supplementary Table 2).

Transepithelial electrical resistance (TEER) measurement

CACO-2 cells were cultured on transwell inserts for 21 days to allow polarization and tight junction formation. TEER was measured using an ERS-2 voltohmmeter (Millicell)

by placing electrodes in the upper (0.5 mL) and lower (1.5 mL) chambers. Readings were multiplied by the membrane surface area (1.12 cm^2) and expressed in $\Omega \cdot cm^2$.

Alkaline phosphatase (AKP) activity assay

After 18 days of CACO-2 culture in transwell dishes, the medium was refreshed and cultured for 3 more days. Supernatants were collected by centrifugation at 1,000–1,500 rpm for 10 minutes. AKP activity was measured using a commercial kit (A059-2-2, Nanjing Jiancheng) according to the manufacturer's protocol.

Apparent permeability coefficient (Papp) assay

A standard curve was established using sodium fluorescein at concentrations from 0 to 5 μg/mL in black 96-well plates. CACO-2 cells cultured in transwell dishes for 21 days were incubated with 20 μg/mL sodium fluorescein in the apical chamber. Samples were collected from the basolateral side at 30 and 60 minutes. Papp was calculated using: $Papp = (dQ/dt) / (A \times C_0)$, where dQ/dt is the amount transported per time, A is membrane area (1.12 cm^2), and C_0 is the initial donor concentration (20 μg/mL).

BODIPY-C12 fatty acid transport assay

CACO-2 cells were cultured on transwell dishes for 21 days. The apical chamber was treated with a lipid micelle mixture (0.6 mM oleic acid, 2 mM sodium taurocholate, 0.2 mM monoolein, 0.05 mM cholesterol, 0.2 mM lysophosphatidylcholine), with or without 5 μM BODIPY-oleic acid. After 10 minutes of incubation, media were replaced with serum-free DMEM. After 4 hours, 50 μL of basolateral medium was collected and fluorescence (Ex 480 nm / Em 510 nm) was measured.

Generation of Knockdown and Knockout Cell Lines

shRNA targeting *Men1* was cloned into pLKO.1 (primer sequences from Sigma-Aldrich), and sgRNAs for *Ces1g* and *CES1* were designed (see Synthego, Supplementary Table 4). Constructs were sequence-verified and used for lentiviral packaging with psPAX2-Gag-pol and pMD2G-VSV-G via calcium phosphate transfection. After 48 hours, viral supernatant was collected and used to infect MODE-K or CACO2 cells in the presence of polybrene (10 μg/mL). After 48 hours, cells were selected with puromycin and validated by WB.

Dual-Luciferase reporter assay

Promoter regions (~−2000 to +500 bp) of mouse *Ces1g* and human *CES1* were PCR-amplified and cloned into a luciferase reporter vector. Primers were synthesized by Kumei Biotech (see Supplementary Table 3). Constructs were verified by sequencing. Transfection into MODE-K or CACO2 cells was done at 70–80% confluence using PEI (1:5 DNA: PEI). After 36 hours, luciferase activity was measured using the Dual-Luciferase Reporter Assay Kit (E1960, Promega). Firefly and Renilla signals were read sequentially to assess promoter activity.

Plasmid construction

PCR was performed using primers (Supplementary Table 5) to amplify transcription factors (HNF4α, LXRα, LXRβ, PPARγ and RELA). Products were gel-purified, and the pcDNA3.1 vector was linearized with *Eco*RI and *Hind*III. Fragments were ligated into the vector, transformed into competent cells, and verified by sequencing. Validated plasmids were used for PEI transfection into 293T cells.

ChIP-qPCR

Primary intestinal epithelial cells or CACO2 cells were crosslinked with 1% formaldehyde for 10–15 min, quenched with glycine, lysed, and chromatin was sheared into 150–500 bp fragments by sonication or enzymatic digestion. Chromatin was incubated with antibodies (to menin, HDAC1, HDAC3) and protein A/G magnetic beads overnight. After washing, crosslinks were reversed, and DNA was purified. qPCR was performed to detect enrichment at promoters of *Ces1g/CES1*. Relative binding was calculated as %Input. Primer sequences are listed in Supplementary Table 6.

ChIP-seq

Chromatin immunoprecipitation (ChIP) DNA libraries derived from primary intestinal epithelial cells were sequenced using the BGISEQ-500 platform. Sequencing reads were aligned to the mouse reference genome (mm39) using SOAP aligner (v2.21t). Following alignment, reads were converted, sorted, and duplicate reads were removed using the rmdup function from the SAMtools suite. To pinpoint significant menin-binding regions, peak calling was performed with MACS2 by comparing ChIP samples to corresponding input controls. Only peaks with a statistical significance of $P < 10^{-5}$ were retained for downstream analysis. Peak annotation to nearby genes was carried out using the annotatePeaks.pl script in the HOMER package. Visualization of peak distribution and morphology was performed using the Integrative Genomics Viewer (IGV). Sequencing was completed by BGI Tech (Shenzhen, China), and raw ChIP-seq sequencing data generated in this study have been deposited in the NCBI database under BioProject accession PRJNA1313781 and will be released to public after August 1, 2026. A reviewer access link is available at

https://dataview.ncbi.nlm.nih.gov/object/PRJNA1313781?reviewer=i880e8onm9i2g3qasb9kh9i6q8.

Re-ChIP

According to the study by Feng *et al.* (3), cells were first cross-linked with 1% formaldehyde at room temperature for 10 minutes. The cross-linking was then quenched by adding 125 mmol/L glycine, followed by incubation on ice for 5 minutes. After washing the cells twice with PBS, they were collected and lysed in lysis buffer on ice for 10 minutes. Chromatin was then sheared to 200–500 bp fragments by sonication. After removing cell debris, an equal amount of chromatin (approximately 5−10 μg) was used for pre-clearing. In the first round of ChIP, chromatin was incubated with 2–3 μg of a specific antibody against the target protein overnight at 4°C with rotation. Then, Protein A/G magnetic beads were added and incubated for 4 hours. The beads were washed five times with low-salt and high-salt buffers sequentially to remove nonspecific bindings. DNA was purified using a PCR Purification Kit. To elute the first immunoprecipitated complex, 20 mmol/L DTT was added and incubated at 37°C for 30 minutes. The eluted product was then diluted 1:50 in ChIP dilution buffer (containing 1% Triton X-100, 2 mmol/L EDTA, 150 mmol/L NaCl, and 20 mmol/L Tris-HCl, pH 8.1). A second antibody against another target protein was added for the second round of immunoprecipitation and incubated overnight at 4°C with rotation. Protein A/G magnetic beads were added again and incubated for 4 hours, followed by buffer washes. Finally, immune complexes were eluted with 1% SDS in Tris-EDTA buffer (pH 8.0), and DNA was purified for PCR amplification of specific gene promoter

regions. PCR products were analyzed by agarose gel electrophoresis.

Supplemental references

1. Rubbino F. GPR120 prevents colorectal adenocarcinoma progression by sustaining the mucosal barrier integrity. *Sci Rep.* 2022;12:381.
2. Kraus D. Lipid extraction from mouse feces. *Bio Protoc.* 2015;5:e1375.
3. Feng D. CARM1 drives triple-negative breast cancer progression by coordinating with HIF1A. *Protein Cell.* 2024;15:744-65.

2. Supplemental Figures

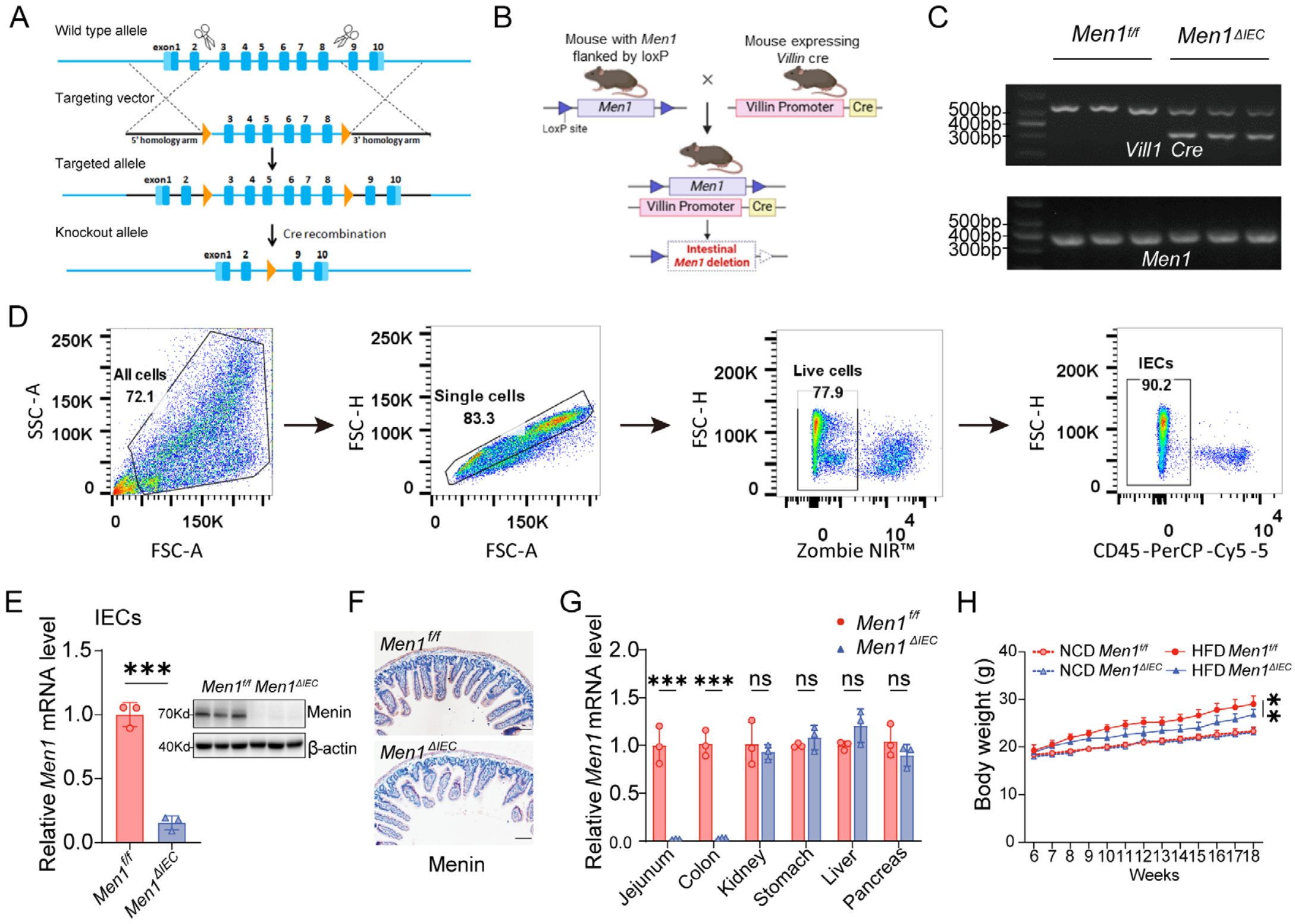

Supplemental Figure 1. IEC-specific deletion of *Men1* attenuates HFD-induced weight gain in mice.

(A) Schematic representation of the generation strategy for conditional *Men1* knockout mice. Floxed alleles flanking exons 3 and 8 of the *Men1* gene were introduced via CRISPR/Cas9-mediated homologous recombination and crossed with Cre-expressing mice to generate conditional knockouts. (B) Strategy for the generation of *Men1*$^{\Delta IEC}$ mice. (C) Genotyping results using PCR on tail DNA showing amplification of *Villin*-Cre and *Men1* alleles. (D) Flow cytometric gating strategy for isolation and enrichment of IECs. (E) RT-qPCR and WB analysis of *Men1* mRNA levels and protein expression in IECs of *Men1*$^{f/f}$ and *Men1*$^{\Delta IEC}$ mice. (F) Immunohistochemical staining showing menin protein localization and expression in proximal small intestine of 12-week HFD-fed *Men1*$^{f/f}$ and *Men1*$^{\Delta IEC}$ mice. (G) RT-qPCR analysis of *Men1* mRNA expression across various tissues in *Men1*$^{f/f}$ and *Men1*$^{\Delta IEC}$ mice. (H) Body weight curves of female *Men1*$^{f/f}$ and *Men1*$^{\Delta IEC}$ mice under NCD or HFD feeding. For C, E, and G, n = 3 mice

per group. All data are presented as mean ± SD. Statistical significance was determined by two-way ANOVA for panels G and H; and by unpaired Student's *t*-test for panel E. $^{**}P < 0.01$, $^{***}P < 0.001$; ns, not significant.

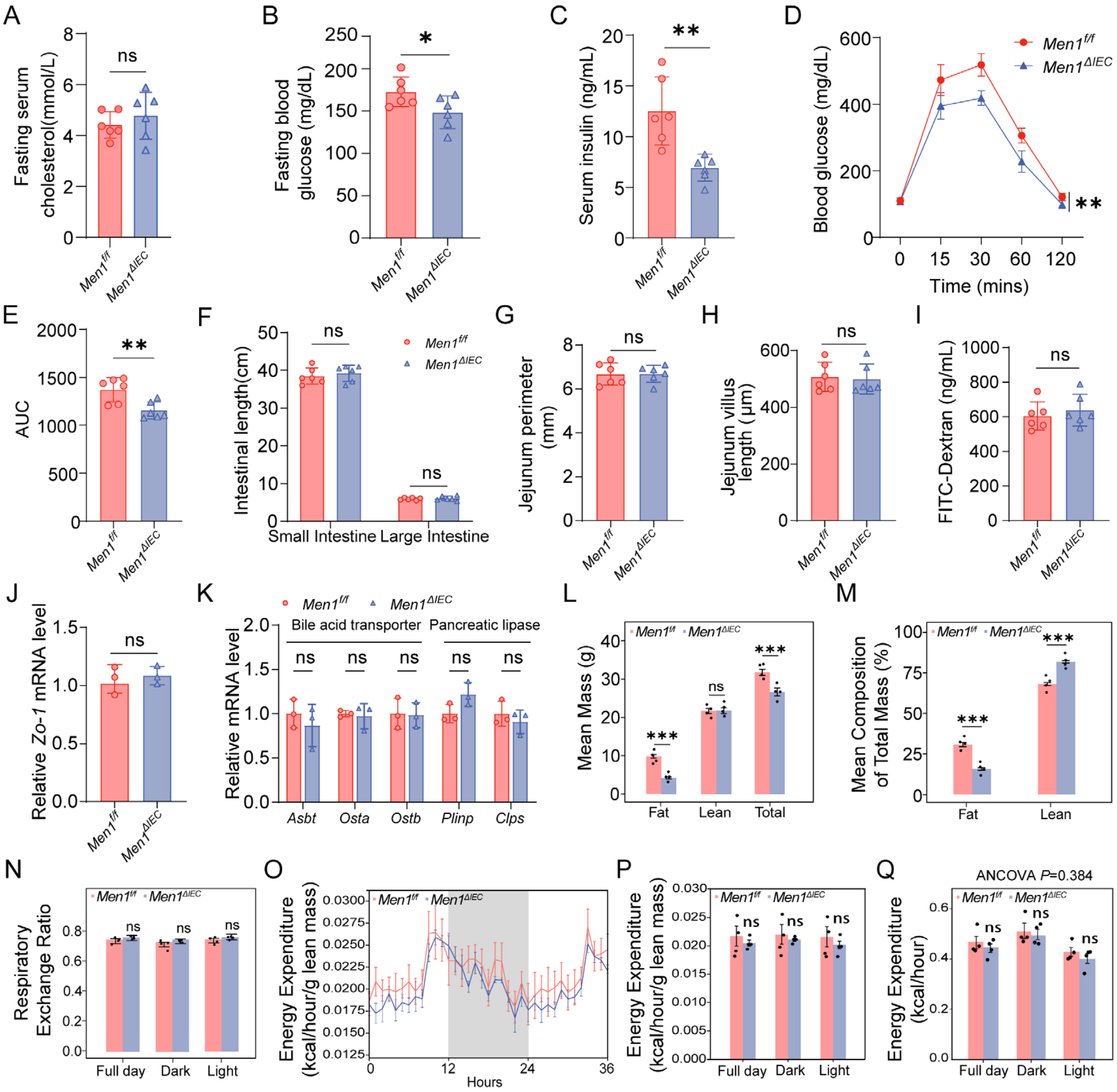

Supplemental Figure 2. IEC-specific deletion of *Men1* does not alter energy expenditure in mice.

(A–C) Fasting serum cholesterol, blood glucose, and serum insulin; insulin was measured after 10 weeks of HFD. (D) IPGTT after 10 weeks of HFD, with blood glucose measured at 0, 15, 30, 60, and 120 min after injection. (E) IPGTT area under the curve. (F) Lengths of the small and large intestines in *Men1*$^{f/f}$ and *Men1*$^{\Delta IEC}$ mice. (G and H) Jejunal circumference and villus length. (I) Serum FITC-dextran levels 4 h after gavage following a 12-h fast. (J) RT-qPCR analysis of *Zo-1* mRNA in primary small intestinal cells. (K) RT-qPCR analysis of ileal bile acid transporter genes (*Asbt*, *Osta*, and *Ostb*) and pancreatic lipase-related genes (*Pnlip* and *Clps*). (L and M) Fat mass, lean mass, body weight, and percentages of body fat and lean mass measured by

CLAMS. (N) Respiratory exchange ratio, calculated as CO_2 production/O_2 consumption, monitored continuously for 36 h by CLAMS. (O) Energy expenditure normalized to lean body mass, monitored for 36 h; gray shading indicates the dark phase. (P) Mean normalized energy expenditure during the full day, dark phase, and light phase. (Q) Energy expenditure during these periods adjusted for lean body mass by ANCOVA. Unless otherwise indicated, analyses were performed after 12 weeks of HFD feeding. For A–I, $n = 6$ mice per group; for J and K, $n = 3$ mice per group; for L–Q, $n = 4$ mice per group. All data are presented as mean ± SD. Statistical significance was assessed by two-way ANOVA for panels D, F, K–N, and P; by unpaired Student's *t*-test for panels A–C, and E–J; and by ANCOVA for panel Q. *$P < 0.05$, **$P < 0.01$, ***$P < 0.001$; ns, not significant.

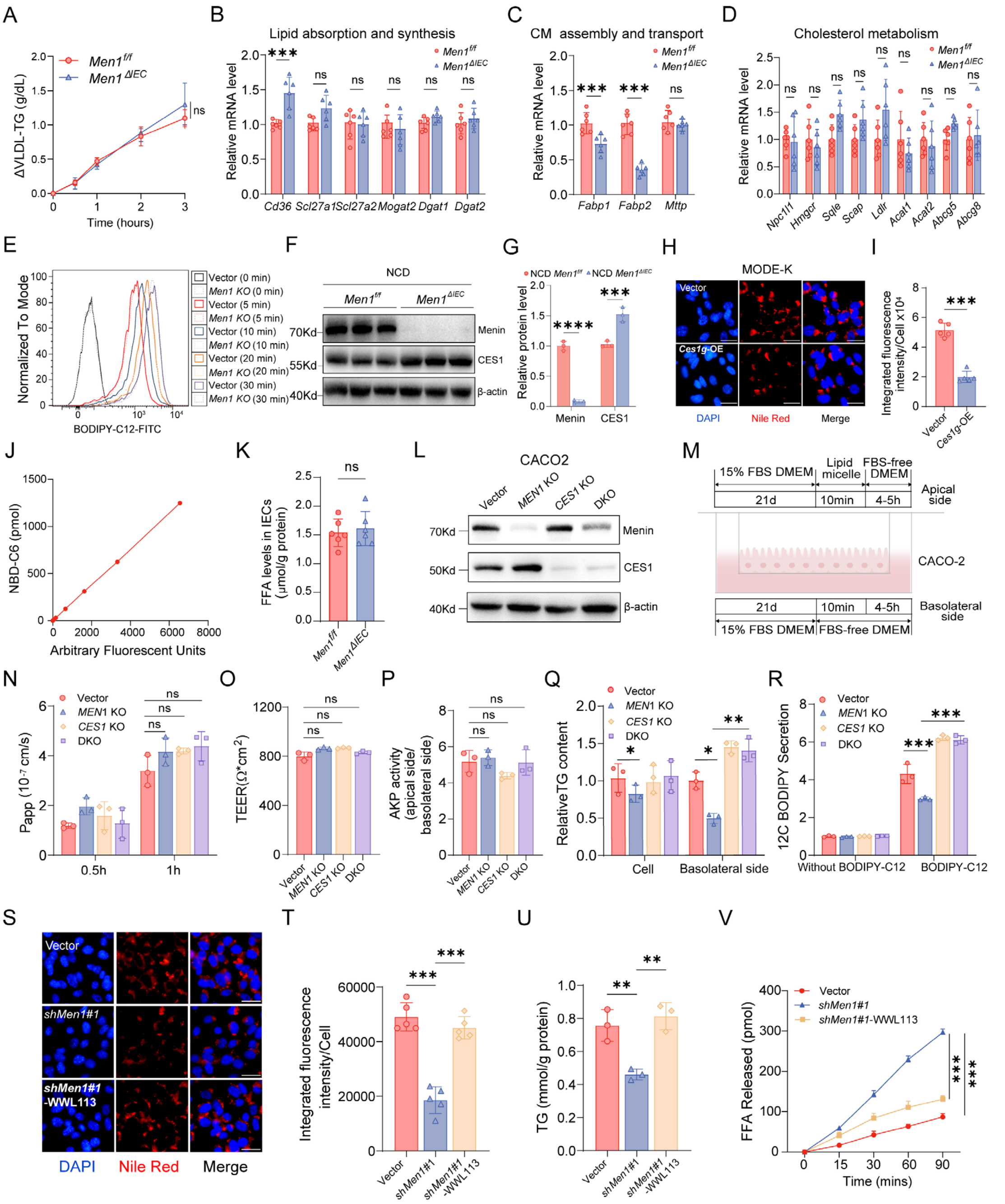

Supplemental Figure 3. ***Men1*** **deficiency promotes** ***Ces1*****-mediated lipid catabolism.** (A) Tyloxapol-induced TG accumulation, indicating hepatic VLDL-TG output, in *Men1*$^{f/f}$ and *Men1*^{ΔIEC} mice at 0–3 h after injection. (B-D) RT-qPCR analysis of genes involved in lipid absorption and re-esterification (B), lipid transport (C), and cholesterol metabolism (D) in jejunal epithelial cells after 4 weeks of HFD. (E) BODIPY 500/510 C1, C12 uptake in control and *Men1*-knockdown MODE-K cells over 30 min. (F and G) Immunoblotting and quantification of menin and CES1 in jejunal epithelial cells

after 12 weeks of NCD. (H and I) Nile Red staining and quantification in *Ces1g*-overexpressing or control MODE-K cells treated with 400 μM oleic acid for 24 h. (J) NBD-C6 standard curve for the NBD-TAG lipid hydrolysis assay. (K) Free fatty acid levels in primary IECs from *Men1*$^{f/f}$ and *Men1*$^{\Delta IEC}$ mice. (L) Immunoblotting of menin and CES1 in Vector, *MEN1*-KO, *CES1*-KO, and DKO CACO-2 cells. (M) Transwell lipid-transport assay in CACO-2 monolayers differentiated for 21 days, exposed apically to lipid micelles for 10 min, and incubated in serum-free DMEM for 4–5 h before cellular and basolateral TG measurement. (N-P) Sodium fluorescein permeability at 0.5 and 1 h (N), transepithelial electrical resistance (O), and apical-to-basolateral alkaline phosphatase activity ratio (P). (Q) Intracellular and basolateral TG after lipid uptake in Vector, *MEN1*-KO, *CES1*-KO, and DKO CACO-2 cells. (R) Basolateral BODIPY-lipid fluorescence with or without BODIPY-C12. (S and T) Nile Red staining and quantification in Vector, *Men1*-knockdown, and WWL113-treated *Men1*-knockdown MODE-K cells after oleic acid treatment. (U) Intracellular TG under the same conditions. (V) FFA release measured by the NBD-TAG assay. For A–D, K, $n = 6$ mice per group; for F and G, $n = 3$ mice per group; for E, J, L and N-R, U and V, $n = 3$; for I and T, $n = 5$; panels H and S show representative results from five independent experiments. All data are presented as mean ± SD. Statistical significance was assessed by two-way ANOVA for panels A–D, G, N, Q–R, and V; by unpaired Student's *t*-test for panels I and K; and by one-way ANOVA for panels O-P, and T–U. *$P < 0.05$, **$P< 0.01$, ***$P < 0.001$; ns, not significant.

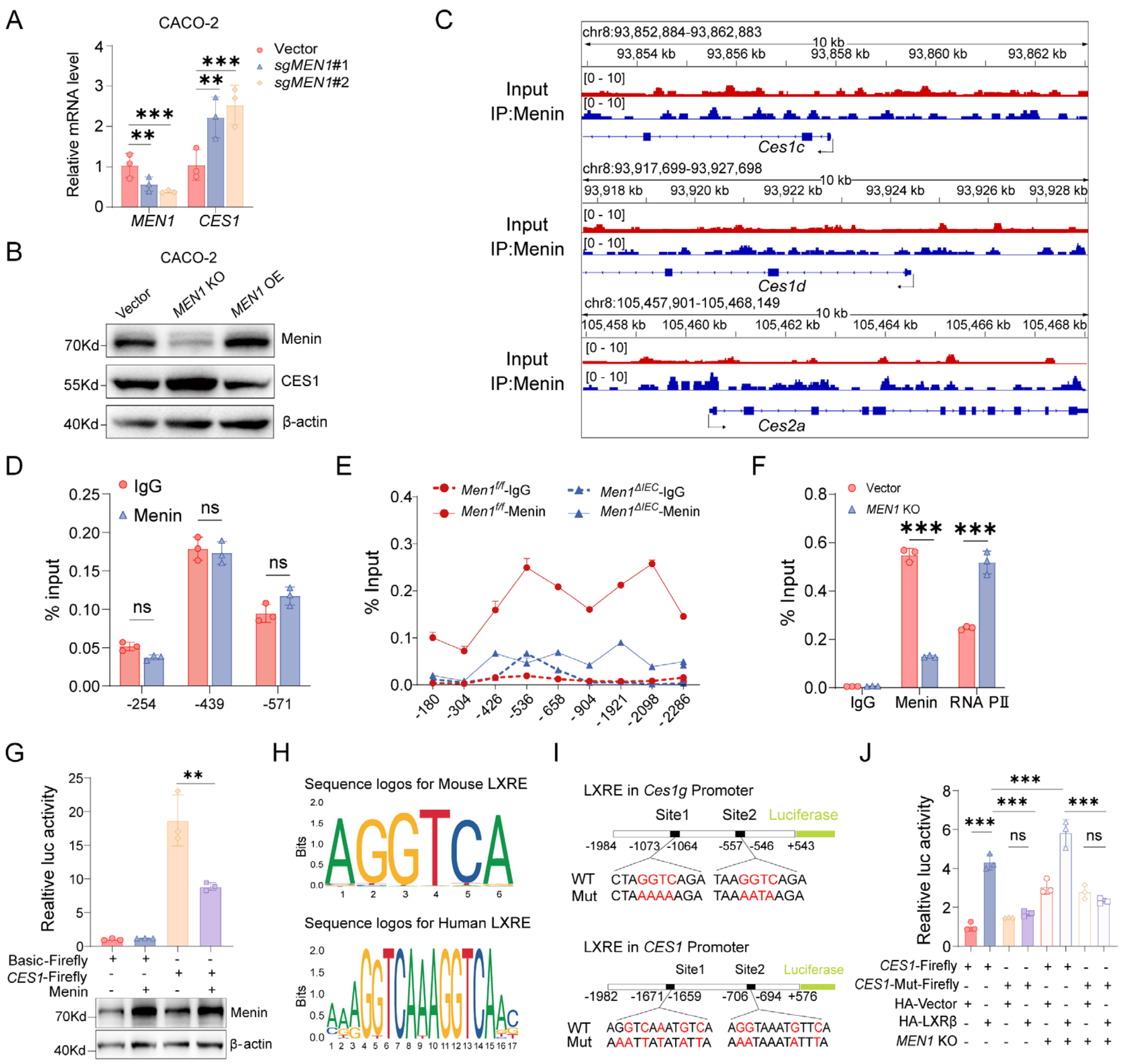

Supplemental Figure 4. The menin-HDAC1-LXRβ complex cooperatively represses *Ces1g/CES1* transcription.

(A) RT-qPCR detection of *MEN1* and *CES1* mRNA levels in CACO-2 cells following CRISPR-Cas9-mediated knockout using *sgMEN1*#1 and *sgMEN1*#2. (B) WB analysis of menin and CES1 protein expression in *Men1*-knockout or overexpressing CACO-2 cells. (C) ChIP-Seq analysis showing menin binding at the promoter regions of *Ces1c*, *Ces1d*, and *Ces2a* in mouse small intestinal epithelial cells. Gene annotation tracks are shown below. (D) ChIP-qPCR analysis of menin enrichment at three candidate regions of the *Ces2a* promoter. IgG was used as a negative control. (E) ChIP-qPCR validation of menin enrichment at 9 regions of the *Ces1g* promoter in *Men1*$^{f/f}$ and *Men1*$^{\Delta IEC}$ mice. (F) ChIP-qPCR analysis of menin and RNA Polymerase II binding to the *CES1* promoter in CACO-2 cells under vector or *MEN1* knockout conditions. (G) Dual-

luciferase reporter assay assessing transcriptional activity of *CES1* promoter in *MEN1* wild-type or knockout HEK293T cells. (H) Sequence logos representing predicted LXR response elements (LXREs) in the mouse *Ces1* (top) and human *CES1* (bottom) promoters. (I) Predicted LXRE sites (site 1 and site 2) within mouse *Ces1g* and human *CES1* promoters, with nucleotide sequences and mutated bases marked in red. (J) WT or mutant *CES1* promoter luciferase constructs were co-transfected with Renilla control into *MEN1* wild-type or knockdown HEK293T cells, followed by HA-vector or HA-LXRβ expression and dual-luciferase assay. For D and E, $n = 3$ mice per group; for A, F, G and J, $n = 3$; panels B and G show representative results from three independent experiments. All data are presented as mean ± SD. Statistical significance was assessed by two-way ANOVA for panels A, D, and F; and by one-way ANOVA for panels G and J. $^{**}P < 0.01$, $^{***}P < 0.001$; ns, not significant.

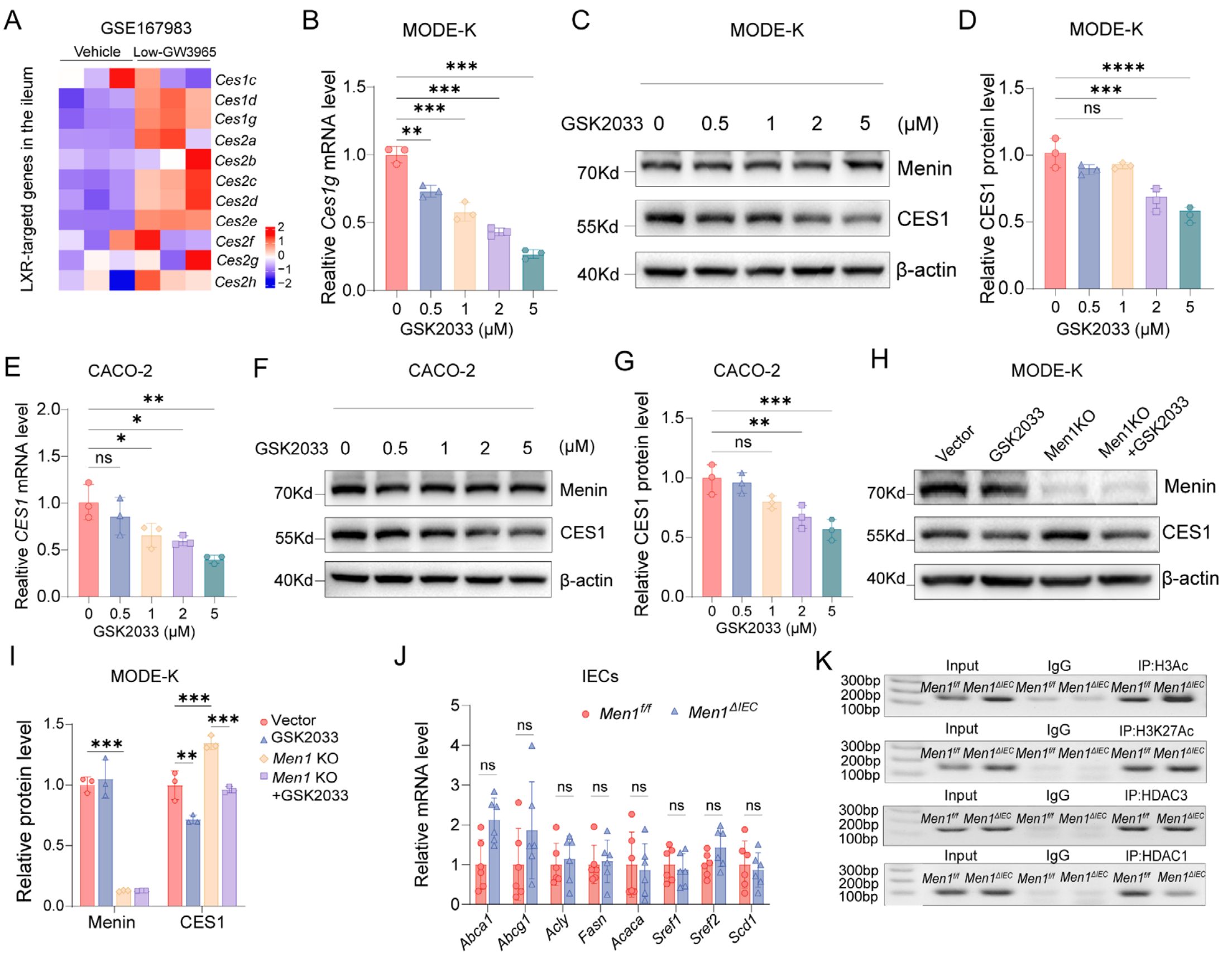

Supplemental Figure 5. LXR inhibition suppresses *Ces1g/CES1* expression and reverses its upregulation induced by *Men1* deficiency.

(A) Differential expression analysis of carboxylesterase family genes based on RNA-seq dataset GSE167983, showing selective induction in the intestine following low-dose LXR agonist GW3965 treatment. (B) RT-qPCR and analysis of *Ces1g* mRNA levels in MODE-K cells treated with increasing doses of the LXR antagonist GSK2033 for 24 h. (C and D) WB and densitometric quantification of menin and CES1 protein expression in MODE-K cells treated with varying concentrations of GSK2033. (E-G) RT-qPCR and WB analysis of *CES1* mRNA and CES1 protein expression in CACO-2 cells treated with increasing concentrations of GSK2033 for 24 h, followed by densitometry of protein bands. (H and I) WB and densitometry of menin and CES1 expression in MODE-K cells treated with LXR antagonist GSK2033 under *Men1* knockdown or control conditions. (J) RT-qPCR analysis of representative canonical LXR-responsive genes involved in cholesterol efflux and lipid metabolism in IECs

isolated from *Men1*$^{f/f}$ and *Men1*$^{\Delta IEC}$ mice. (K) ChIP-PCR analysis of acetylation marks (H3Ac, H3K27Ac) and histone deacetylases HDAC1 and HDAC3 at the *Ces1g* promoter region in intestinal epithelial cells from *Men1*$^{f/f}$ and *Men1*$^{\Delta IEC}$ mice. Target regions were amplified after chromatin immunoprecipitation with the indicated antibodies. For B, D, E, G, and I, $n = 3$; panels C, F and H show representative results from three independent experiments; for J, $n = 6$ mice per group. All data are presented as mean ± SD. Statistical significance was assessed by two-way ANOVA for panels I-J; and by one-way ANOVA for panels B, D, E, and G. *$P < 0.05$, **$P< 0.01$, ***$P < 0.001$ ****$P < 0.0001$; ns, not significant.

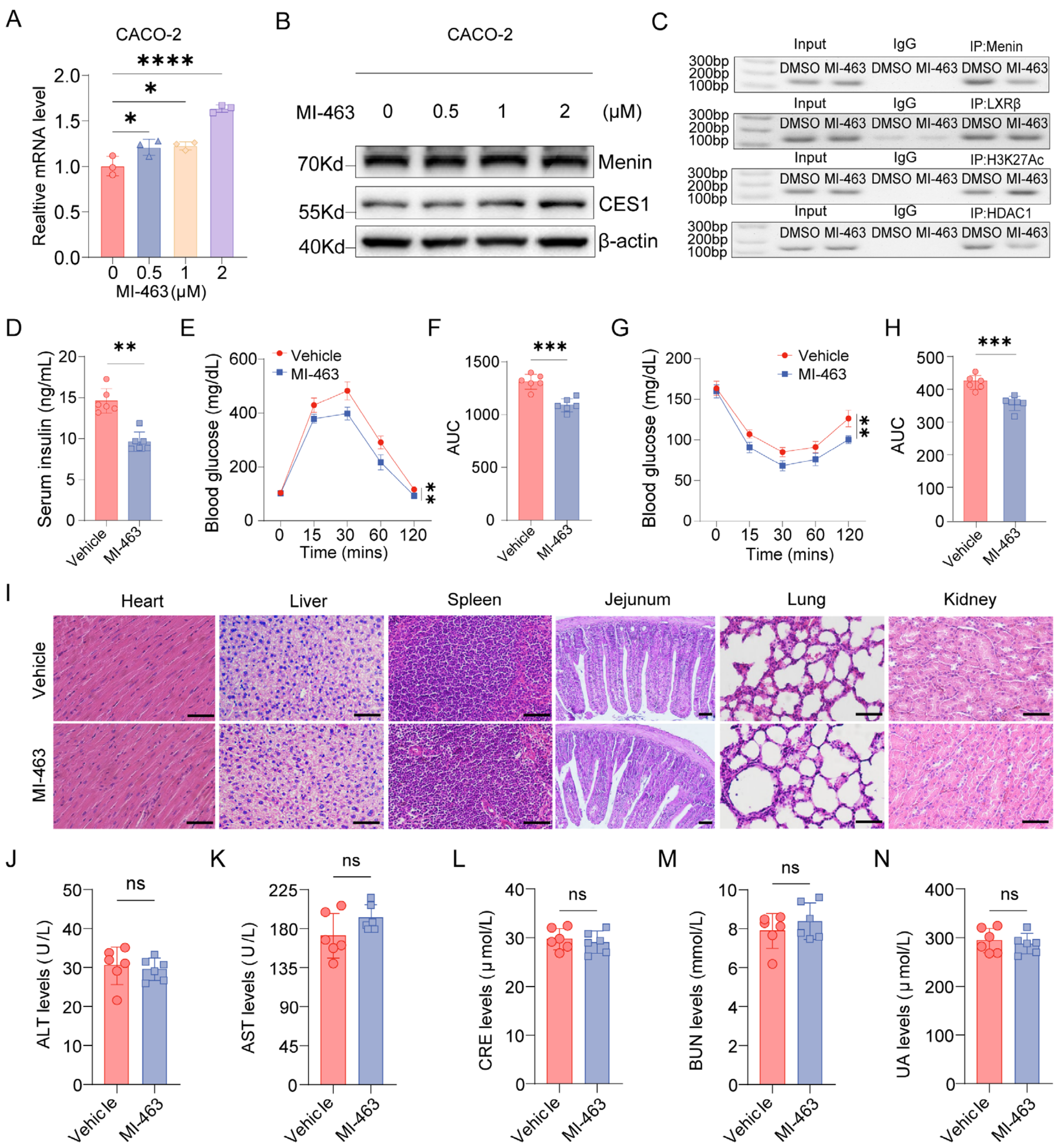

Supplemental Figure 6. MI-463 administration promotes *CES1* transcription and improved glucose intolerance without obvious toxicity.

(A) RT-qPCR analysis of *CES1* mRNA levels in CACO-2 cells treated with increasing concentrations of MI-463. (B) WB analysis of menin and CES1 protein levels in CACO-2 cells following MI-463 treatment at various concentrations. (C) ChIP-PCR analysis of the binding of menin, LXRβ, and H3K27Ac at the *Ces1g* promoter region in MODE-K cells treated with DMSO or MI-463. (D) Fasting serum insulin concentrations in vehicle and MI-463 treated mice. (E) IPGTT with glucose measurements at 0, 15, 30, 60, and 120 min in vehicle and MI-463 treated mice. (F)

IPGTT area under the curve. (G) IPITT with glucose measurements at the indicated time points in vehicle and MI-463 treated mice. (H) IPITT area under the curve. (I) Representative H&E staining of major organs, including heart, liver, spleen, jejunum, lung, and kidney, from mice treated with vehicle or MI-463, scale bars, 100 μm. (J–N) Serum biochemical analysis of alanine aminotransferase (ALT) (J), aspartate aminotransferase (AST) (K), creatinine (CRE) (L), blood urea nitrogen (BUN) (M), and uric acid (UA) (N) levels in vehicle and MI-463 treated mice. For A, $n = 3$; panel B and C show representative results from three independent experiments; for D-N, $n = 6$ mice per group. All data are presented as mean ± SD. Statistical significance was assessed by one-way ANOVA for panel A; by two-way ANOVA for panels E and G; and by unpaired Student's *t*-test for panels D, F, H, and J-N. *$P < 0.05$, **$P < 0.01$, ***$P < 0.001$, ****$P < 0.0001$; ns, not significant.

A

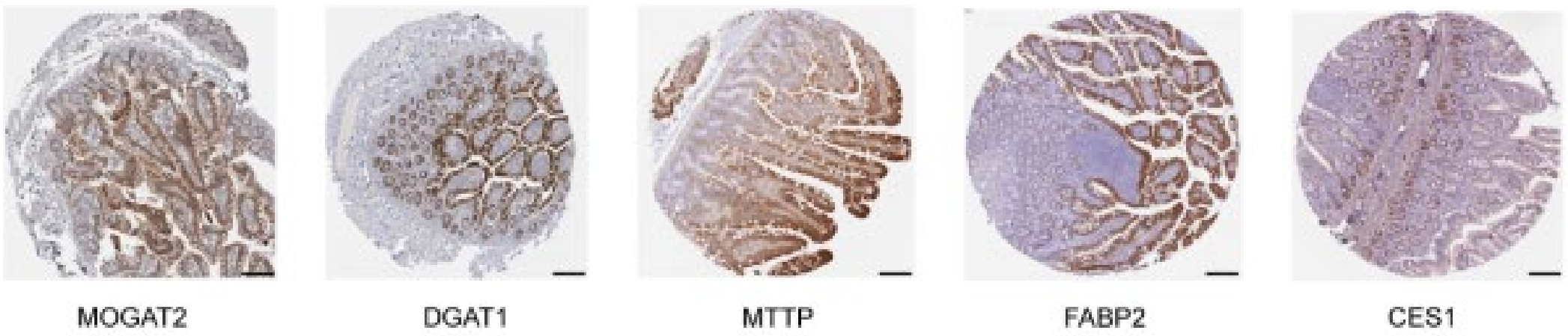

Supplemental Figure 7. Expression of lipid metabolism-related proteins in the human small intestine.

(A) Immunohistochemical staining of re-esterification enzymes (MOGAT2 and DGAT1), lipid transport proteins (MTTP and FABP2), and carboxylesterase CES1 in healthy human small intestine tissues, retrieved from the Human Protein Atlas. Scale bars, 200 μm.

3. Supplemental Tables

Supplementary Table 1. PCR primer sequences for mouse genotyping.

Genotyping Primer	Sequences
Villin Cre-Forward-1	CCATAGGAAGCCAGTTTCCCTTC
Villin Cre-Reverse-1	TTCCAGGTATGCTCAGAAAACGC
Villin Cre-Forward-2	CTATCAGGGATACTCCTCTTTGCC
Villin Cre-Reverse-2	GATACAGGAATGACAAGCTCATGGT
Villin CreERT2-Forward	TCCGTCTCTGGTGTAGCTGATGA
Villin CreERT2- Reverse	CCTGTTTCACTATCCAGGTTACG
Men1$^{f/f}$-Forward	GCACGGGCTTGACTAAGGAC
Men1$^{f/f}$-Reverse	CCCAATAACCCAACCCCAGA

Supplementary Table 2. Primer sequences for RT-qPCR.

RT-qPCR primer	Sequences
Human-*MEN1*-F	GCCTGGGTAGTGTTTGGGC
Human-*MEN1*-R	AGCGCATGTATGATCCTTTCAG
Human-*ACTIN*-F	CATGTACGTTGCTATCCAGGC
Human-*ACTIN*-R	CTCCTTAATGTCACGCACGAT
Human-*CES1*-F	GTGGCTCCGTGCCTTTAT
Human-*CES1*-R	TGTTCTCCTTTCGGTTTGT
Human-*CPT1A*-F	ATCAATCGGACTCTGGAAACGG
Human-*CPT1A*-R	TCAGGGAGTAGCGCATGGT
Human-*ABCA1*-F	GCTCTCAGACCTGGGCATTT
Human-*ABCA1*-R	CTTCGGCCACCTTGAGGAAT
Mouse-*Men1*-F	GCCTGACCTGGTGCTCCTGT
Mouse-*Men1*-R	GCGGTGAATCGGGCATAG
Mouse-*actin*-F	GTGACGTTGACATCCGTAAAGA
Mouse-*actin*-R	GCCGGACTCATCGTACTCC
Mouse-*Tjp1*-F	GTTGGTACGGTGCCCTGAAAGA
Mouse-*Tjp1*-R	GCTGACAGGTAGGACAGACGAT
Mouse-*Astb*-F	GTCTGTCCCCCAAATGCAACT
Mouse-*Astb*-R	CACCCCATAGAAAACATCACCA
Mouse-*Osta*-F	AGGCAGGACTCATATCAAACTTG
Mouse-*Osta*-R	TGAGGGCTATGTCCACTGGG
Mouse-*Ostb*-F	AGATGCGGCTCCTTGGAATTA
Mouse-*Ostb*-R	TGGCTGCTTCTTTCGATTTCTG
Mouse-*Pnlip*-F	CTGGGAGCAGTAGCTGGAAG
Mouse-*Pnlip*-R	AGCGGGTGTTGATCTGTGC
Mouse-*Clps*-F	GAACAGTATGCAGTGTAAGAGCA
Mouse-*Clps*-R	GCAGATGCCATAGTTGGTGTTG
Mouse-*Cd36*-F	GGACATTGAGATTCTTTTCCTCTG
Mouse-*Cd36*-R	GCAAAGGCATTGGCTGGAAGAAC

Mouse-*Slc27a2*-F	TCCTCCAAGATGTGCGGTACT
Mouse-*Slc27a2*-R	TAGGTGAGCGTCTCGTCTCG
Mouse-*Mogat2*-F	TGGGAGCGCAGGTTACAGA
Mouse-*Mogat2*-R	CAGGTGGCATACAGGACGGA
Mouse-*Dgat1*-F	GCGCTACTTCCGAGACTACTT
Mouse-*Dgat1*-R	GGGCCTTATGCCAGGAAACT
Mouse-*Dgat2*-F	CTGTGCTCTACTTCACCTGGCT
Mouse-*Dgat2*-R	CTGGATGGGAAAGTAGTCTCGG
Mouse-*Apoa4*-F	CAGAAGACGGATGTCACTCAGC
Mouse-*Apoa4*-R	AGCTGTACGACAAAGGGCACCA
Mouse-*Mttp*-F	CCAGGAAAGGTTCCTCTATGCC
Mouse-*Mttp*-R	GACTCTCTGATGTCGTTGCTTGC
Mouse-*Fabp1*-F	AGCTGTGGAAAGGAAGCCTCGTTGC
Mouse-*Fabp1*-R	GCACGATTTCTGACACCCCCTTGAT
Mouse-*Fabp2*-F	CATTCGACGGCACGTGGAAAGTAGA
Mouse-*Fabp2*-R	GCTTGGCCTCAACTCCTTCATATGT
Mouse-*Sar1b*-F	GATGAAACCATTGCCAACGTGCCT
Mouse-*Sar1b*-R	CCAGCGGAAGCCTTCTCCATAGCC
Mouse-*Vamp7*-F	GCTGTTGTTGCCAGGGGAACCACTA
Mouse-*Vamp7*-R	CCACAAGGTTTTCTGTTTTATCTAT
Mouse-*Ces1c*-F	GGTGGACTGGTGATAGGC
Mouse-*Ces1c*-R	ATCTTGGACCCAGCGTAG
Mouse-*Ces1d*-F	CAGAGGATGAACTACTGGAGACC
Mouse-*Ces1d*-R	CTGGTGCCTTTGGCAGAACTAC
Mouse-*Ces1g*-F	GGTACACACCGTTCATGGCAA
Mouse-*Ces1g*-R	GGGTTTTGGTAGCACAAAGGAG
Mouse-*Cpt1a*-F	AGCAAGATAGGCATAAACGCAGAG
Mouse-*Cpt1a*-R	CAAAGGTGTCAAATGGGAAGGAAT
Mouse-*Acadm*-F	AGCCTTTACTGGATTCATTGTGGA
Mouse-*Acadm*-R	TCCAGGGCATACTTCGTGGCTTCGT
Mouse-*Acadl*-F	AGTGAGTAGAGAGGTCTGGGAAAAA
Mouse-*Acadl*-R	CAGCCATTAGTGATGAACACCTTGC
Mouse-*Acox1*-F	CACCCGTCCCAAGAACTCCAGATA
Mouse-*Acox1*-R	AGGCATGTAACCCGTAGCACTCCC
Mouse-*Ehhadh*-F	TGGGCGATTTGGTCAGAAGACAGG
Mouse-*Ehhadh*-R	CCTCCAAGATGCGGAATGCCTCGTT
Mouse-*Abca1*-F	CAGTGGCGGCAACAAACG
Mouse-*Abca1*-R	TACTATGAGATGTAAGGACT
Mouse-*Npc1l1*-F	CCACAGACCCTGTGGAACTGT
Mouse-*Npc1l1*-R	GCTCGTCATGGAAAGCCTTT
Mouse-*Hmgcr*-F	AGCTTGCCCGAATTGTATGTG
Mouse-*Hmgcr*-R	TCTGTTGTGAACCATGTGACTTC
Mouse-*Sqle*-F	ATAAGAAATGCGGGGATGTCAC
Mouse-*Sqle*-R	ATATCCGAGAAGGCAGCGAAC

Mouse-*Scap*-F	CATCTTAGCCTGCTGCTACCC
Mouse-*Scap*-R	TCTGTTGGATGTACGCCACG
Mouse-*Ldlr*-F	TGACTCAGACGAACAAGGCTG
Mouse-*Ldlr*-R	ATCTAGGCAATCTCGGTCTCC
Mouse-*Acat1*-F	GAAACCGGCTGTCAAAATCTGG
Mouse-*Acat1*-R	TGTGACCATTTCTGTATGTGTCC
Mouse-*Acat2*-F	ACAAGACAGACCTCTTCCCTC
Mouse-*Acat2*-R	ATGGTTCGGAAATGTTCACC
Mouse-*Abcg5*-F	TGTGCATCTTAGGCAGCTCA
Mouse-*Abcg5*-R	CACAGTGAGGCTGCTCAGAA
Mouse-*Abcg8*-F	CCTCATCATTGGCTTCCTTAC
Mouse-*Abcg8*-R	ATTGACCTCTCCGAGTGACATT
Mouse-*Abcg1*-F	CTTTCCTACTCTGTACCCGAGG
Mouse-*Abcg1*-R	CGGGGCATTCCATTGATAAGG
Mouse-*Acly*-F	GTGGTCTGCTGGTGTATCGG
Mouse-*Acly*-R	CCTGGTTCTTGGCTACTGC
Mouse-*Fasn*-F	GGAGGTGGTGATAGCCGGTAT
Mouse-*Fasn*-R	TGGGTAATCCATAGAGCCCAG
Mouse-*Acaca*-F	ATGGGCGGAATGGTCTCTTTC
Mouse-*Acaca*-R	TGGGGACCTTGTCTTCATCAT
Mouse-*Srebf1*-F	GCAGCCACCATCTAGCCTG
Mouse-*Srebf1*-R	CAGCAGTGAGTCTGCCTTGAT
Mouse-*Srebf2*-F	GCAGCAACGGGACCATTCT
Mouse-*Srebf2*-R	CCCCATGACTAAGTCCTTCAACT
Mouse-*Scd1*-F	CAGTGCCGCGCATCTCTAT
Mouse-*Scd1*-R	GGTGTGGTGGTAGTTGTGGA

Supplementary Table 3. Primer sequences for dual-luciferase reporter and mutant plasmids of *Ces1g* and *CES1* promoters.

Promoter Primer	Sequences
Mouse-*Ces1g*-Promoter-F	TGTGCTCCCAACTCGTTTTAGTTG
Mouse-*Ces1g*-promoter-R	CCCTCTGCCATAACTTCGTTGCTT
Ces1g -Promoter-MutF1	GGCCTAACTGGCCGGTACCTGTGCTCCCAACTCGTTTTAGTTG
Ces1g -Promoter-MutR1	TGAGCTTTTGTGCCATGTGGGTTTCTTTTTAGGCCAGCAATTCATTCCTGAATGA
Ces1g -Promoter-MutF2	TCATTCAGGAATGAATTGCTGGCCTAAAAAGAAACCCACATGGCACAAAAGCTCA
Ces1g -Promoter-MutR2	CTCTGTCTTCTTCTACTCTACTCTAAAAAAAGAGAGATCACTATTTTAAAGAACAG
Ces1g -Promoter-MutF3	CTGTTCTTTAAAATAGTGATCTCTCTAAAATTAGAGTAGAGTAGAAGAAGACAGAG
Ces1g -Promoter-MutR3	TTATATACCCTCTAGTGTCTAAGCTTCCCTCTGCCATAACTTCGTTGCTT
CES1-promoter-F	AGACAGCATTAATCTCACGGGGTT
CES1-promoter-R	TGTTTCAGAGAGGGGGTAACCCAA
CES1-Promoter-MutF1	GGCCTAACTGGCCGGTACCAGACAGCATTAATCTCACGGGGTT
CES1-Promoter-MutR1	CTTTAAAAAGGTAAACTTTATAATATATAATTTATATTTCAATAAAATAATG
CES1-Promoter-MutF2	CATTATTTTATTGAAATATAAATTATATATTATAAAGTTTACCTTTTTAAAG
CES1-Promoter-MutR2	CCAAGTACCCATCCATTTTGTAAATATTTATTAAAATGCTTGGCACCTGGCC
CES1-Promoter-MutF3	GGCCAGGTGCCAAGCATTTTAATAAATATTTACAAAATGGATGGGTACTTGG
CES1-Promoter-MutR3	TTATATACCCTCTAGTGTCTAAGCTTTGTTTCAGAGAGGGGGTAACCCAA

Supplementary Table 4. Primer sequences for gene editing mediated by shRNA and sgRNA.

shRNA or sgRNA name	Sequences
Men1-shRNA#1 sense	CCGGTGGAGATCTACAAGGAATTCCTCGAGGAATTCCTTGTAGATCTCCTTTTTG
Men1-shRNA#1 anti-sense	AATTCAAAAAGGAGATCTACAAGGAATTCCTCGAGGAATTCCTTGTAGATCTCCA
Men1-shRNA#2 sense	CCGGTGCTCCGACTCTTATCTGTGAACTCGAGTTCACAGATAAGAGTCGGAGCTTTTTG
Men1-shRNA#2 anti-sense	AATTCAAAAAGCTCCGACTCTTATCTGTGAACTCGAGTTCACAGATAAGAGTCGGAGCA
Ces1g-Oligo- sense1	CACCGGTACCACAGGCGGTAAGGAT
Ces1g-Oligo- anti-sense1	AAACCATGGTGTCCGCCATTCCTAC
Ces1g-Oligo- sense2	CACCGACACCCATCCTTACCGCCTG
Ces1g-Oligo- anti-sense2	AAACTGTGGGTAGGAATGGCGGACC
CES1-Oligo- sense1	CACCGAGAGCTATTTACAAACCGAA
CES1-Oligo- anti-sense1	AAACTTCGGTTTGTAAATAGCTCTC
CES1-Oligo- sense2	CACCGGCATCCGTCCTCGCCACCTG
CES1-Oligo- anti-sense2	AAACCAGGTGGCGAGGACGGATGCC

Supplementary Table 5. Primer sequences for transcription factors and different truncated forms of LXRβ plasmids.

Transcription factor	Sequences
RELA-F-homo	AGACTACGCATCAGCGGAAAAGCTTATGGACGAACTGTTCCCCCT
RELA-R-homo	CACTGTGCTGGATATCTGCAGAATTCTTAGGAGCTGATCTGACTCA
PPARγ-F-homo	AGACTACGCATCAGCGGAAAAGCTTATGGGTGAAACTCTGGGAGAT
PPARγ-R-homo	CACTGTGCTGGATATCTGCAGAATTCCTAGTACAAGTCCTTGTAG
HNF4α-F-homo	CCCAGACTACGCATCAGCGGAAAAGCTTATGCGACTCTCCAAAACC
HNF4α-R-homo	CTGTGCTGGATATCTGCAGAATTCTTAAGCAACTTGCCCAAAGCG
LXRα-F-homo	CCAGACTACGCATCAGCGGAAAAGCTTATGTCCTTGTGGCTGGGG
LXRα-R-homo	CTGTGCTGGATATCTGCAGAATTCTCATTCGTGCACATCCCAG
LXRβ-F-homo	CAGACTACGCATCAGCGGAAAAGCTTATGTCCTCTCCTACCACG
LXRβ-R-homo	ACTGTGCTGGATATCTGCAGAATTCTCACTCGTGGACGTCCCAGAT

Supplementary Table 6. Primer sequences for CHIP-qPCR.

CHIP-Primer	Sequences
Ces1g-CHIP-F0	GCATTATCTCCACCACCAGACACC
Ces1g-CHIP-R0	CCTGAAATCCTGCCCACTAA
Ces1g-CHIP-F1	TCCAATGGCTGGTCAAATAA
Ces1g-CHIP-R1	CCTGAAATCCTGCCCACTAA
Ces1g-CHIP-F2	GCCTGCTGTGCTACTCTCTGCCTT
Ces1g-CHIP-R2	CTCTTATTTGACCAGCCATT
Ces1g-CHIP-F3	GCCCAGGGATGCCAAGAGATGTAT
Ces1g-CHIP-R3	AAAGGCAGAGAGTAGCACAGCAGG
Ces1g-CHIP-F4	GAAGAAGACAGAGCACTTGATAGG
Ces1g-CHIP-R4	ATCTCTTGGCATCCCTGGGCTTGT
Ces1g-CHIP-F5	ATAATGGACGGATAATGAATAGGC
Ces1g-CHIP-R5	CAATACTCCTATCAAGTGCTCTGT
Ces1g-CHIP-F6	GTCTCTCACTGAAGAAAACTACAT
Ces1g-CHIP-R6	TATCCGTCCATTATTCAAACATTA
Ces1g-CHIP-F7	AAGAAGATACTGTAGACCCTTAGG
Ces1g-CHIP-R7	TGAGAGACTAAACTGCCCCACACT
Ces1g-CHIP-F8	CAGGAATGAATTGCTGGCCTAGGT
Ces1g-CHIP-R8	GGCACTTTCATAAATATGCCTTAA
CES1-CHIP-F1	TGGATGATAACAGCCAGGGACT
CES1-CHIP-R1	AGCTTCACAATATCCTGCCAAC
Ces2a-CHIP-F1:	CTGTGGCTTCTCCTGTTG
Ces2a-CHIP-R1:	GGCATCTGAAGTCCTTCTG
Ces2a-CHIP-F2:	TGCAGAAGGACTTCAGATG
Ces2a-CHIP-R2:	GGAGACAAGAGCCAACAA
Ces2a-CHIP-F3:	CAATCAAATGTCCACACCTAC
Ces2a-CHIP-R3:	CAATGGCATGGTTGAATCTC

Supplementary Table 7. Reagents, antibodies and kits used in this study.

Category	Reagent or Resource	Source
Reagents	Tamoxifen	Sigma-Aldrich, Cat# T5648
Reagents	DAPI staining solution	Thermo Fisher Scientific, Cat# D1306
Reagents	FITC-dextran	Sigma-Aldrich, Cat# FD4
Reagents	TRIzol reagent	Invitrogen, Cat# 15596026
Reagents	RIPA lysis buffer	Sigma-Aldrich, Cat# R0278
Reagents	BCA Protein Assay Kit	Thermo Scientific, Cat# 23225
Reagents	Dual-Luciferase® Reporter Assay System	Promega, Cat# E1910
Reagents	QuantiTect SYBR Green PCR Kit	Qiagen, Cat# 204143
Reagents	SuperScript III Reverse Transcriptase	Invitrogen, Cat# 18080044
Antibodies	Anti-HA magnetic beads	MCE, Cat# HY-K0201
Antibodies	Anti-Flag antibody	MCE, Cat# HY-P80111
Antibodies	Anti-HDAC1 antibody	Abcam, Cat# ab280198
Antibodies	Anti-HDAC3 antibody	Abcam, Cat# ab137704
Antibodies	Anti-Histone H3 (acetyl K27) antibody	Abcam, Cat# ab4729
Antibodies	Anti-mouse IgG, HRP-linked antibody	Cell Signaling Technology, Cat# 7076S
Antibodies	Anti-rabbit IgG, HRP-linked antibody	Cell Signaling Technology, Cat# 7074S
Antibodies	Anti-β-actin antibody	Cell Signaling Technology, Cat# 3700S
Antibodies	Liver Carboxylesterase 1 (CES1) antibody	Abmart, Cat# T58003s
Antibodies	menin antibody	Bethyl, Cat# A300-105A
Antibodies	CPT1A Polyclonal Antibody	Proteintech, Cat# 15184-1-AP
Antibodies	LXRβ monoclonal antibody	Proteintech, Cat# 60345-1-Ig
Antibodies	PerCP-Cyanine5.5 anti-mouse CD45 Antibody	BioLegend, Cat# 103132
Reagents	Zombie NIR™ Fixable Viability Kit	BioLegend, Cat# 423105
Reagents	6× SDS-PAGE loading buffer	Full Gold Biotechnology, Cat# DL101-2
Reagents	AgeI-HF® restriction enzyme	NEB, Cat# R3552S

Reagents	BCA Protein Assay Kit	Beyotime Biotechnology, Cat# P0012
Reagents	BD nonfat dry milk	BD, Cat# 232100
Reagents	Benzonase nuclease	HaiGene, Cat# C2001
Reagents	BODIPY 500/510 C1, C12	Beyotime Biotechnology, Cat# C2055-1mg
Reagents	DAPI staining solution	Beyotime Biotechnology, Cat# C1002
Reagents	DNA 1000 Marker	TAKARA, Cat# 3591A
Reagents	DNA Purification Buffers and Spin Columns	CST, Cat# 14209
Reagents	Dual-Luciferase® Reporter Assay System	Promega, Cat# E1960
Reagents	ECL chemiluminescent substrate	Proteintech, Cat# PK10003
Reagents	FastKing One Step RT-PCR Kit	TIANGEN, Cat# KR118-02
Reagents	Trypsin	Gibco/Thermo Fisher, Cat# 25200-072
Reagents	Hank's Balanced Salt Solution (HBSS)	Biosharp, Cat# BL562A
Reagents	HE Staining Kit	Biosharp, Cat# BL700B
Reagents	Lipoprotrein lipase inhibitor (Tyloxapol)	Sigma-Aldrich, Cat# T0307
Reagents	LXR antagonist (GSK2033)	Sigma-Aldrich, Cat# SML1617-5MG
Reagents	Mouse insulin ELISA Kit	Proteintech, Cat# KE10089
Reagents	NBD-TAG (1,3-diolein, 2-NBD-X ester)	Setareh Biotech, Cat# 6285
Reagents	L-α-Phosphatidylcholine	Proteintech, Cat# 8002-43-5
Reagents	Phosphatidylcholines,soya	MCE Cat# 97281-47-5
Reagents	Citrate Synthase Activity Assay Kit (Colorimetric)	Thermo Fisher Scientific, Cat# EEA054
Reagents	Fatty Acid Oxidation Colorimetric Assay Kit	Elabscience, Cat# E-BC-K784-M
Reagents	Extracellular Oxygen Consumption Rate Plate Assay Kit	Dojindo Molecular Technologies, Cat# E297
Reagents	Triglyceride Assay Kit	Nanjing Jiancheng Bioengineering Institute, Cat# A110-1-1
Reagents	Free Fatty Acid Assay Kit	Solarbio, Cat# BC0595

Reagents	Cholesterol Assay Kit	Nanjing Jiancheng Bioengineering Institute, Cat# A111-1-1
Reagents	XF Palmitate Oxidation Stress Test Kit	Agilent Technologies, Cat# 103693-100
Reagents	BODIPY 500/510 C1, C12	Biyun Tian Biotechnology Co., LTD, Cat# C2055-1mg
Reagents	Very low density lipoprotein (VLDL) assay kit	Nanjing Jiancheng Bioengineering Institute, Cat# H249-1-2
Reagents	Liver function ALT + AST assay kit, microplate method	Nanjing Jiancheng Bioengineering Institute, Cat# Z002-1-1
Reagents	Renal function Cr + UA + BUN assay kit, microplate method	Nanjing Jiancheng Bioengineering Institute, Cat# Z003-1-1
