# Supplementary material for: Inhibiting menin attenuates high-fat diet–induced weight gain by limiting intestinal lipid absorption in mice
Source: J Clin Invest. 2026 Aug 11;136(19):e202709. doi: 10.1172/JCI202709 (PMC13626824; doi:10.1172/JCI202709)
Supplement: Supplementary file 2 [file jci-136-202709-s051.pdf]

Original image of the protein bands

Figure2M

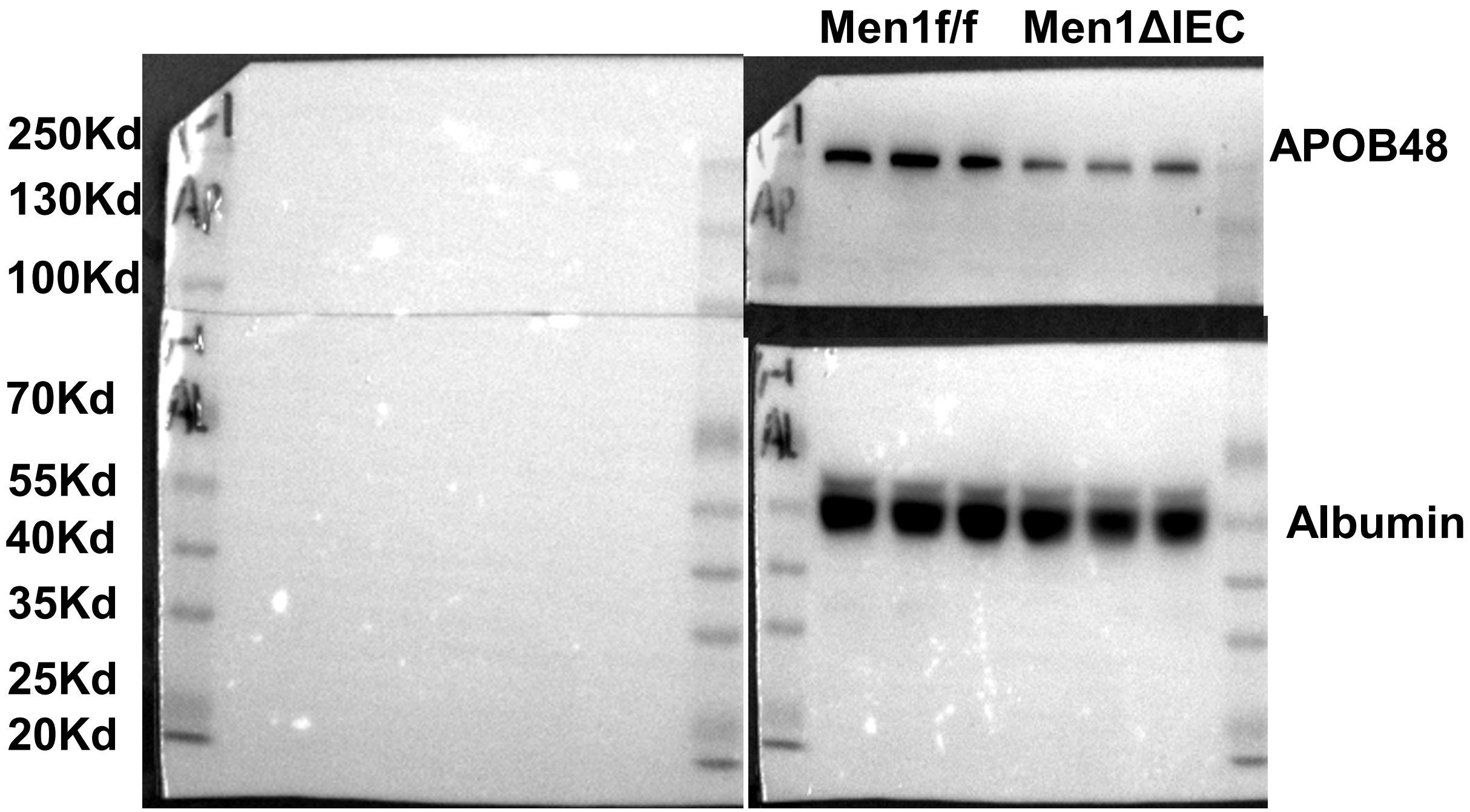

Men1f/f
Men1ΔIEC
250Kd
130Kd
100Kd
70Kd
55Kd
40Kd
35Kd
25Kd
20Kd
APOB48
Albumin

Figure3E

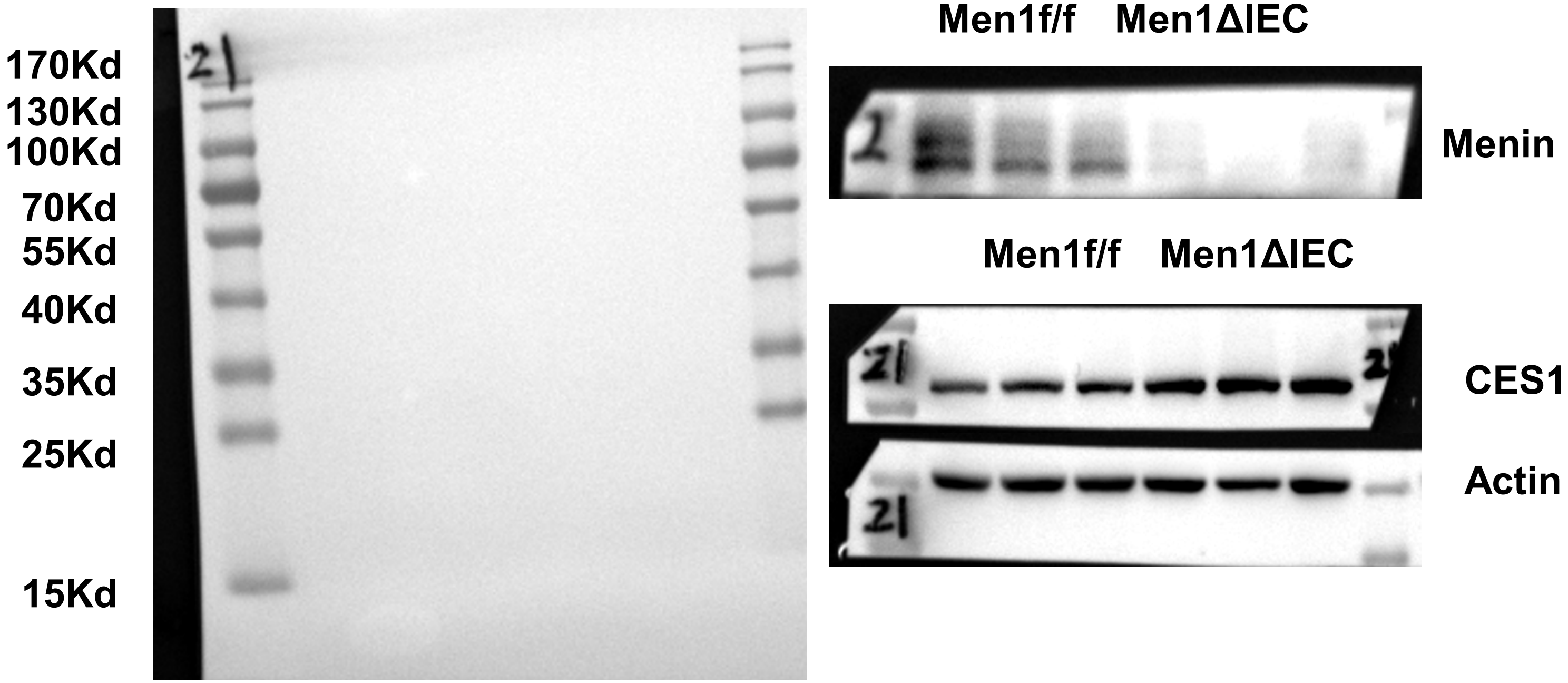

170Kd
130Kd
100Kd
70Kd
55Kd
40Kd
35Kd
25Kd
15Kd
Men1f/f
Men1ΔIEC
Menin
Men1f/f
Men1ΔIEC
CES1
Actin

Figure3G

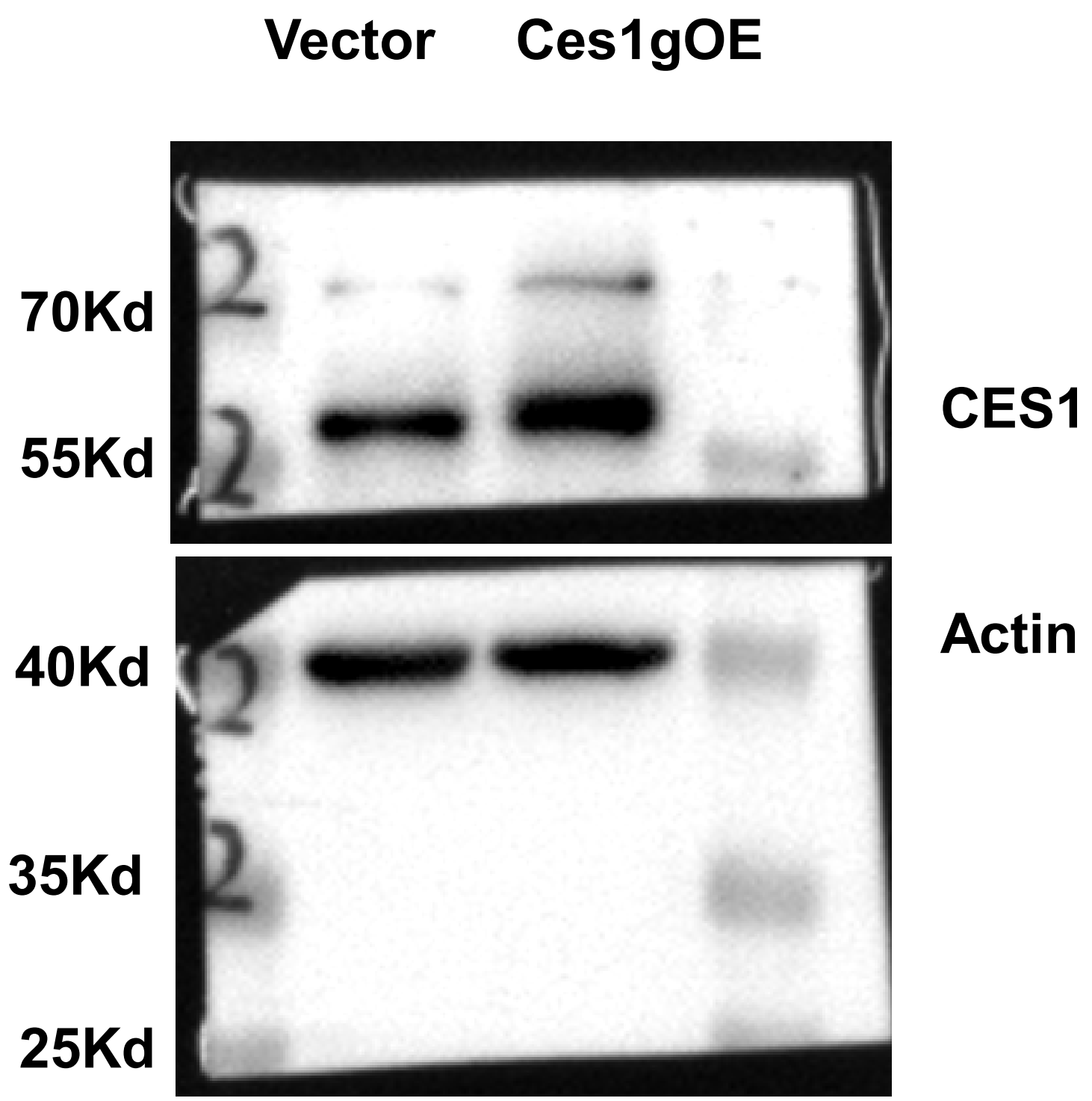

Figure3O

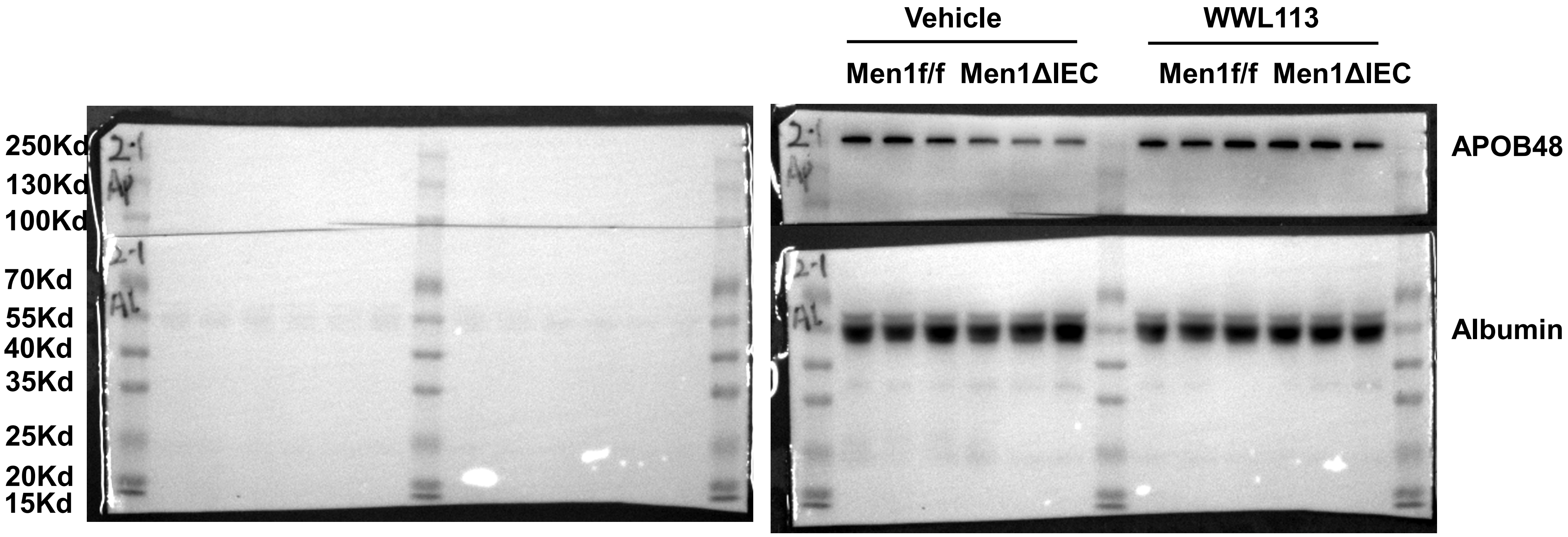

Vehicle
WWL113
Men1f/f
Men1ΔIEC
Men1f/f
Men1ΔIEC
250Kd
130Kd
100Kd
70Kd
55Kd
40Kd
35Kd
25Kd
20Kd
15Kd
APOB48
Albumin

Figure4C

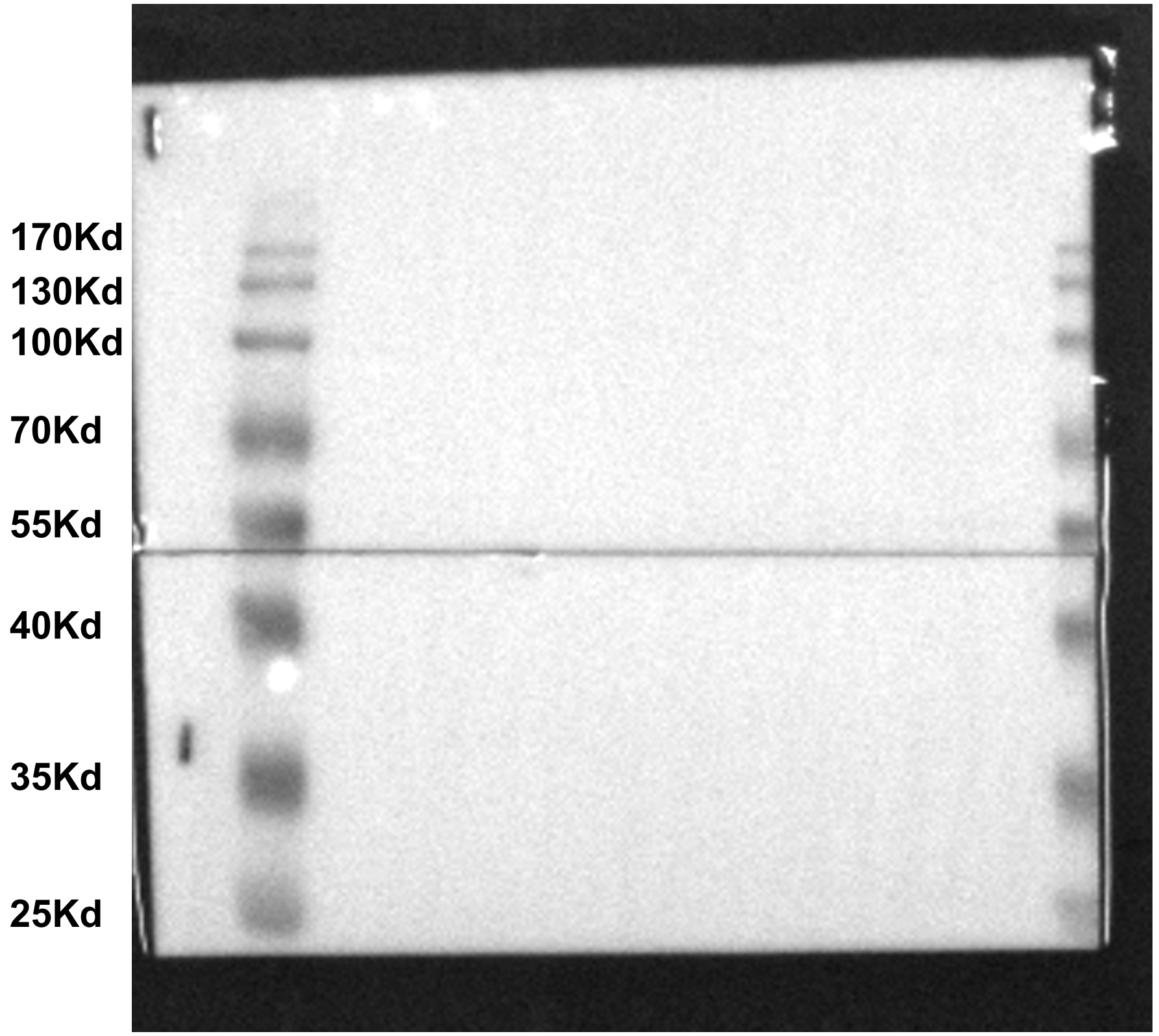

170Kd
130Kd
100Kd
70Kd
55Kd
40Kd
35Kd
25Kd

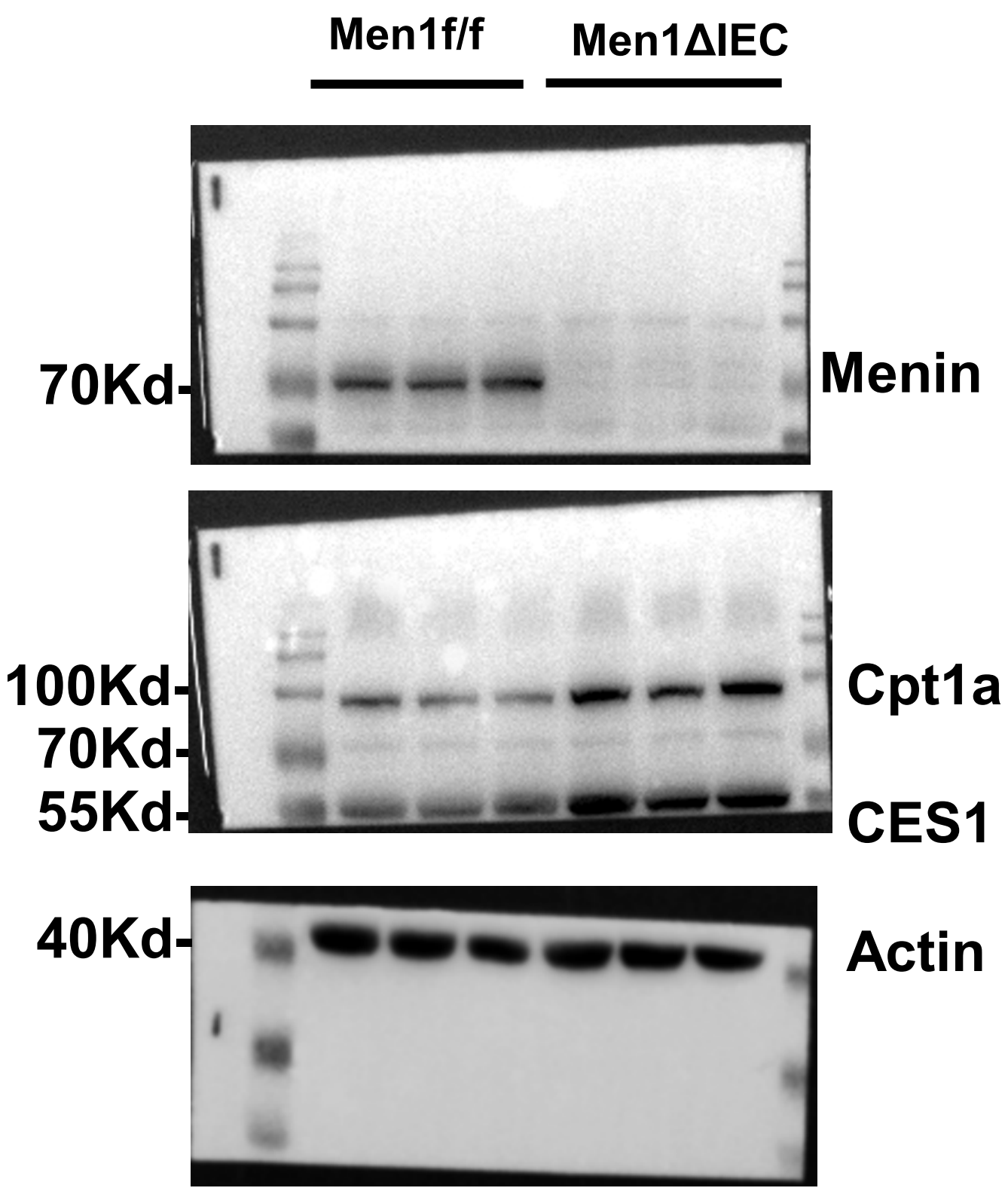

Men1f/f
Men1ΔIEC
70Kd-
Menin
100Kd-
70Kd-
55Kd-
Cpt1a
CES1
40Kd-
Actin

Figure5B

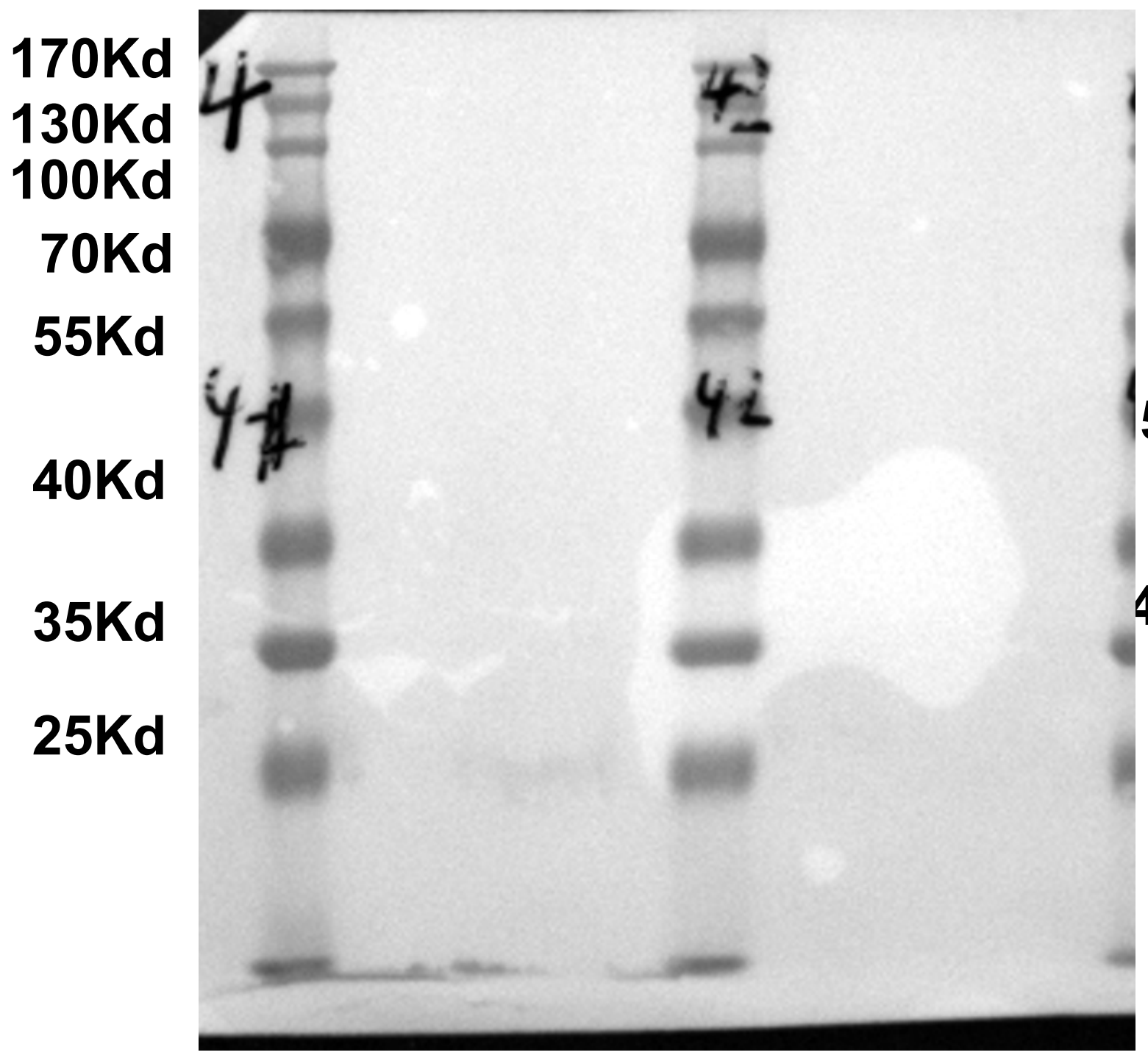
170Kd
130Kd
100Kd
70Kd
55Kd
40Kd
35Kd
25Kd

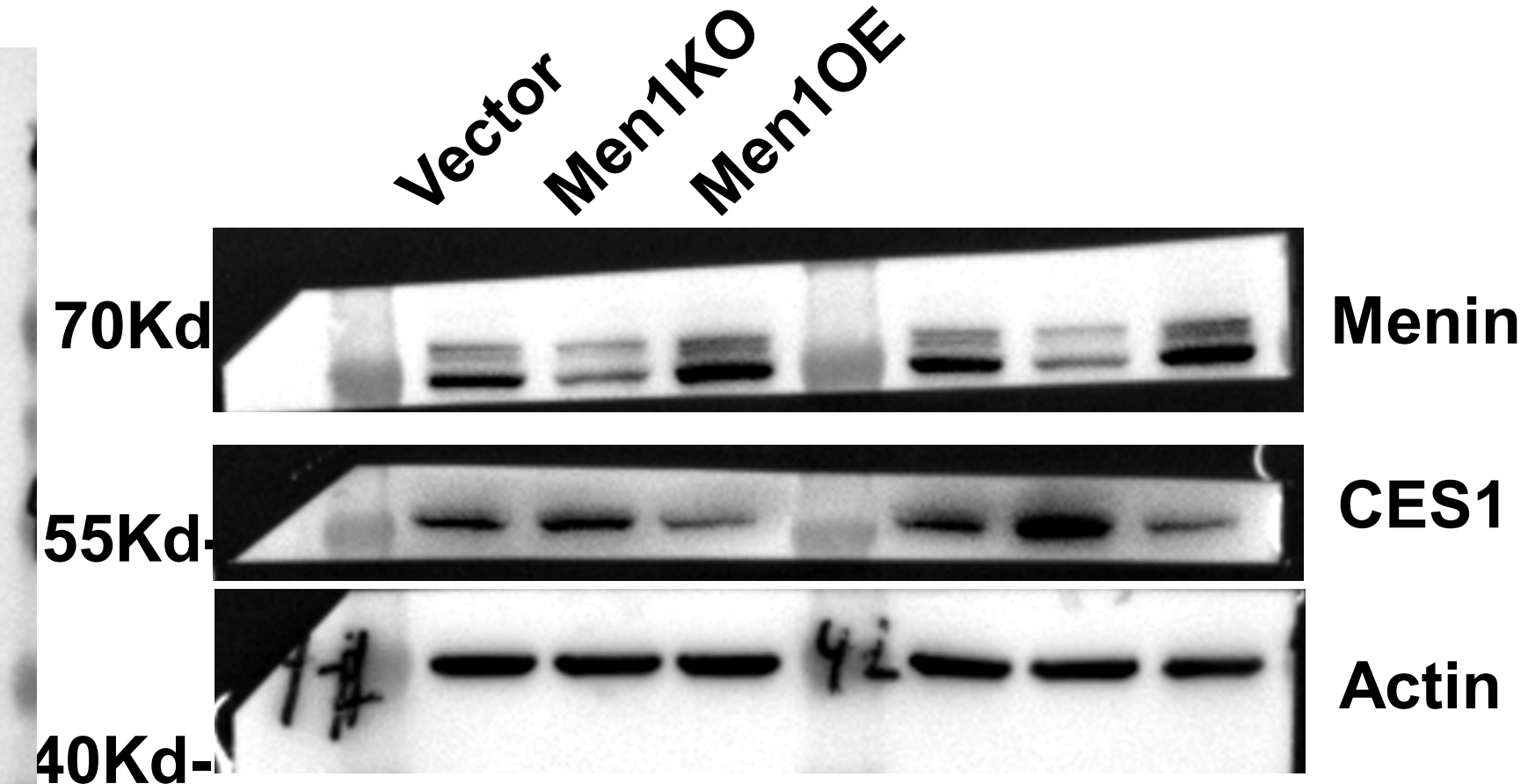
Vector
Men1KO
Men1OE
70Kd
55Kd-
40Kd-
Menin
CES1
Actin

Figure5F

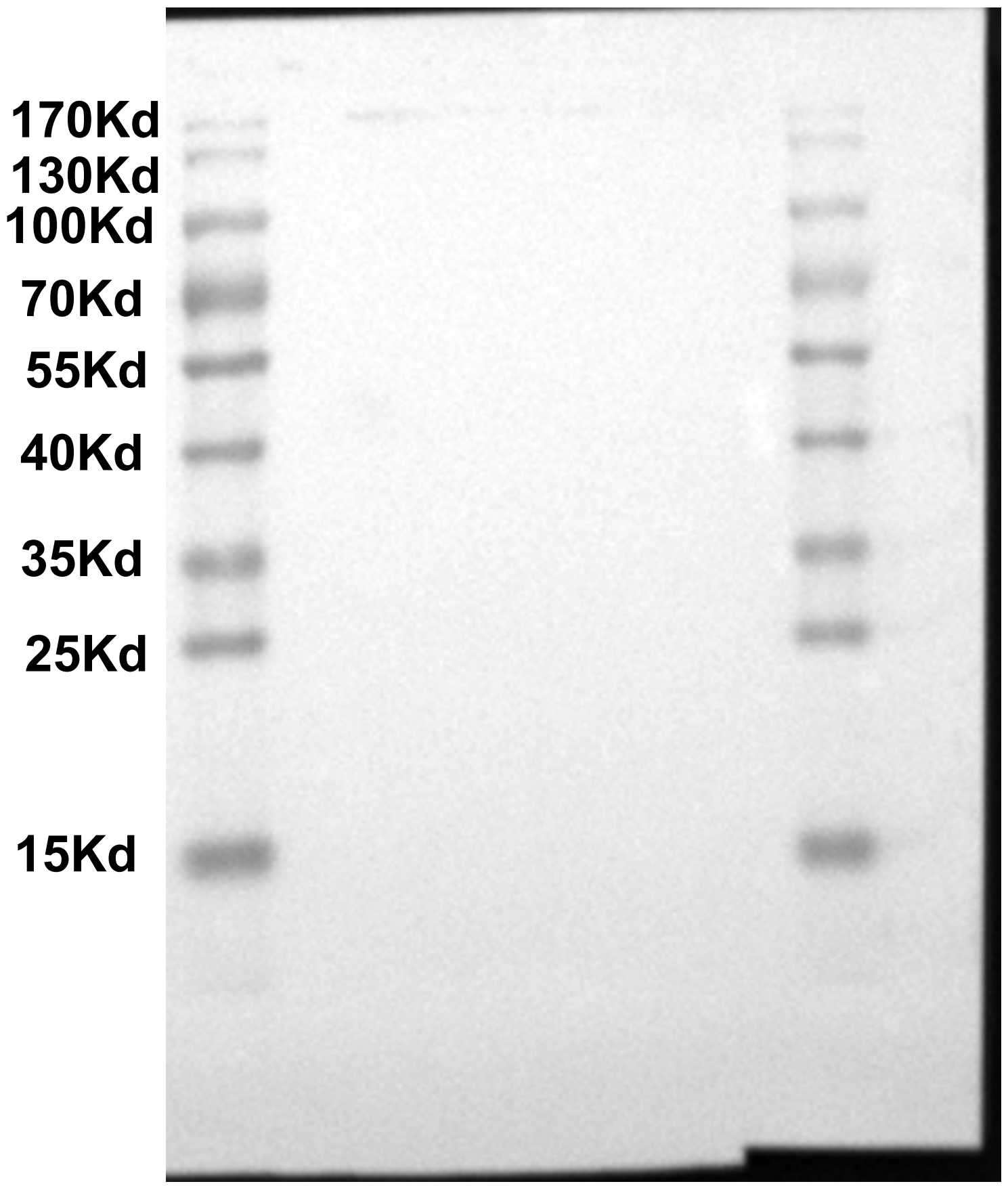
170Kd
130Kd
100Kd
70Kd
55Kd
40Kd
35Kd
25Kd
15Kd

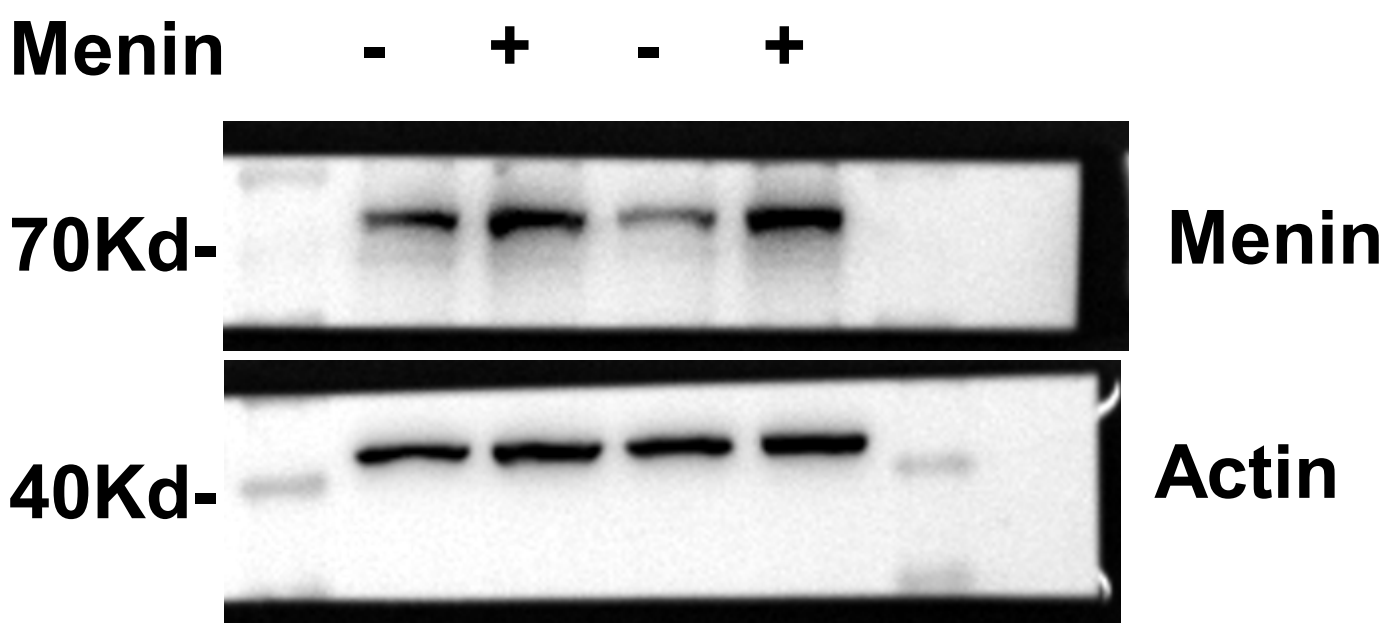
Menin - + - +
70Kd-
Menin
40Kd-
Actin

Figure5H

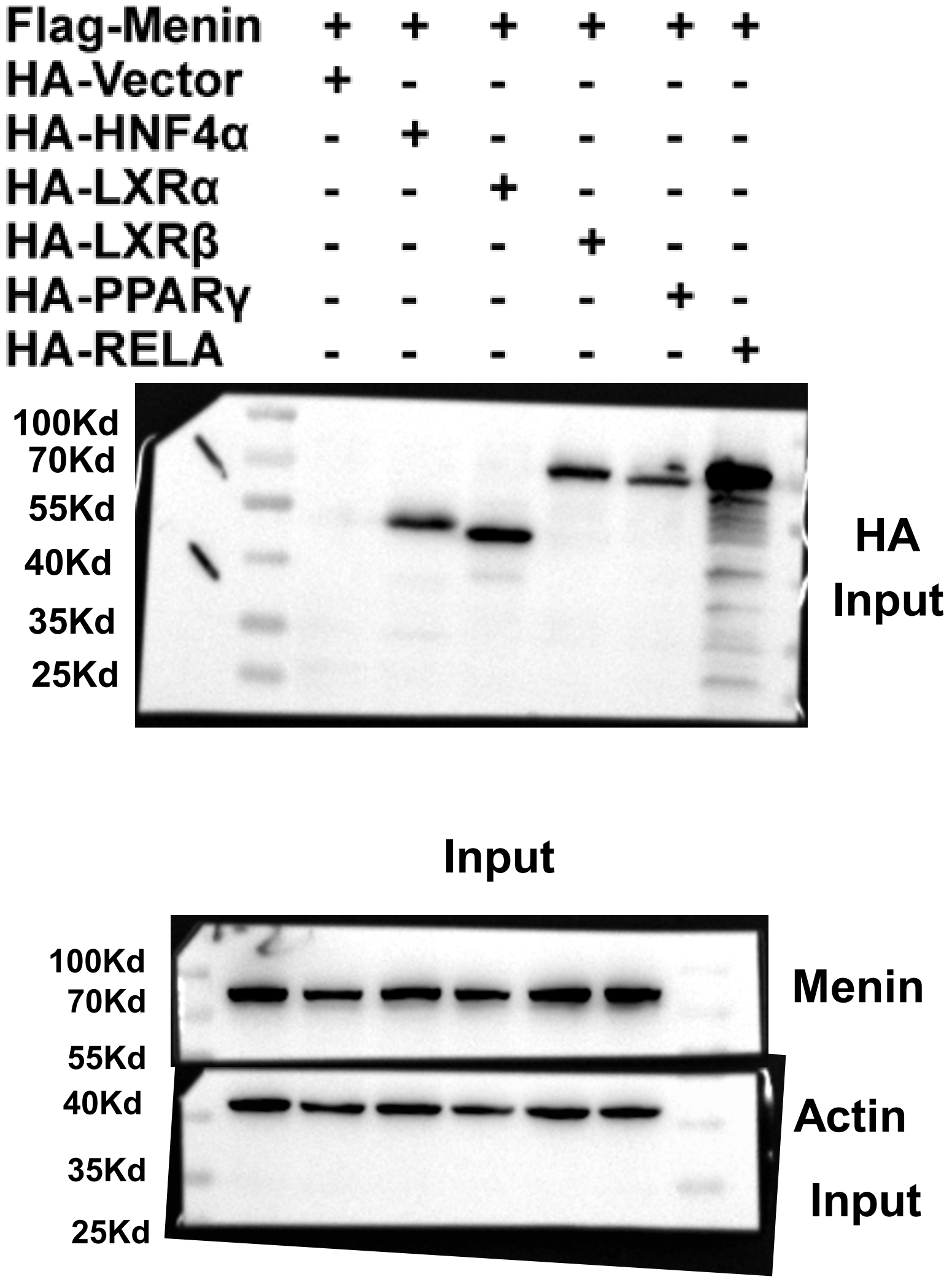

Flag-Menin + + + + + +
HA-Vector + - - - - -
HA-HNF4α - + - - - -
HA-LXRα - - + - - -
HA-LXRβ - - - + - -
HA-PPARγ - - - - + -
HA-RELA - - - - - +
100Kd
70Kd
55Kd
40Kd
35Kd
25Kd
HA
Input
Input
100Kd
70Kd
55Kd
40Kd
35Kd
25Kd
Menin
Actin
Input

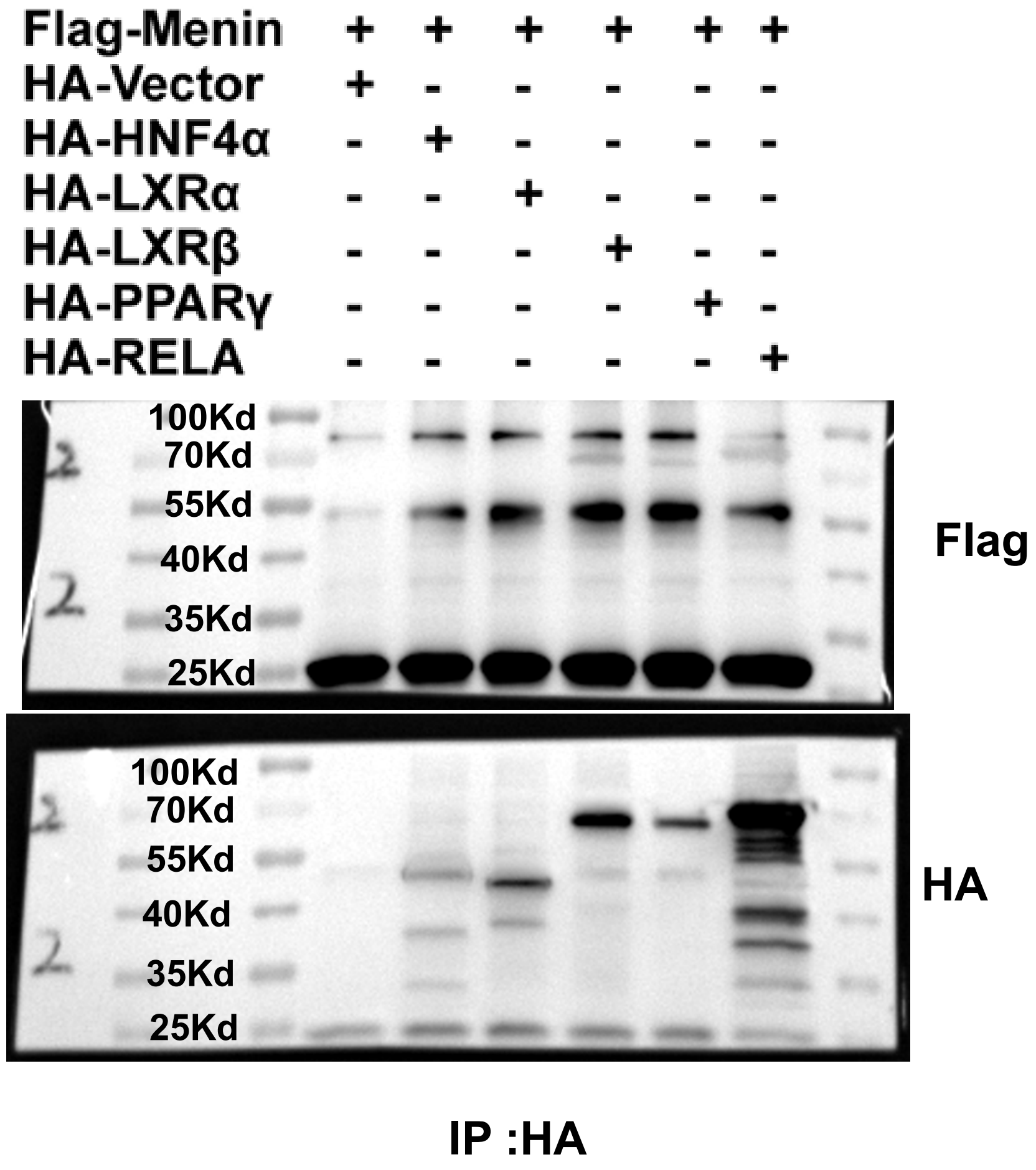

Flag-Menin + + + + + +
HA-Vector + - - - - -
HA-HNF4α - + - - - -
HA-LXRα - - + - - -
HA-LXRβ - - - + - -
HA-PPARγ - - - - + -
HA-RELA - - - - - +
100Kd
70Kd
55Kd
40Kd
35Kd
25Kd
Flag
100Kd
70Kd
55Kd
40Kd
35Kd
25Kd
HA
IP :HA

Figure5J

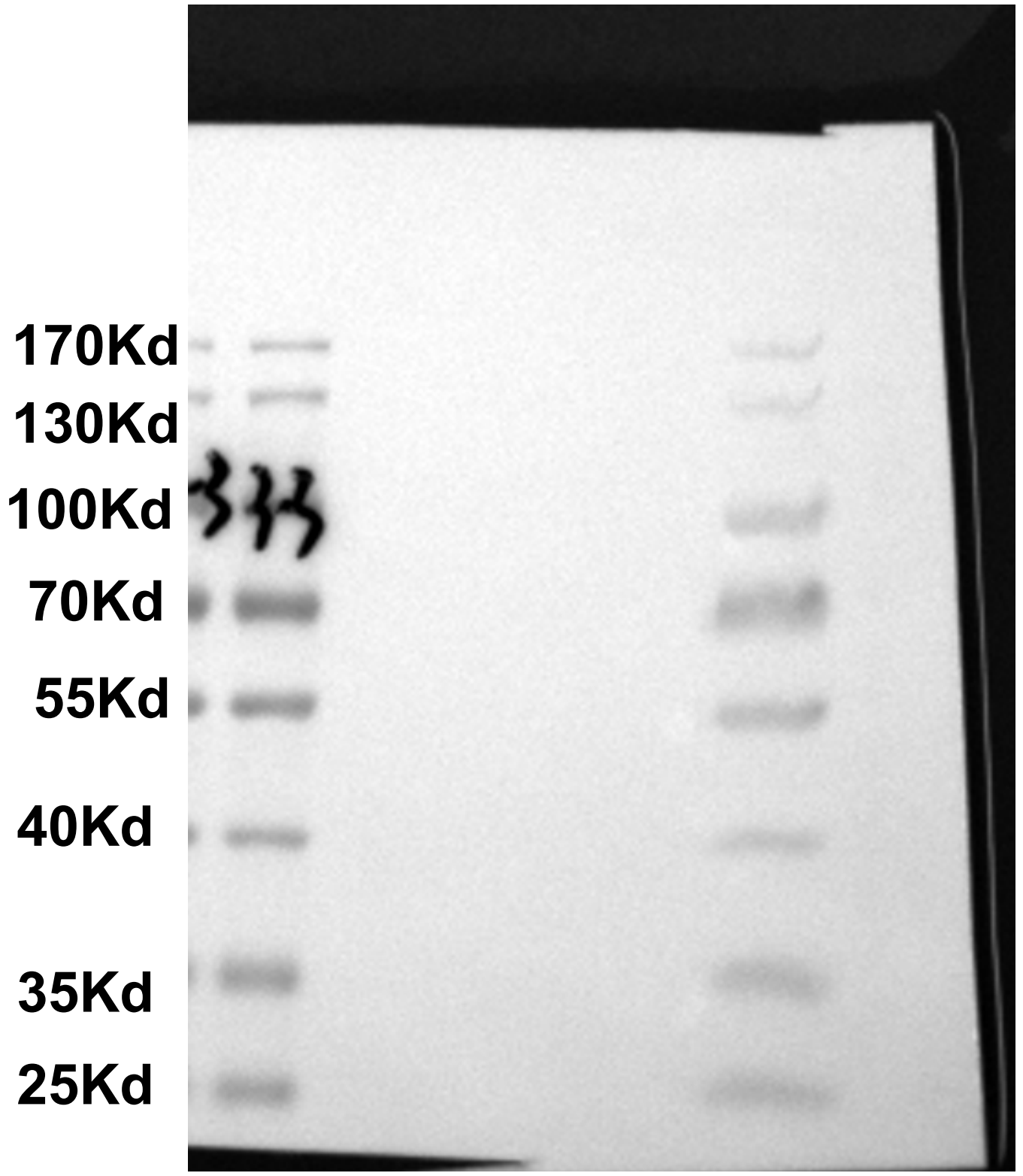

170Kd
130Kd
100Kd
70Kd
55Kd
40Kd
35Kd
25Kd

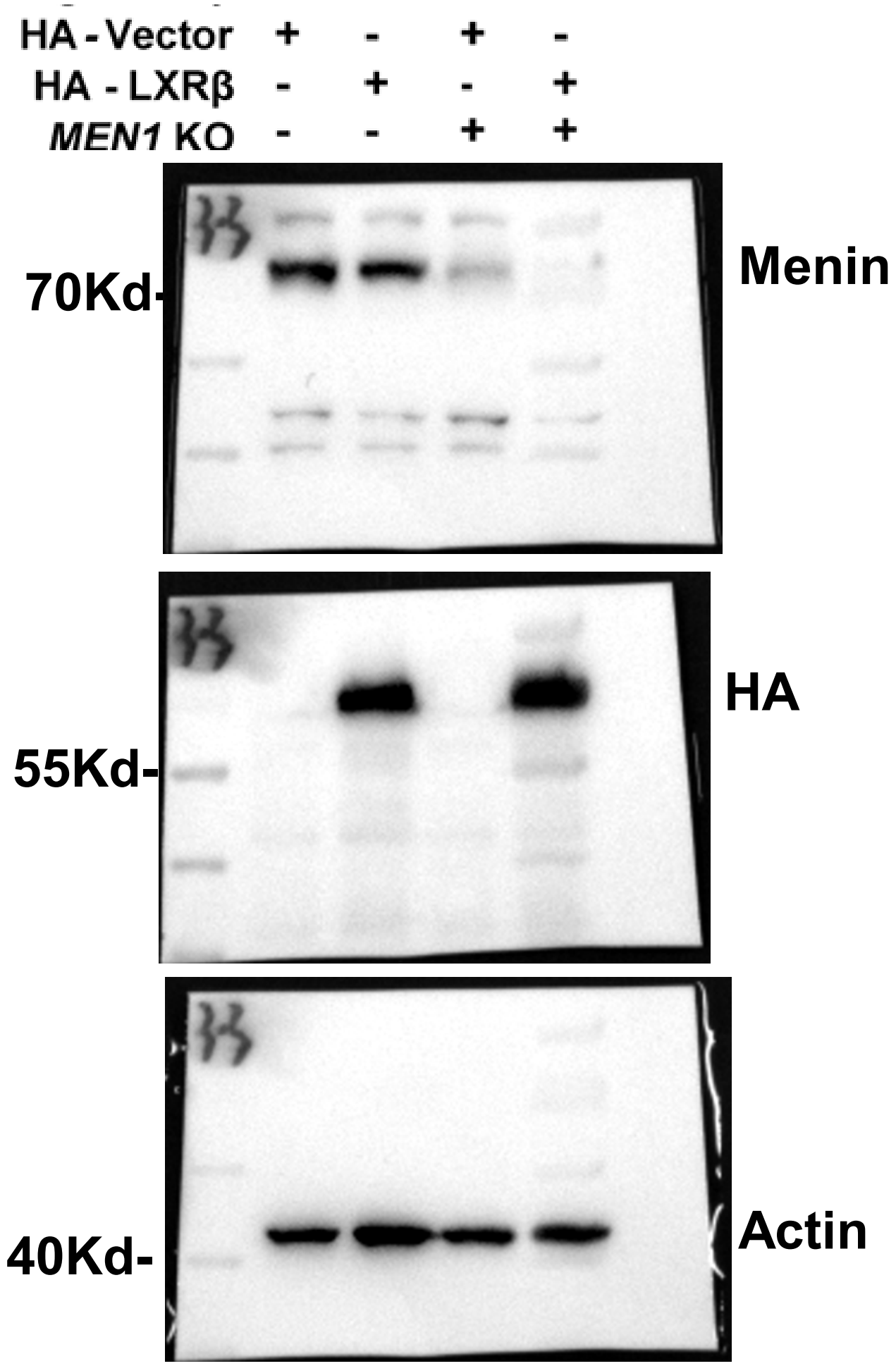

HA - Vector + - + -
HA - LXRβ - + - +
MEN1 KO - - + +
70Kd-
Menin
55Kd-
HA
40Kd-
Actin

Figure5O-HDAC3

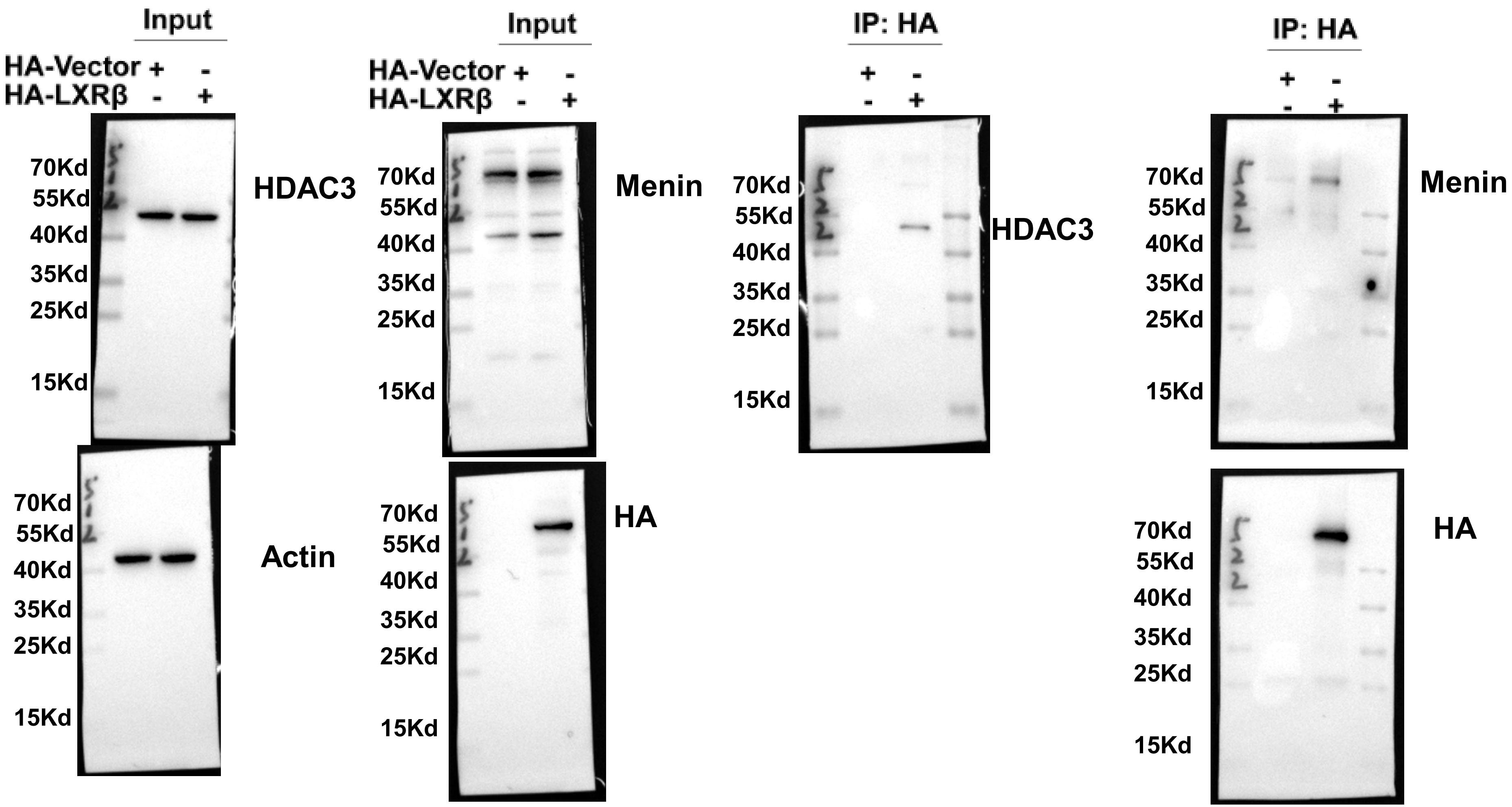

Figure5O-HDAC1

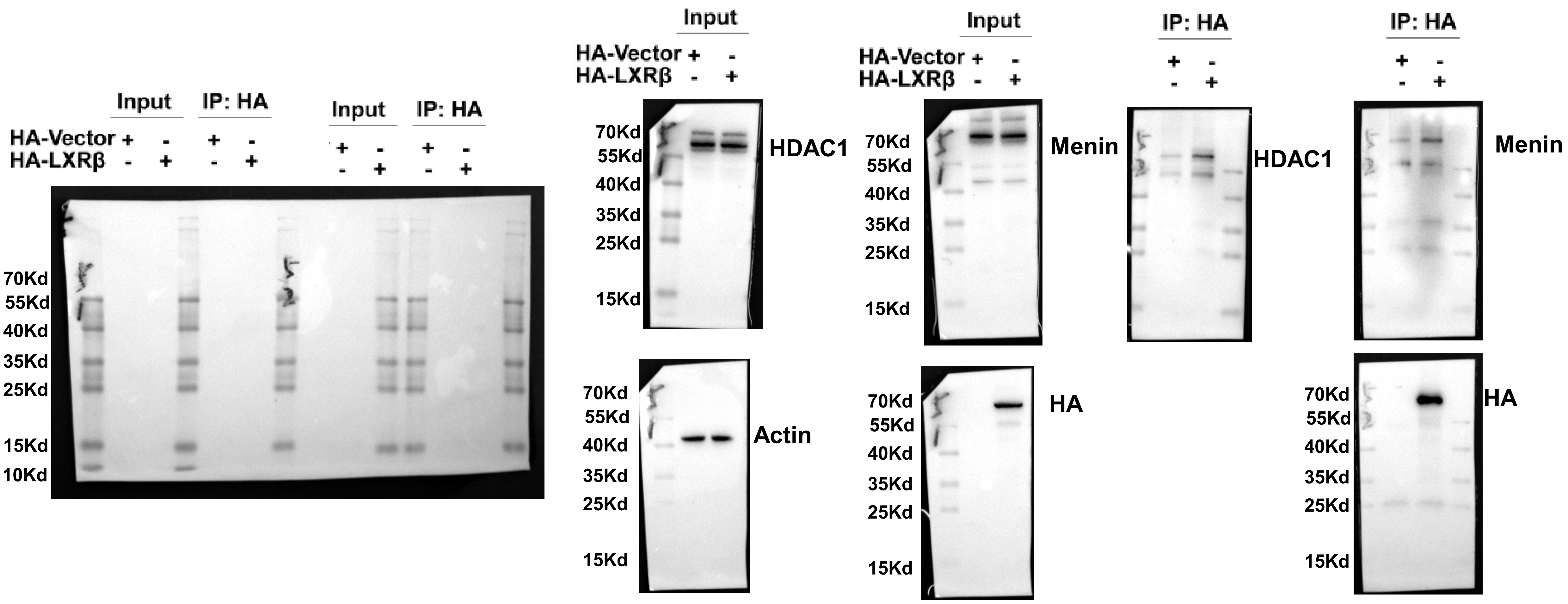

Figure6B

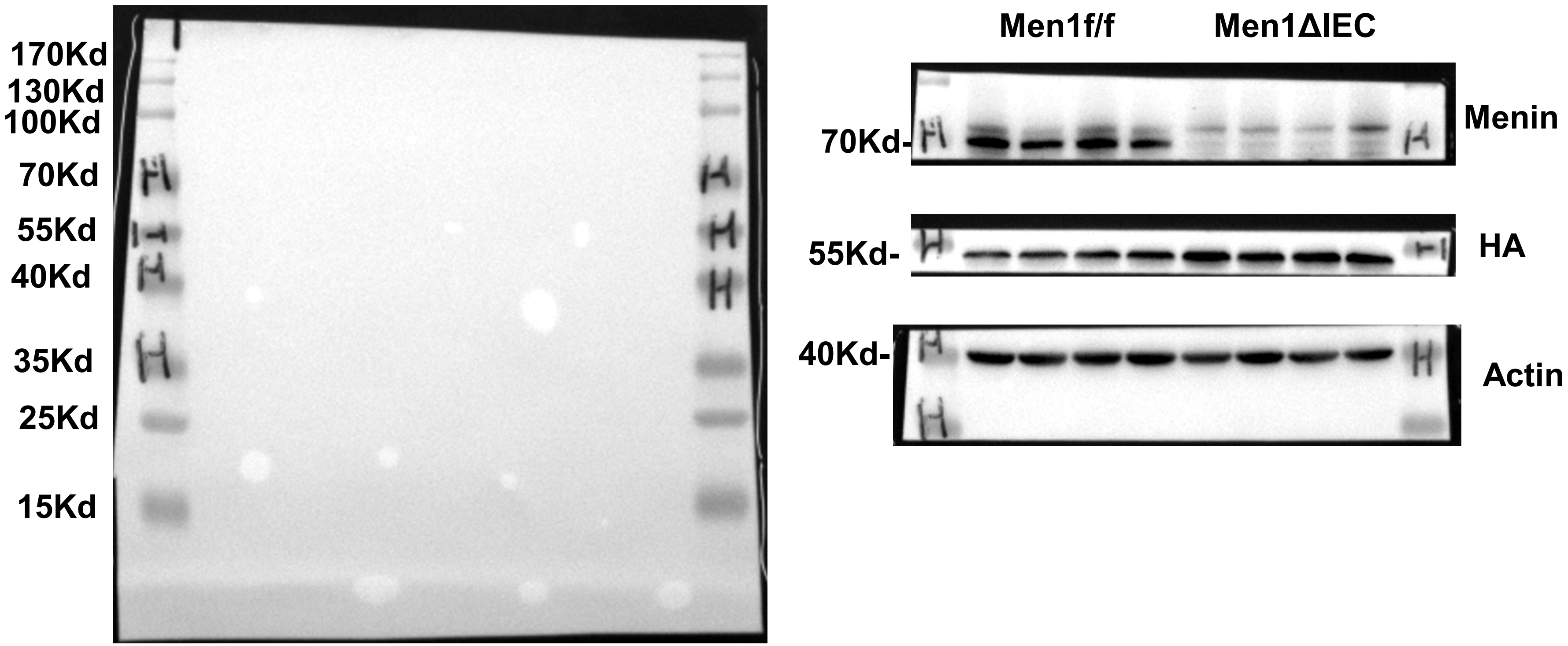
170Kd
130Kd
100Kd
70Kd
55Kd
40Kd
35Kd
25Kd
15Kd
Men1f/f
Men1ΔIEC
70Kd-
Menin
55Kd-
HA
40Kd-
Actin

Figure6I

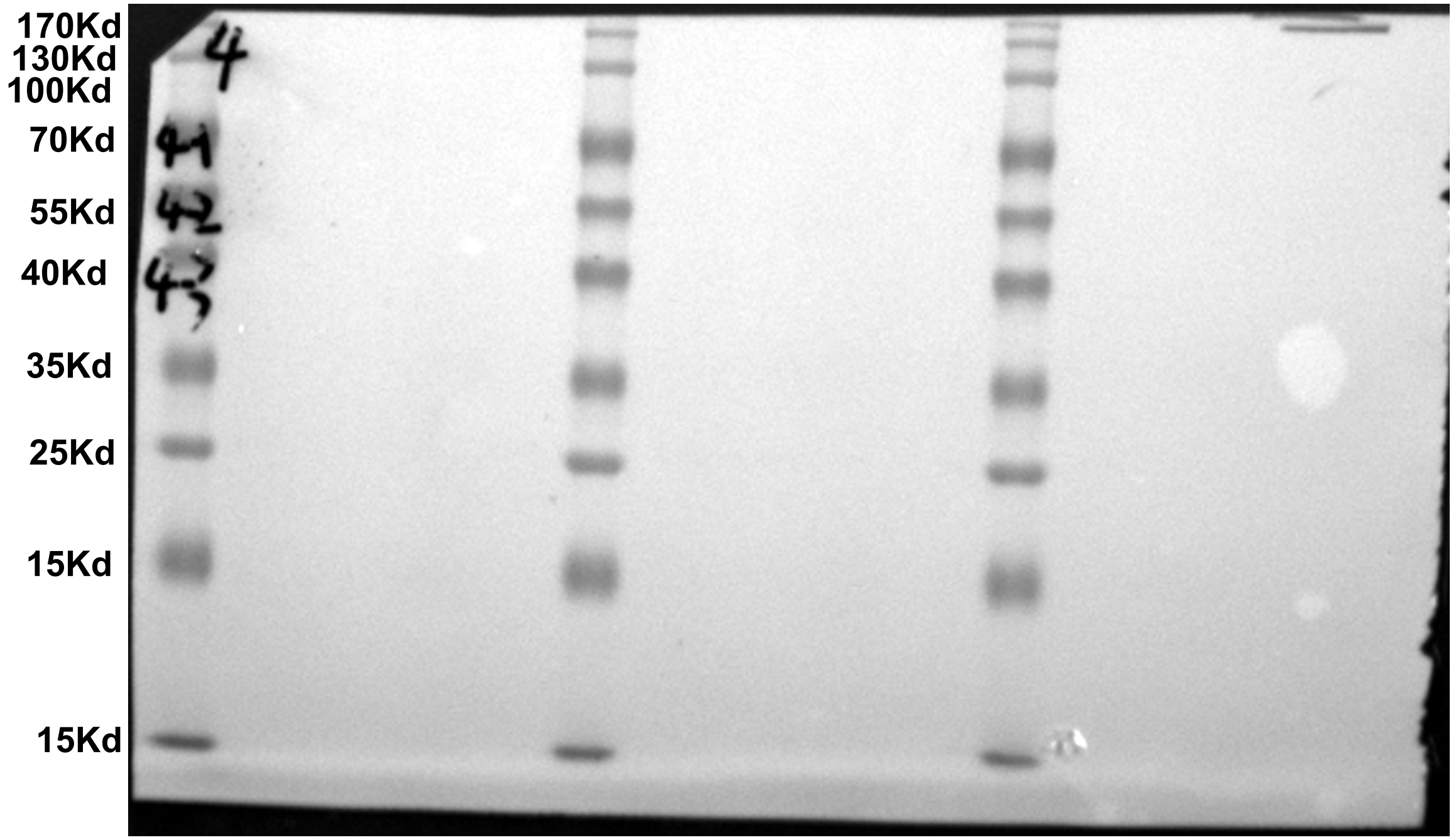

170Kd
130Kd
100Kd
70Kd
55Kd
40Kd
35Kd
25Kd
15Kd
15Kd

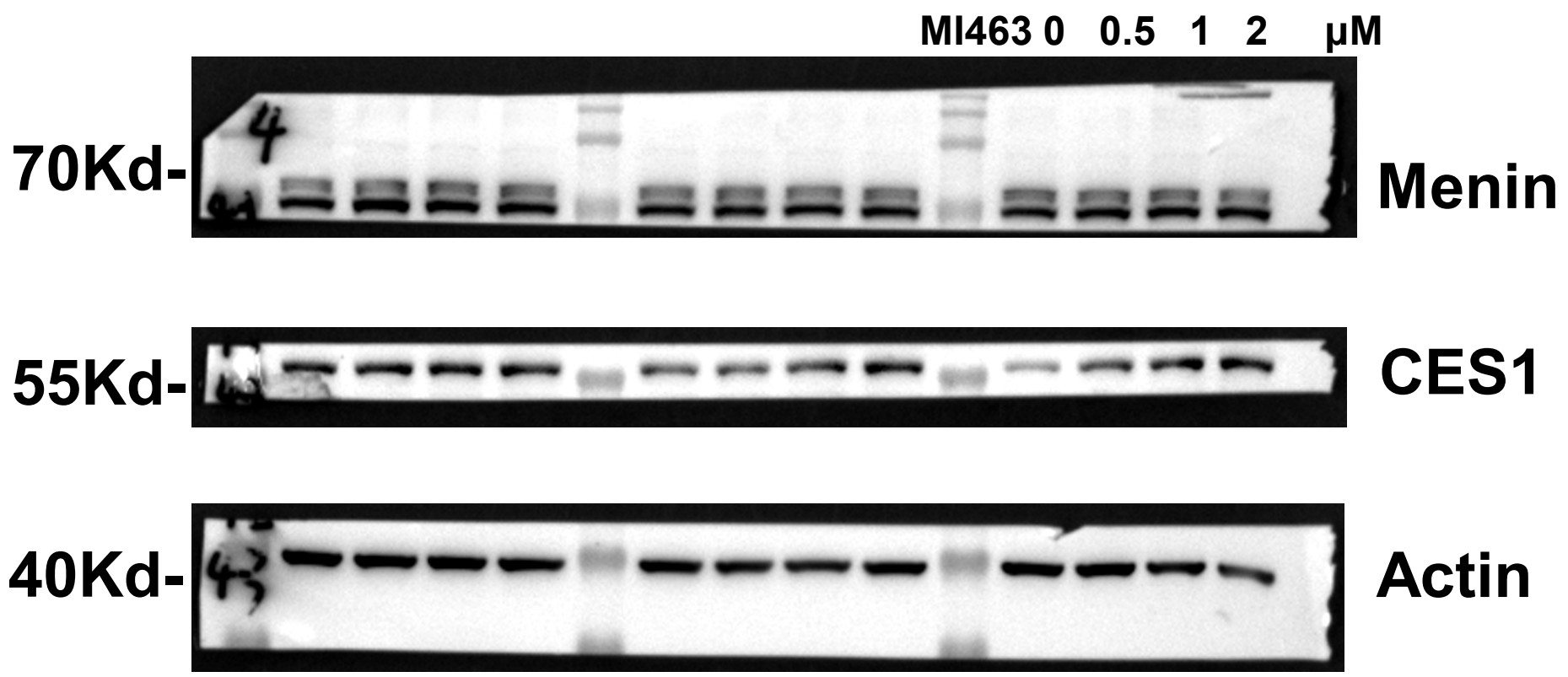

MI463 0 0.5 1 2 μM
70Kd-
Menin
55Kd-
CES1
40Kd-
Actin

Figure6J

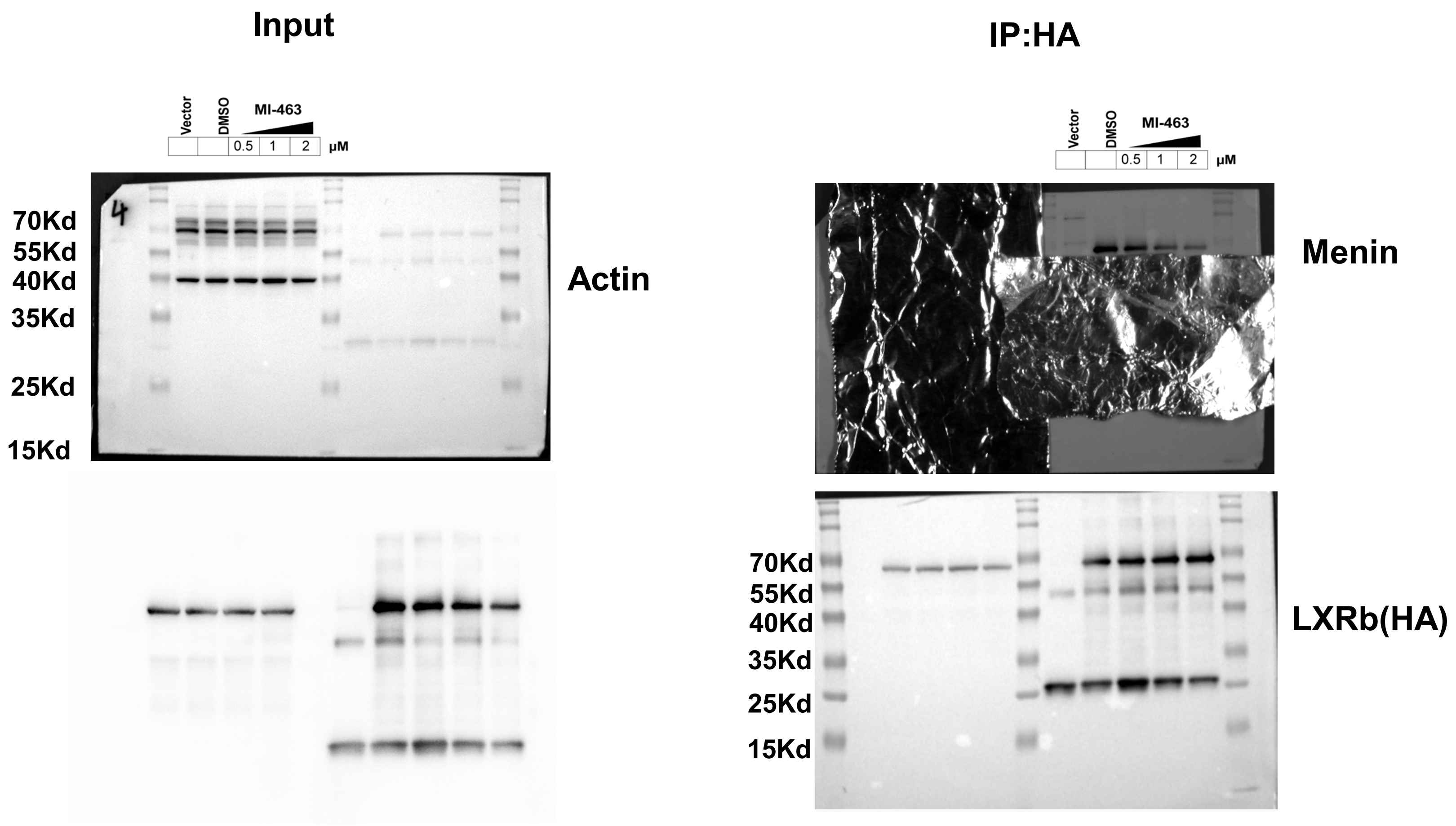
Input
Vector
DMSO
MI-463
0.5
1
2
μM
70Kd
55Kd
40Kd
35Kd
25Kd
15Kd
Actin
IP:HA
Vector
DMSO
MI-463
0.5
1
2
μM
Menin
70Kd
55Kd
40Kd
35Kd
25Kd
15Kd
LXRb(HA)

Figure6S-right

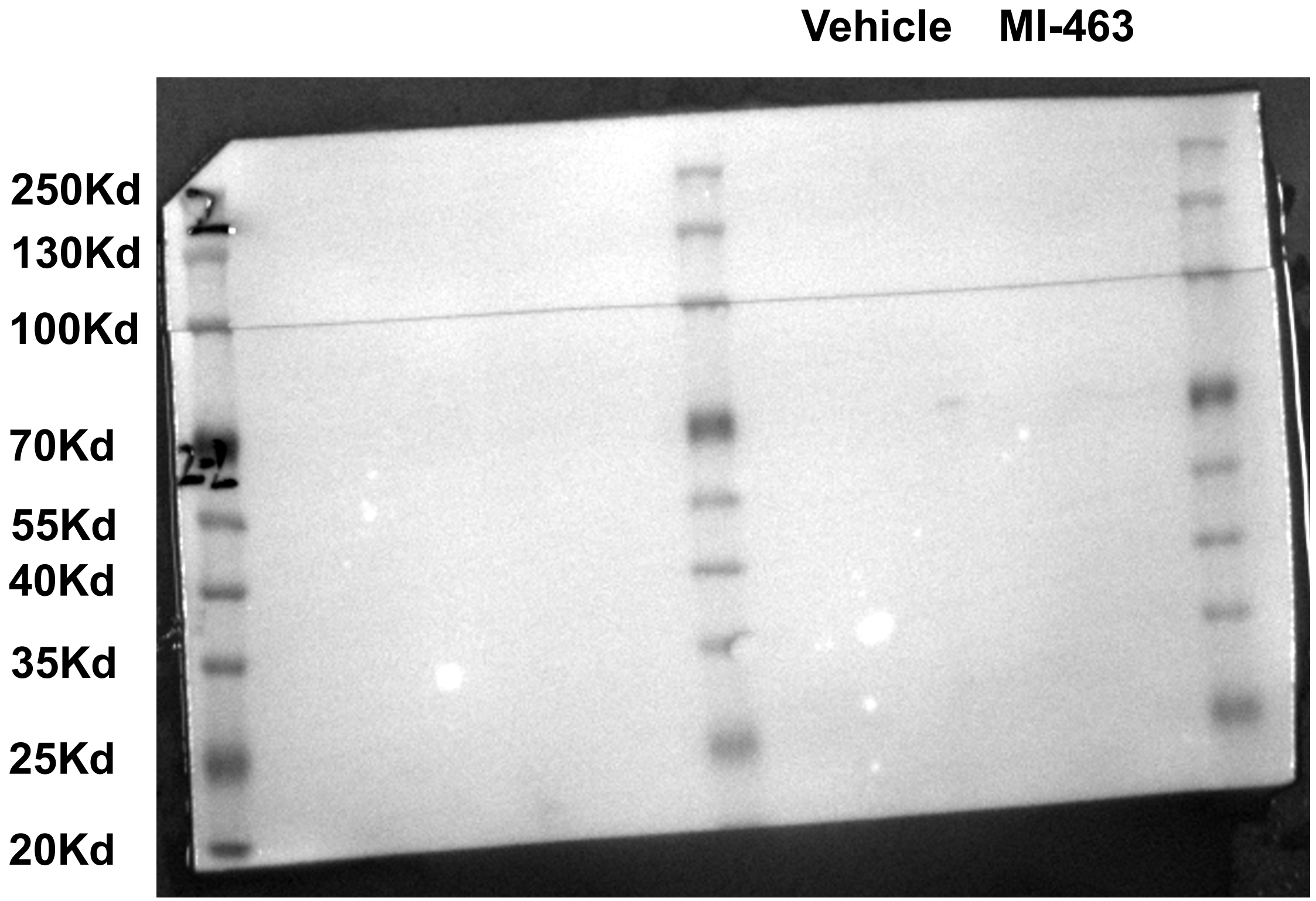

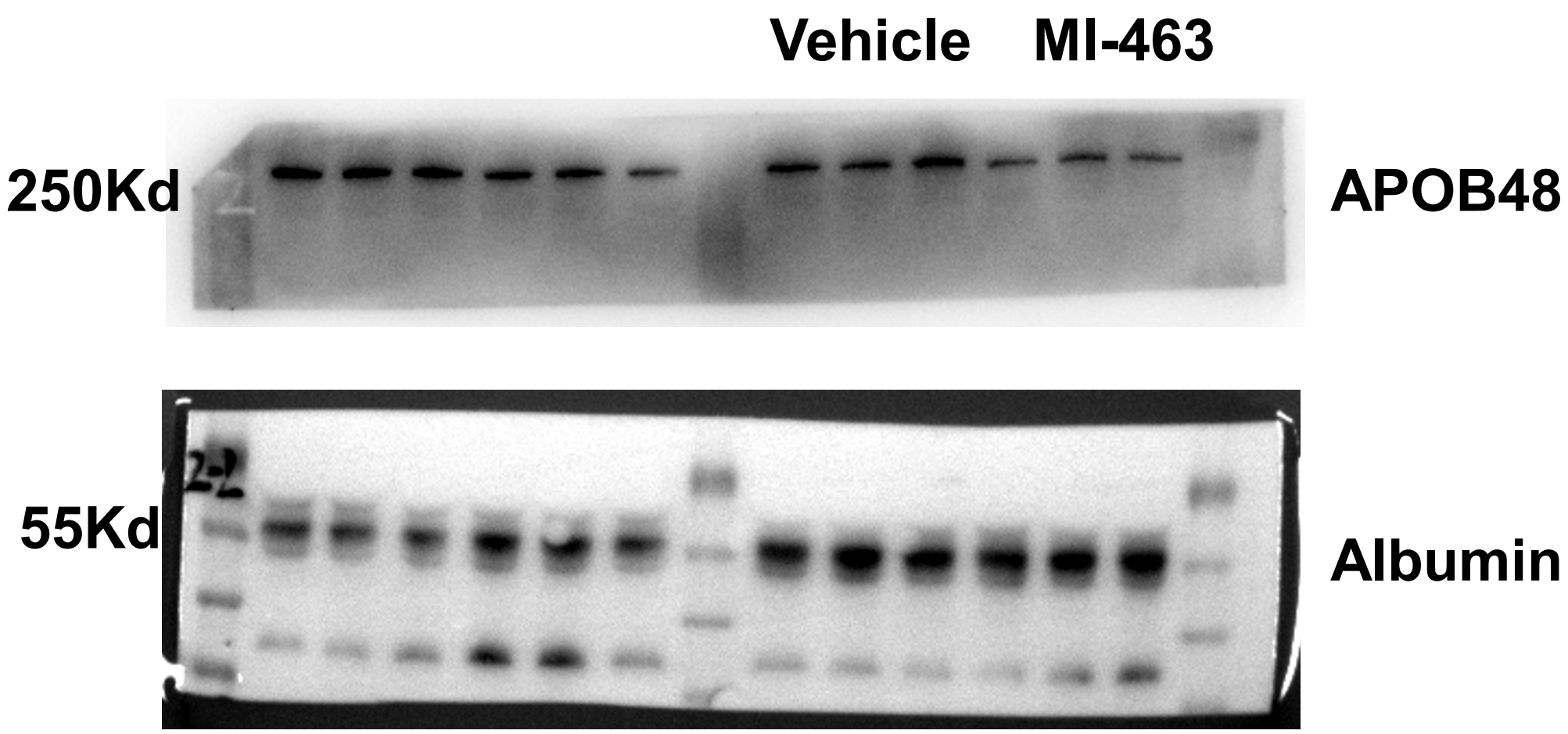

sFigure1E-left

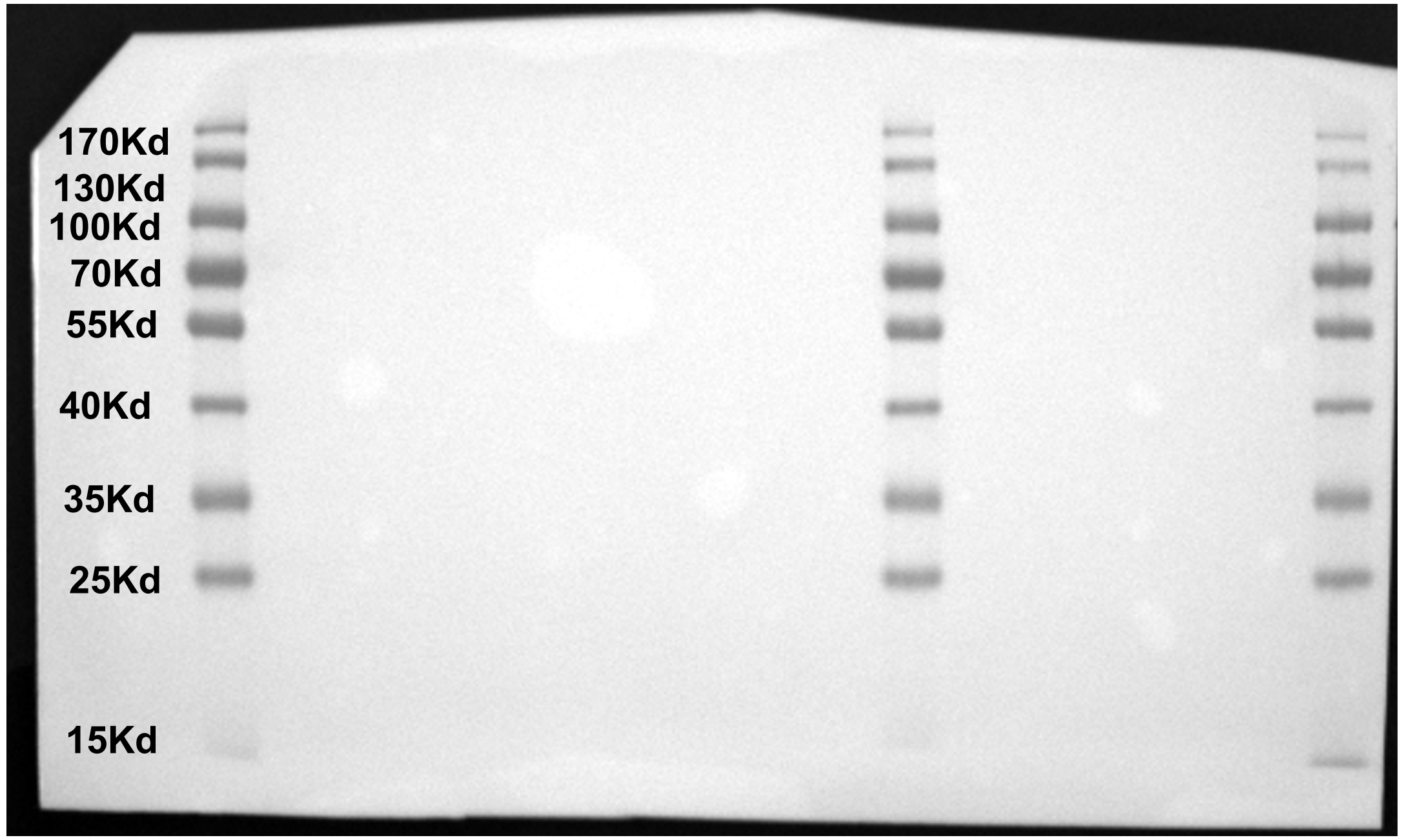

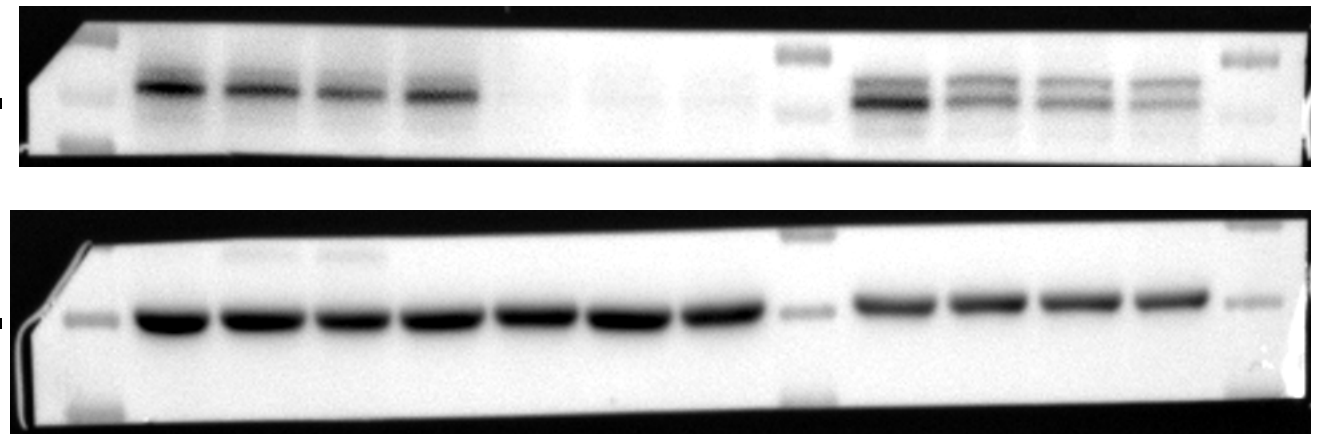

sFigure3F

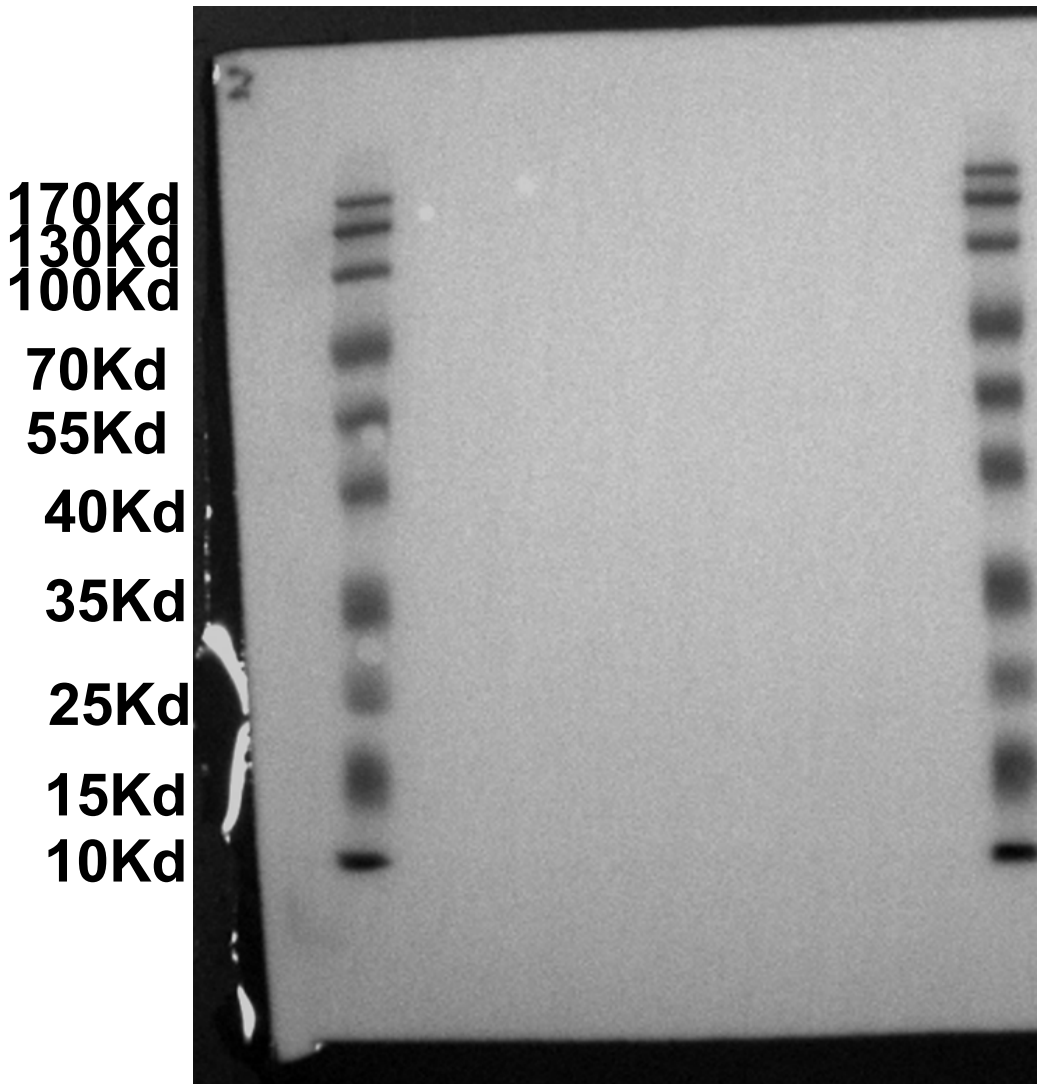

170Kd
130Kd
100Kd
70Kd
55Kd
40Kd
35Kd
25Kd
15Kd
10Kd

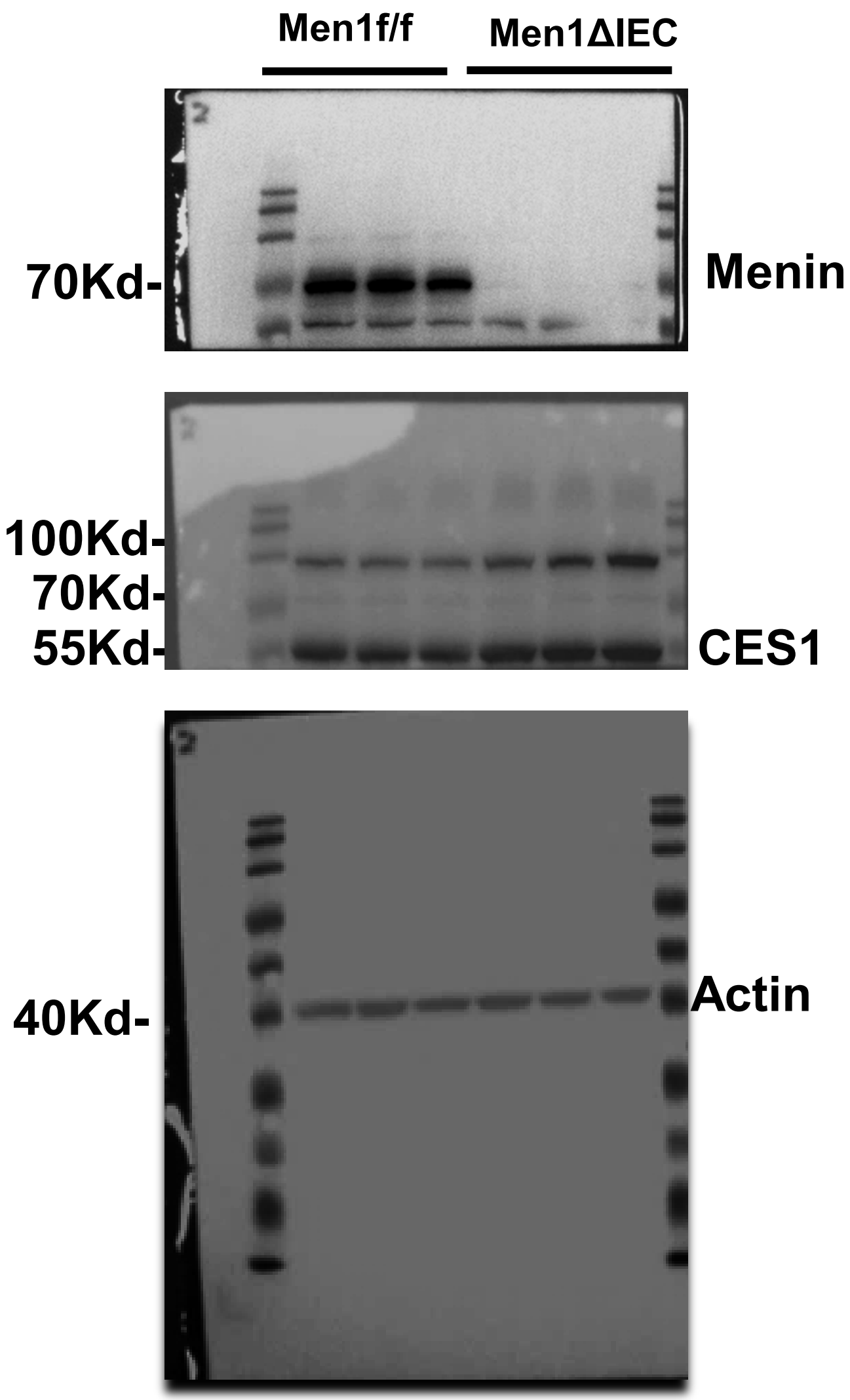

Men1f/f
Men1ΔIEC
70Kd-
Menin
100Kd-
70Kd-
55Kd-
CES1
40Kd-
Actin

SFigure3L

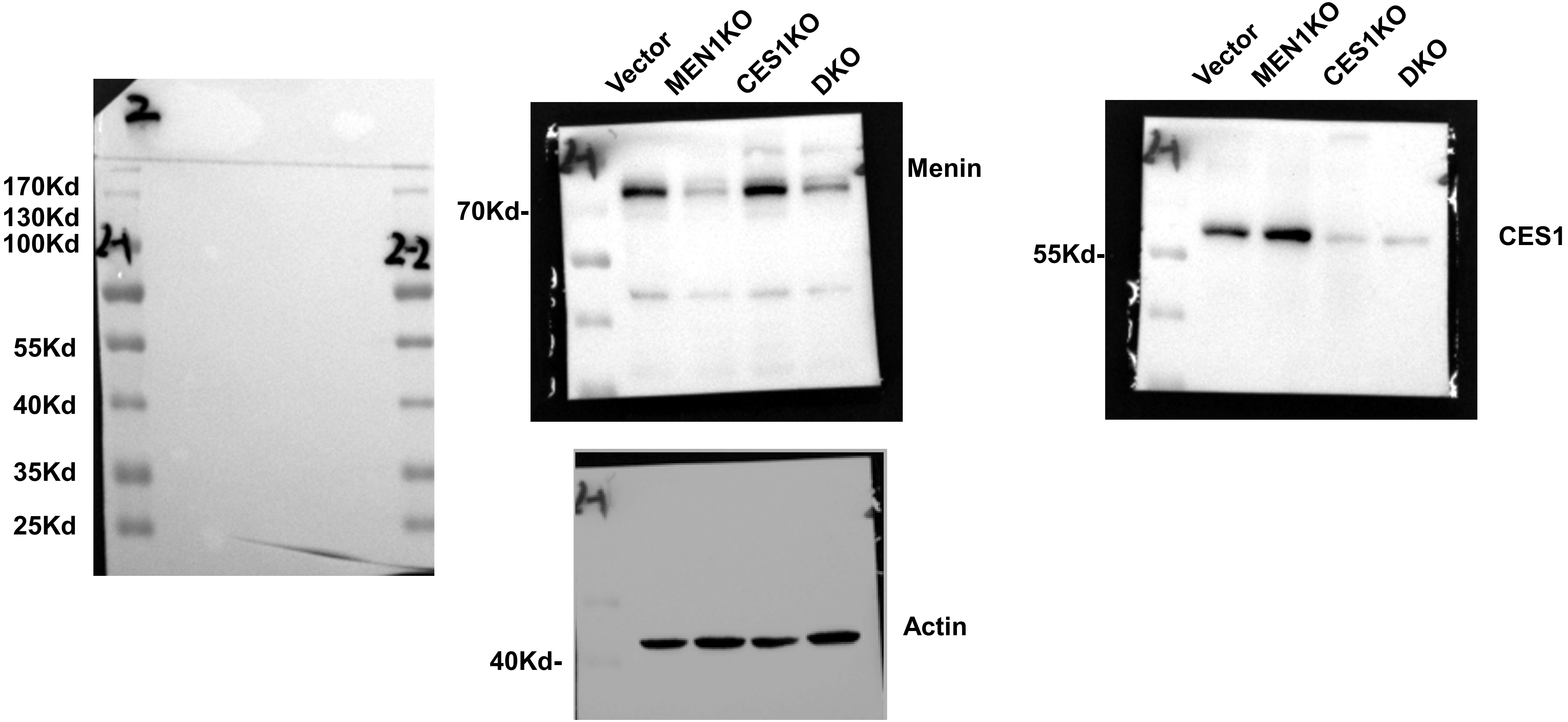

170Kd
130Kd
100Kd
55Kd
40Kd
35Kd
25Kd
Vector
MEN1KO
CES1KO
DKO
Menin
70Kd-
Actin
40Kd-
Vector
MEN1KO
CES1KO
DKO
CES1
55Kd-

SFigure4B

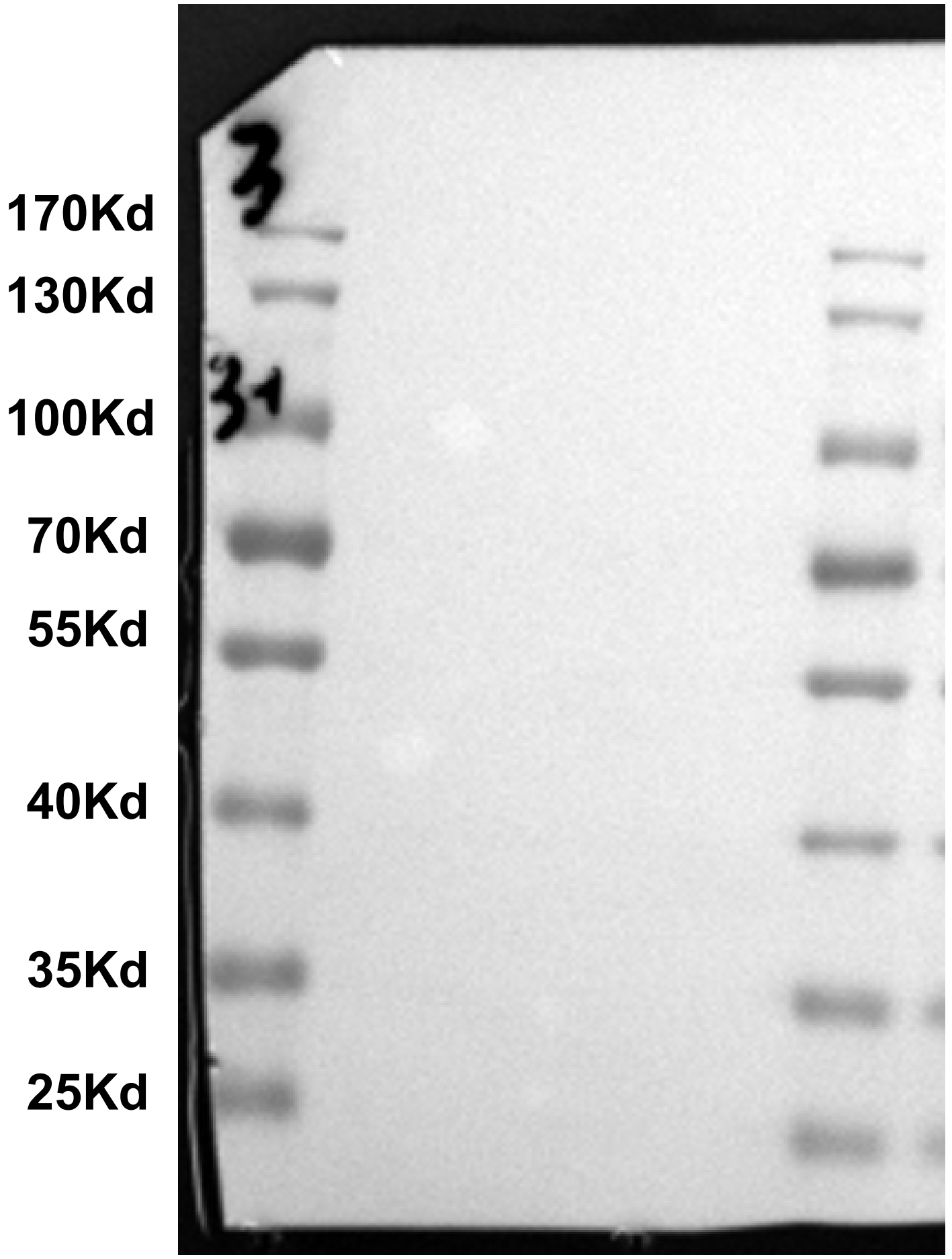
170Kd
130Kd
100Kd
70Kd
55Kd
40Kd
35Kd
25Kd

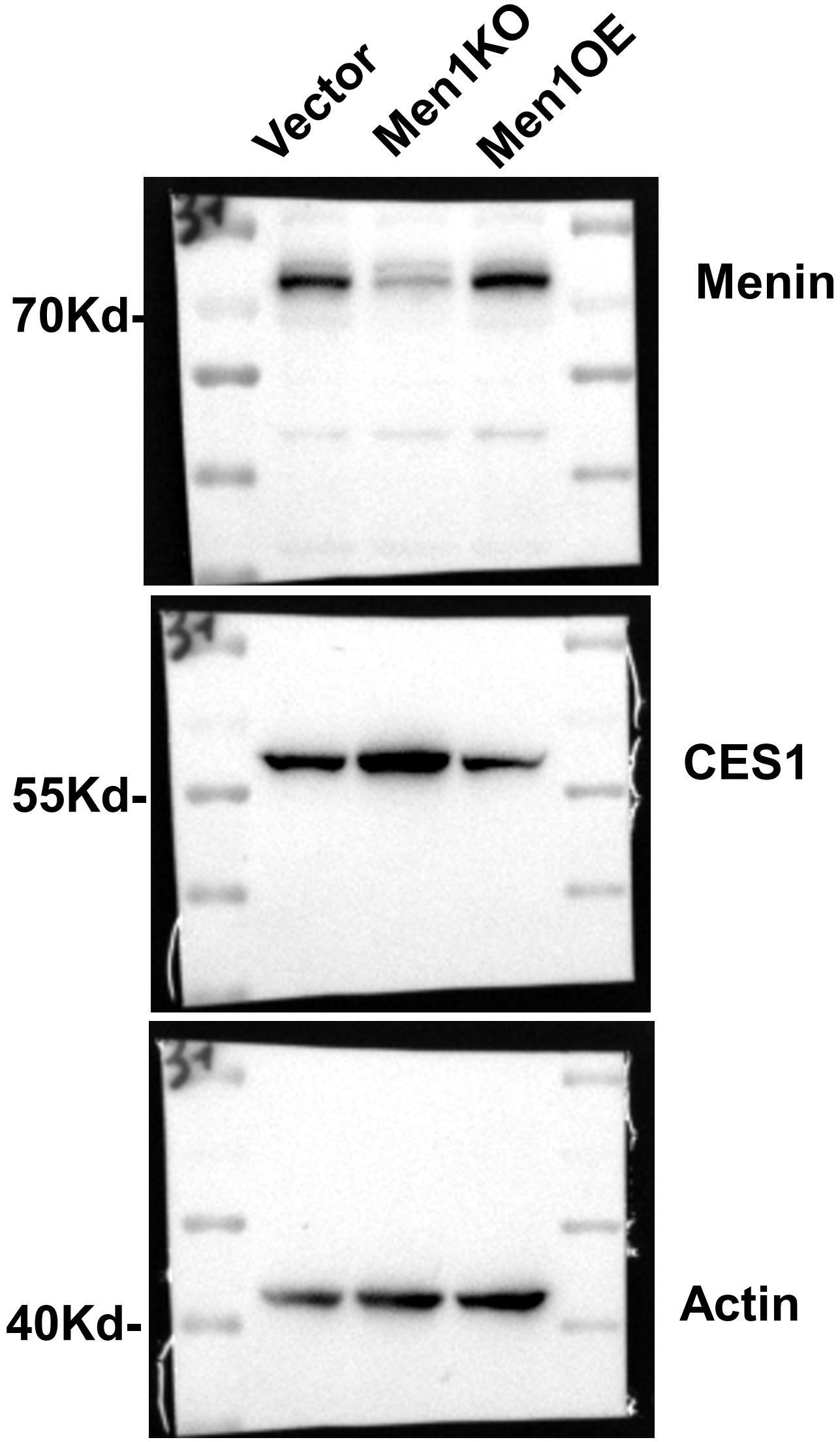
Vector
Men1KO
Men1OE
70Kd-
Menin
55Kd-
CES1
40Kd-
Actin

SFigure4G

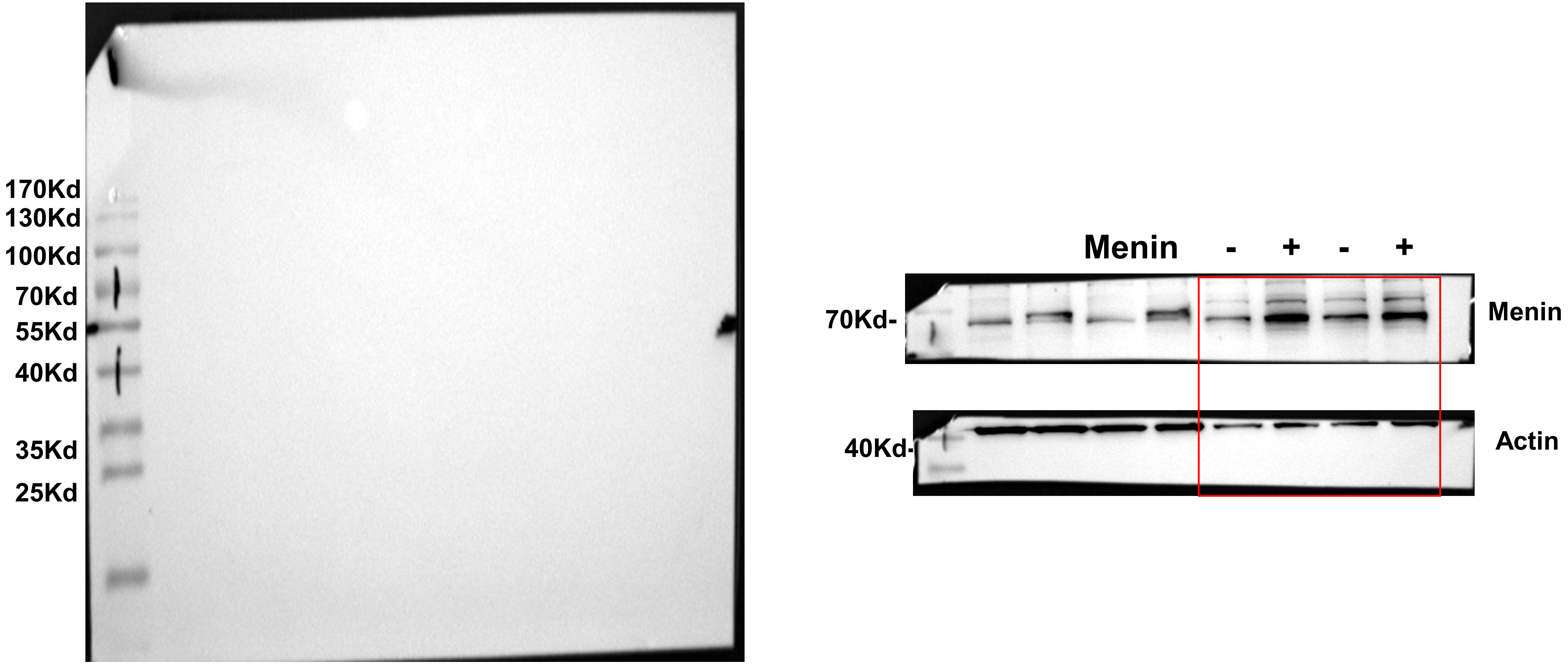
170Kd
130Kd
100Kd
70Kd
55Kd
40Kd
35Kd
25Kd
Menin - + - +
70Kd-
Menin
40Kd-
Actin

sFigure5C and 5F

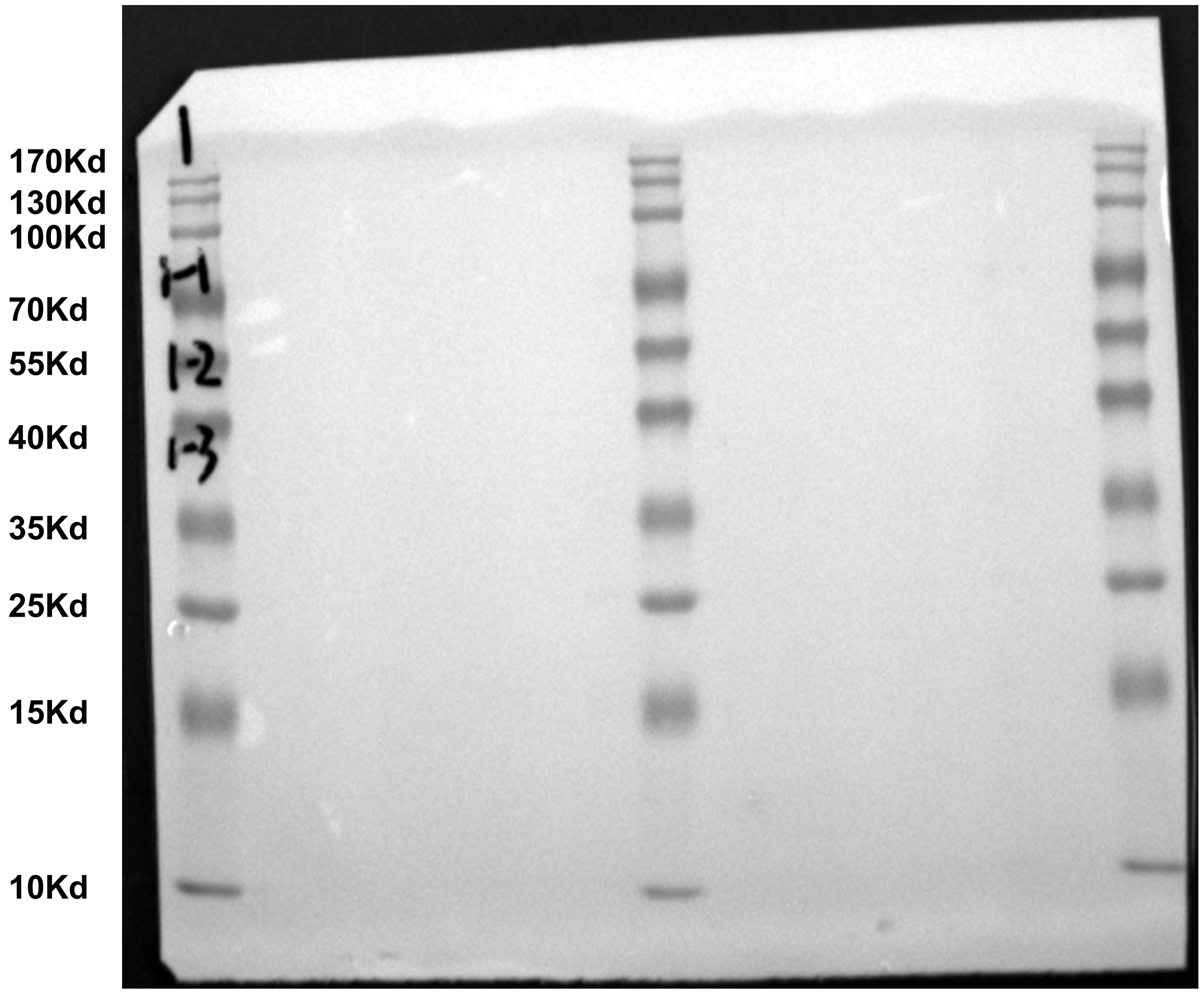

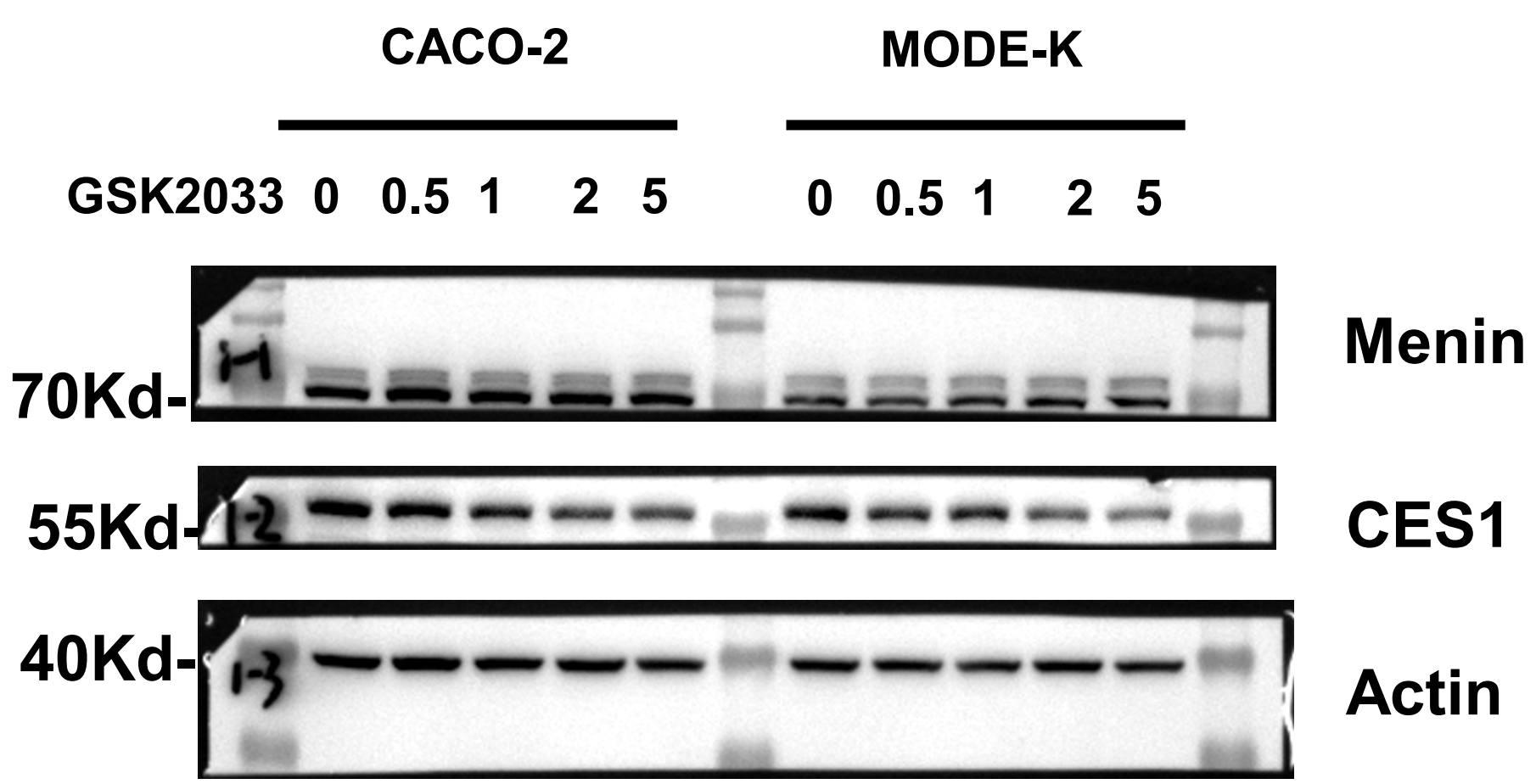

sFigure5H

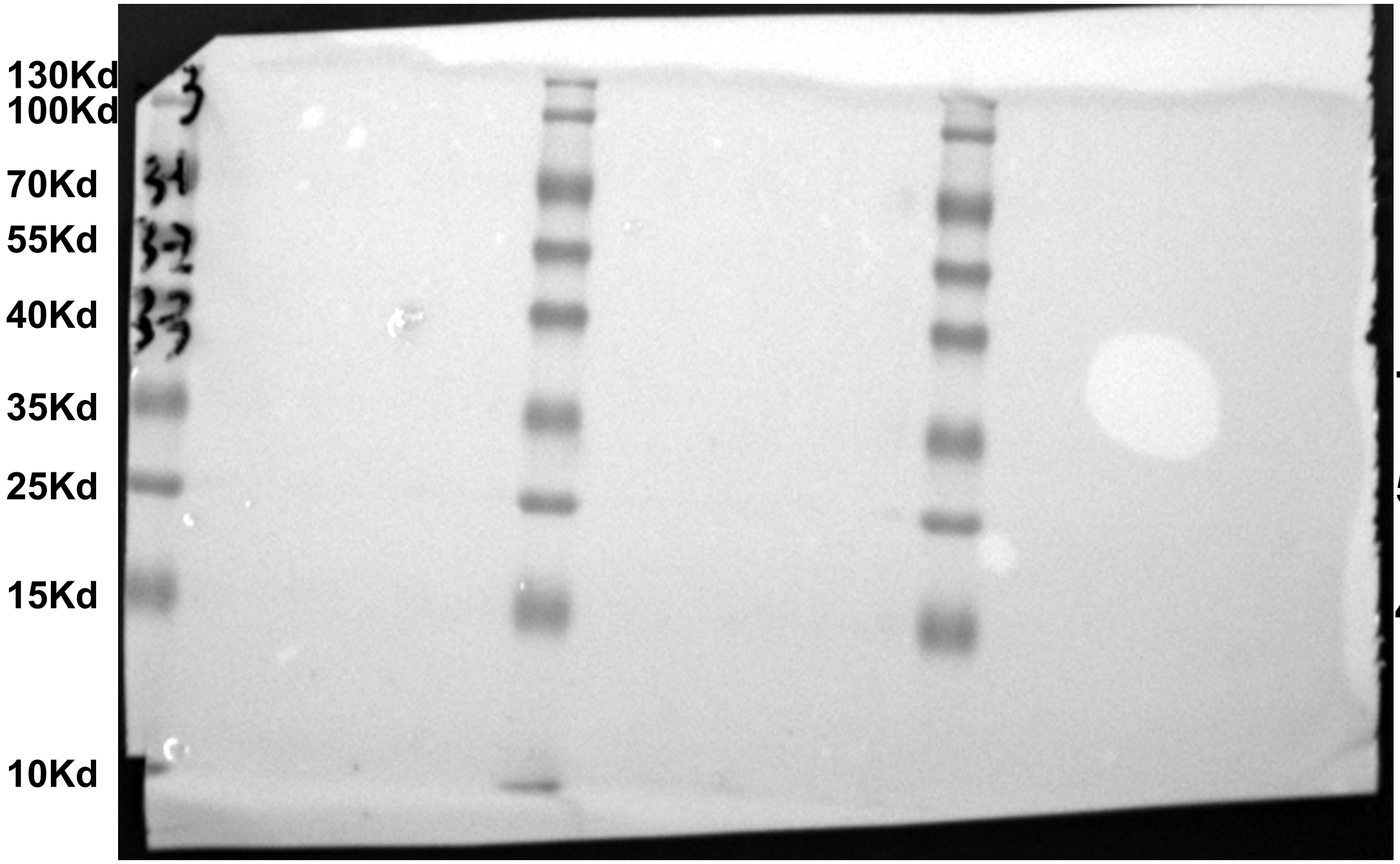
130Kd
100Kd
70Kd
55Kd
40Kd
35Kd
25Kd
15Kd
10Kd

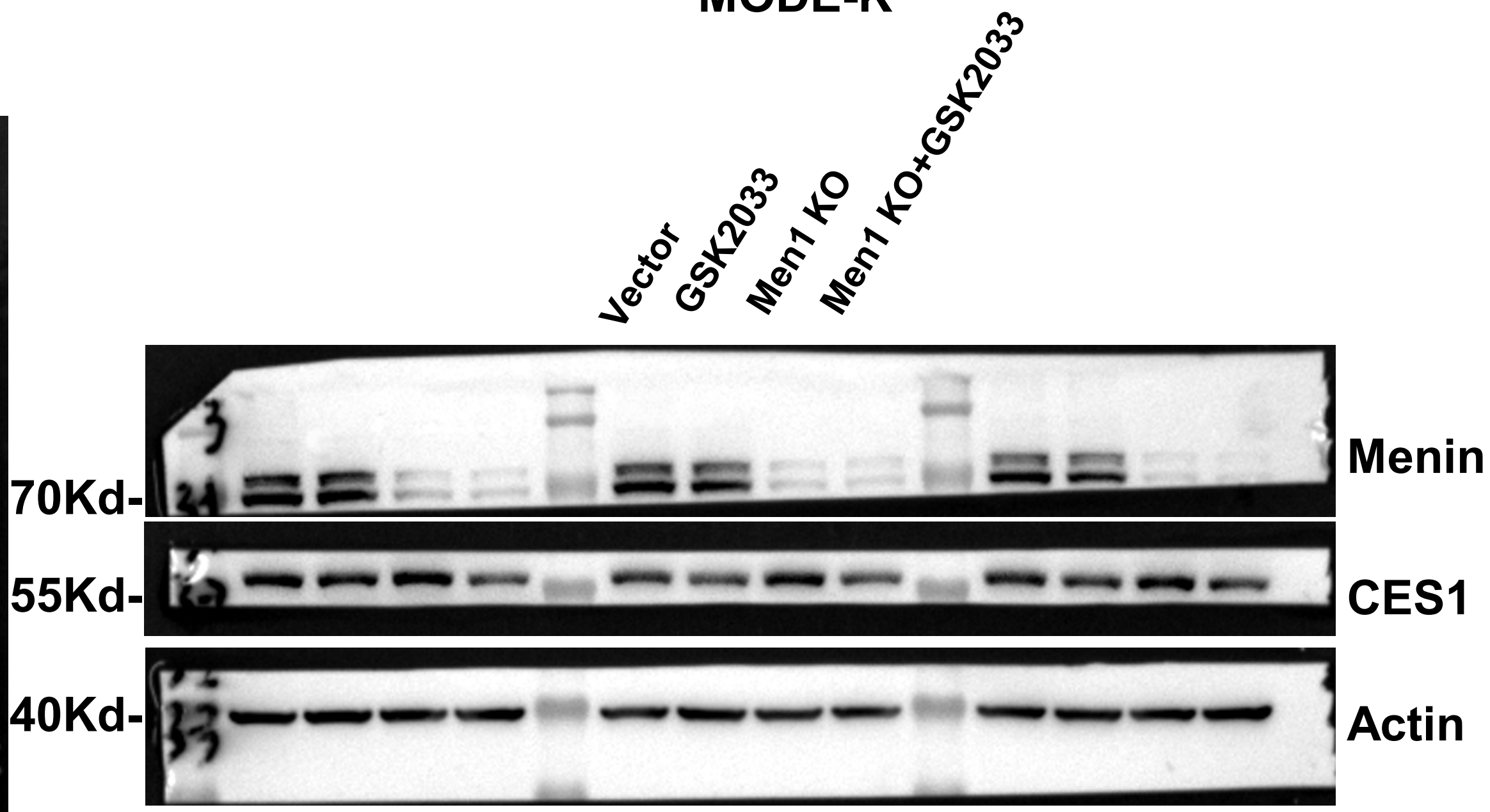
MODE-K
Vector
GSK2033
Men1 KO
Men1 KO+GSK2033
70Kd-
55Kd-
40Kd-
Menin
CES1
Actin

sFigure6B

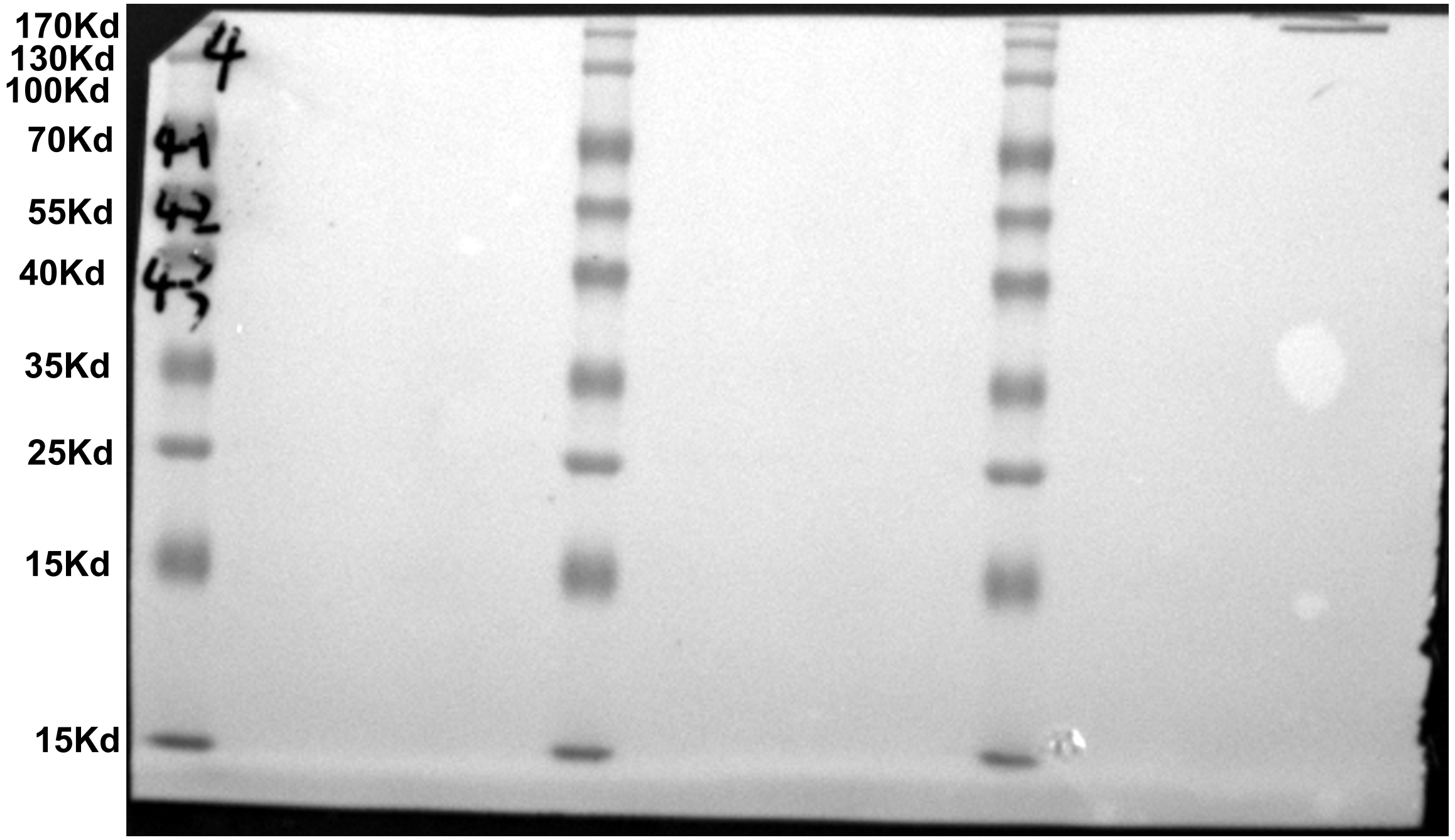

170Kd
130Kd
100Kd
70Kd
55Kd
40Kd
35Kd
25Kd
15Kd
15Kd

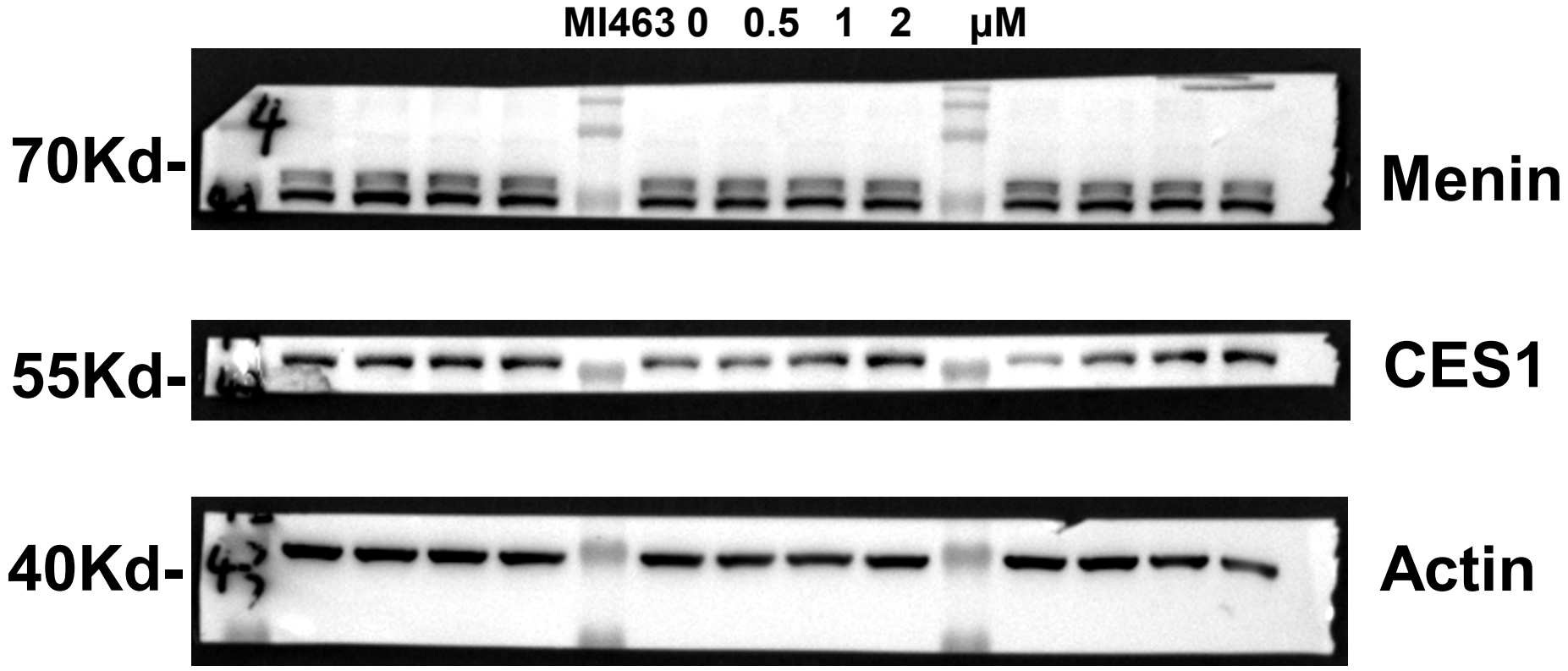

MI463 0 0.5 1 2 μM
70Kd-
Menin
55Kd-
CES1
40Kd-
Actin
